# Massively parallel barcode sequencing revealed the interchangeability of capsule transporters in *Streptococcus pneumoniae*

**DOI:** 10.1126/sciadv.adr0162

**Published:** 2025-01-24

**Authors:** Wan-Zhen Chua, Rachel Lyn Ee Wong, Ye-Yu Chun, Nicole Ng Chyi Shien, Tong Su, Matthias Maiwald, Kean Lee Chew, Raymond Tzer-Pin Lin, Alyson M. Hockenberry, Min Luo, Lok-To Sham

**Affiliations:** ^1^ Infectious Diseases Translational Research Programme and Department of Microbiology and Immunology, Yong Loo Lin School of Medicine, National University of Singapore, Singapore, Singapore.; ^2^ Department of Pathology and Laboratory Medicine, KK Women’s and Children’s Hospital, Singapore, Singapore.; ^3^ Duke-NUS Graduate Medical School, National University of Singapore, Singapore, Singapore.; ^4^ Department of Laboratory Medicine, National University Hospital, Singapore, Singapore.; ^5^ National Public Health Laboratory, Ministry of Health, Singapore, Singapore.; ^6^ Department of Microbiology and Immunology, Stritch School of Medicine, Loyola University Chicago, Chicago, IL, USA.; ^7^ Department of Biological Sciences, Faculty of Science, National University of Singapore, Singapore, Singapore.

## Abstract

Multidrug/oligosaccharidyl-lipid/polysaccharide (MOP) family transporters are essential in glycan synthesis, flipping lipid-linked precursors across cell membranes. Yet, how they select their substrates remains enigmatic. Here, we investigate the substrate specificity of the MOP transporters in the capsular polysaccharide (CPS) synthesis pathway in *Streptococcus pneumoniae*. These capsule flippases collectively transport more than 100 types of capsule precursors. To determine whether they can substitute for one another, we developed a high-throughput approach to systematically examine nearly 6000 combinations of flippases and substrates. CPS flippases fall into three groups: relaxed, type-specific, and strictly specific. Cargo size and CPS acetylation affect transport, and we isolated additional gain-of-function flippase variants that can substitute for the peptidoglycan flippase YtgP (MurJ). We also showed that combining flippase variants in a single cassette allows various CPS precursors to be flipped, which may aid glycoengineering. This study reveals that MOP flippases exhibit broad specificity, shaping the evolution of glycan synthesis.

## Teaser

About 6000 combinations of transporters and substrates were examined to determine the specificity of capsule transporters.

## INTRODUCTION

Virtually all cell surfaces are covered with glycans. They have diversified roles, such as maintaining the structural integrity of the cell, facilitating cell-cell interactions, immunomodulation, and, for pathogens, evading the host’s immune responses. In many cases, the biosynthetic pathway of glycans spans across a biological membrane. Thus, a conduit is often required to overcome the energy barrier of trafficking (or “flipping”) the hydrophilic glycan precursors through the hydrophobic lipid bilayer. This process is usually mediated by transporters of the multidrug/oligosaccharidyl-lipid/polysaccharide (MOP) superfamily, which includes the cell wall peptidoglycan (PG) flippase MurJ (*1*–*4*), the O-antigen flippase Wzx (*5*, *6*), and the eukaryotic N-linked protein glycosylation flippase Rft1 (*7*). In addition, MOP transporters in the multidrug and toxin compound extrusion family flippases are involved in the efflux of antibiotics, therapeutic drugs, and toxic molecules (*4*, *8*, *9*).

**Fig. 1.**
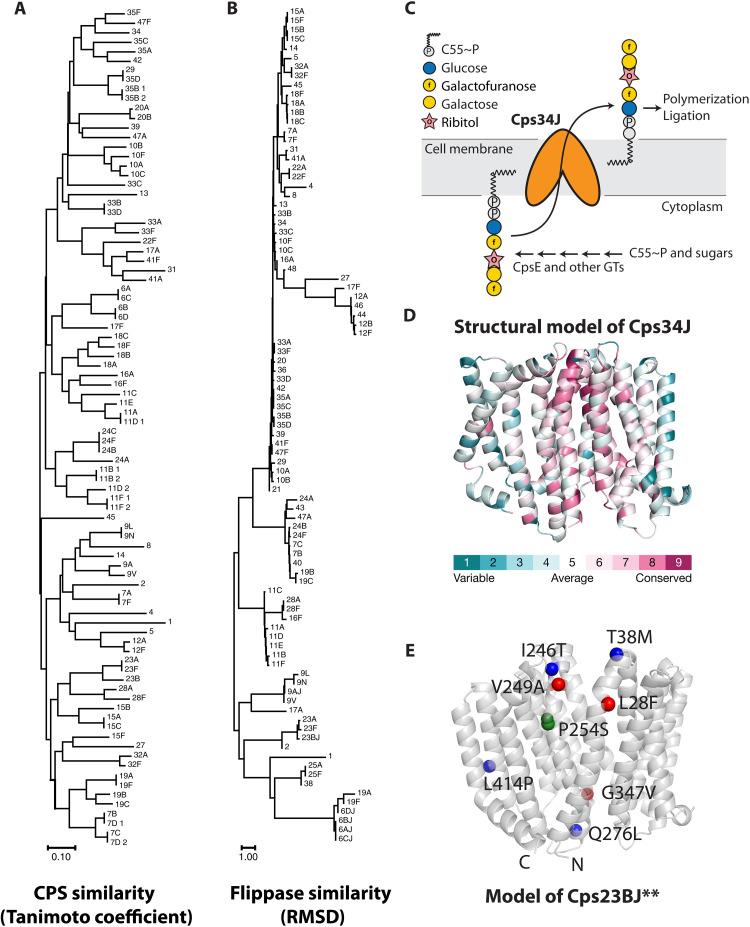
Diversity of the capsule flippases and their substrates. (**A**) Structures of the CPS repeating units were retrieved from the carbohydrate structure database (*52*). Pairwise comparisons were performed by calculating their Tanimoto coefficient. Hierarchical clustering depicts the differences in their chemical structures. (**B**) Structural models of CpsJ generated by AlphaFold (*47*). Phylogenetic trees were constructed with MEGA (*50*) by calculating the root mean square deviation (RMSD) of the CpsJ models. (**C**) Synthesis of the serotype 34 capsule. The repeating unit is assembled on the lipid carrier undecaprenyl phosphate (Und-P) in the cytoplasm. Sugar residues are sequentially added by activities of the polyprenol phosphoglycosyl transferase (PGT) CpsE and glycosyltransferases (GTs). The completed repeat units are transported across the cell membrane by Cps34J before polymerization and ligation to the peptidoglycan. (**D**) The structural model of Cps34J is predicted by AlphaFold, showing two pseudosymmetrical bundles of transmembrane helices, which adopt an inward-facing configuration and are colored based on their sequence conservation. Variable and conserved regions are colored in cyan and magenta, respectively. (**E**) cps23BJ** alleles identified in this study. *cps23BJ*(P254S) was mutagenized and introduced into strain NUS1992 [P_Zn_-*ytgP* ∆*ytgP*::P-*erm*] to identify mutant alleles that could substitute for the function of *ytgP* (the peptidoglycan lipid II flippase in *S. pneumoniae*). The P254S mutation is highlighted in green. Amino acid changes that relax the specificity of Cps23BJ are colored blue (*30*), while previously unidentified residues are shown in red. See also fig. S1.

In *Streptococcus pneumoniae*, MOP transporters support the production of capsular polysaccharides (CPS) (*10*–*12*), a major virulence factor that protects the bacteria from mucociliary clearance, complement deposition, and opsonophagocytosis (*10*, *13*–*15*). The pneumococcal CPS is remarkably diverse, with 107 serotypes identified to date (*10*, *16*–*18*). Except for serotypes 3 and 37, *S. pneumoniae* uses the Wzx/Wzy-dependent pathway for capsule synthesis (*11*). In this pathway, repeating units of CPS precursor are assembled on a lipid-carrier undecaprenyl phosphate (Und-P). Next, the MOP flippase CpsJ (Wzx) facilitates the transport of the lipid-linked precursor before it is polymerized by the CpsH (Wzy) polymerase and ligated to the PG (*19*) (Fig. 1). Und-P is then released and recycled for the next round of synthesis. Interruptions in the completion of this pathway are often lethal, as they result in the sequestration of lipid-linked precursors and stall the synthesis of other glycans, such as PG (*2*, *20*, *21*).

As the capsule flippases transport a variety of substrates (Fig. 1A), they are known to have great structural diversity (Fig. 1B) (*22*, *23*). Despite extensive research to elucidate their transport mechanism, how they recognize their substrates remains unclear. Sequence alignments and structural modeling could not identify residues that contribute to specificity in the substrate-binding pocket. Yet, the mechanism of how MOP flippases transport their substrates appears to be conserved based on their structures (*4*, *24*–*26*). Overall, MurJ and other MOP flippases have two pseudosymmetrical bundles of transmembrane helices that adopt an overall V-shape configuration, forming a central cavity that may be involved in substrate binding (Fig. 1, C and D). Substrate binding triggers conformational changes that likely lead to a transition from an inward-open to an outward-open state. This transition is thought to release the substrates to the outer leaflet of the cytoplasmic membrane (*4*, *24*–*26*). Because of this, mutations that break the contacts of the extracellular gate presumably destabilize the inward-facing conformation and reduce substrate specificity (*27*, *28*, *21*). To better understand the specificity determinant of MOP flippases, we previously examined whether *cpsJ* alleles in different serotypes are interchangeable (*21*). Similar to the residues that determine substrate selectivity, our results suggest that sequence similarity cannot be used to predict whether the *cpsJ* alleles can substitute for each other. In addition, subtle changes in the central aqueous cavity of the transporter are shown to be sufficient to alter specificity (*21*). While this study involves 177 CpsJ-switch mutants across 82 serotypes, we have yet to fully understand why some CpsJ flippases can transport many noncognate cargos, whereas others seem strictly specific.

Here, we developed a high-throughput approach to systematically assess the interchangeability of more than 6000 combinations of CpsJ flippases and their substrates. This was done by combining molecular barcoded *cpsJ* alleles and our collection of CPS-switch mutants (*21*, *29*). Through sequencing the barcodes by next-generation sequencing (i.e., Bar-seq), the fitness of strains harboring various combinations of CpsJ and capsule cargos was simultaneously measured. We showed that the specificity of CpsJ flippases varies substantially, ranging from strictly specific flippases to one that could transport nearly half of the 79 cargos tested. The size of the CPS precursors and acetylation were found to play an essential role in substrate selection. CpsJ variants that acquired the ability to flip the PG precursor lipid II exhibited a relaxed specificity, enabling the transport of various CPS precursors. We demonstrated that combining three flippases into a “TRIO” cassette allowed us to complement multiple noncognate CPS flippases. Last, we observed that flippases with relaxed specificity were toxic to the cells, which could explain the selective pressure that maintains the specificity of MOP transporters.

## RESULTS

### Isolation of capsule flippase variants that could transport PG precursors

Previously, we showed that capsule flippases can be mutagenized to gain the ability to flip noncognate substrates (*21*, *28*, *30*). The amino acid changes mostly congregate around the extracellular gate formed by the transmembrane helices. To test whether other substitutions can lead to a reduction in substrate specificity, we mutagenized *cps23BJ*(P254S), a mutant *cpsJ* allele from serotype 23B that gained the ability to flip the serotype 2 precursor, to isolate alleles that could also flip lipid II (*21*, *30*). This was done by PCR mutagenesis of the *cps23BJ*(P254S) allele at the ectopic β-galactosidase (*bgaA*) locus [∆*bgaA::P-kan-cps23BJ(P254S)*] and selecting for gain-of-function mutants that survived when the essential lipid II flippase *ytgP* (*murJ*) was depleted (fig. S1). For convenience, we collectively referred to these double mutants as cps23BJ**. Previously, we identified four cps23BJ** alleles (T38M, I246T, Q276L, and L414P in addition to P254S) (*30*). Here, we isolated three additional cps23BJ** alleles (P254S with L28F, V249A, or G347V) that could complement *ytgP* (Fig. 1E). We reconstructed these alleles by site-directed mutagenesis and demonstrated that they remained functional and flipped lipid II when they were transformed into a strain with an inducible copy of *ytgP*, NUS1992 [∆CEP::P_Zn_-*ytgP* ∆*ytgP*::P-*erm*] (fig. S1). To examine the spectrum of substrates by which Cps23BJ** can transport, we tested whether Cps23BJ** could complement Cps2J, Cps14J, Cps23BJ, and TacF (the teichoic acid flippase). To test this, cps23BJ** alleles were first introduced ectopically [∆*bgaA*::P-*kan*-cps23BJ**] into isogenic capsule-switch mutants of serotypes 2, 14, and 23B (*29*) before *cpsJ* or *tacF* at the native locus was deleted. None of the Cps23BJ** variants support the translocation of teichoic acid precursors, as transforming the ∆*tacF*::P-*erm* cassette generated no viable *tacF* deletion mutant, suggesting that these Cps23BJ** variants could not substitute TacF (table S1). Nevertheless, they could complement Cps2J and Cps23BJ (fig. S1 and tables S2 and S3), demonstrating that their specificity was relaxed instead of switched. In addition, the degree of specificity relaxation differs, as all cps23BJ** alleles except *cps23BJ*(P254S G347V) could transport the serotype 14 substrate (fig. S1 and table S4). As reported in other Wzx flippases (*5*), the overexpression of a noncognate flippase may cause the translocation of unrelated substrates. To rule out this possibility, we fused them with a FLAG tag (DYKDDDDK) at the C terminus and quantified their relative amounts by immunoblotting. The cellular amount of Cps23BJ(P254S G347V) remained the same, whereas the amounts of Cps23BJ(P254S L28F), Cps23BJ(P254S V249A), and Cps23BJ(P254S L414P) increased by more than twofold compared to the wild type (fig. S1). This suggests that these three mutations may lead to the accumulation of the flippase in the cell. Cps23BJ(P254S Q276L) mildly accumulated in the cell (<2-fold). Together with G347V, Q276L is located near the intracellular gate and the central cavity (Fig. 1E), illustrating that there are likely different mechanisms to relax the specificity of MOP family flippases other than destabilizing the outward-facing conformation by changes at the extracellular gate (*21*).

### A high-throughput approach for investigating the interchangeability of capsule flippases

**Fig. 2.**
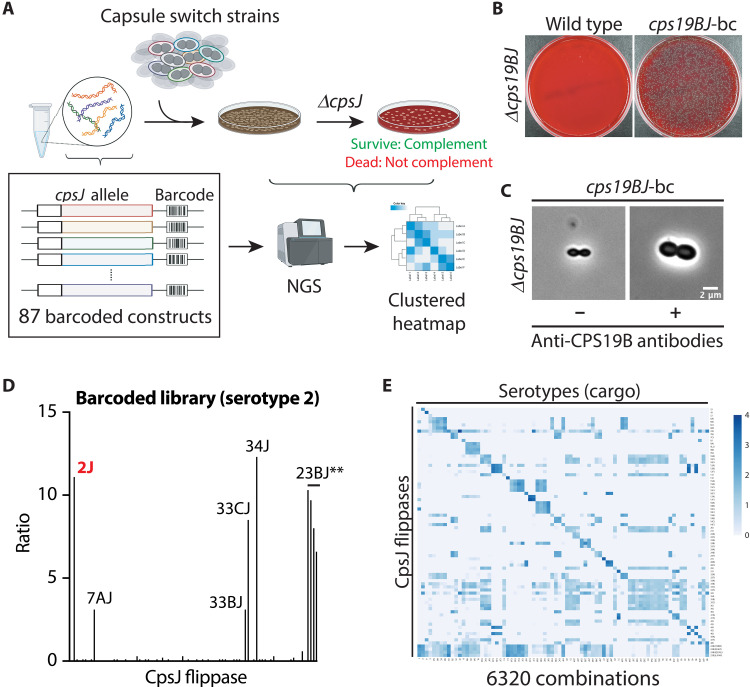
Interchangeability of capsule transporters. (**A**) A high-throughput approach to examine cross-complementation of capsule transporters. In total, 87 *cpsJ* alleles were cloned upstream of a DNA barcode, and the constructs were pooled and used to transform 1 of the 79 capsule-switch mutants (*29*). The resulting transformants are merodiploid with two copies of *cpsJ* genes. Next, the native *cpsJ* flippase was deleted (∆*cpsJ*), and the survivors were expected to carry a CpsJ variant that could replace the native *cpsJ*. The survivors were pooled, and their genomic DNA was purified. The barcodes of the ectopic *cpsJ* constructs were then amplified and sequenced (Bar-seq). The number of reads recovered for each barcode represents the fitness of the mutant carrying the corresponding *cpsJ* allele. Cross-complementation of the flippases is illustrated by a clustered heatmap generated using Clustvis (*49*). (**B**) The barcoded *cpsJ* constructs are functional. Strains NUS0353 [Serotype 19B isogenic capsule-switch mutant; CPS19B] and NUS2944 [CPS19B P-*kan*-*cps19BJ*-barcode] were grown in BHI broth at 37°C in 5% CO_2_ until they reached the early exponential phase. Cultures were induced by competent stimulating peptides and transformed with the ∆*cps19BJ* cassette. Transformants were plated on blood agar plates and incubated overnight at 37°C in 5% CO_2_ before imaging. (**C**) Immunostaining with and without anti-serogroup 19 antisera confirmed the presence of CPS in the ∆*cps19BJ* // *cps19BJ*-barcode mutant. A positive Quellung reaction was shown on the right with a plus sign. Scale bar, 2 μm. (**D**) Bar-seq experiments confirmed the *cpsJ* alleles that could complement *cps2J* (*21*). (**E**) Shown is a heatmap depicting the interchangeability of *cpsJ* alleles. The blue color represents the natural log ratios of the NGS read counts after the *cpsJ* allele at the native locus was deleted. Shown in (B) and (C) are representative images from three biological replicates. See also figs. S2 to S4.

CpsJ is required for growth likely because its inactivation results in the accumulation of dead-end Und-PP–linked intermediates (*20*). Several unmutagenized *cpsJ* alleles were shown to be able to transport noncognate substrates (*21*). Yet, which *cpsJ* alleles could do so is still unknown. To test this, we developed a high-throughput approach to systematically evaluate the cross-complementation of CpsJ. First, we inserted a unique DNA barcode adjacent to each of the 83 *cpsJ* alleles from the different serotypes. The barcoded *cpsJ* was then introduced into an ectopic locus (chromosomal expression platform or CEP) and expressed under a constitutive promoter (P-*kan*) that drives the expression of the kanamycin-resistant marker [CEP::P-*kan*-*cpsJ*-barcode]. In addition, four of the Cps23BJ** variants (P254S T38M, P254S I246T, P254S Q276L, and P254S L414P) were included to assess their ability to transport other CPS cargos. These 87 *cpsJ* alleles were pooled and introduced at the same genetic locus by open-reading frame replacements into the isogenic capsule-switch mutants constructed previously (*29*) (Fig. 2A). This resulting “input” library contained merodiploid strains with two copies of *cpsJ*. *cpsJ* at the native locus of the respective serotype in the “input” library was then deleted by transforming the library with a PCR fragment containing the erythromycin-resistant gene (P-*erm*) flanked by the adjacent sequences of *cpsJ*. Under this condition, cells could not survive unless the ectopic *cpsJ* allele was able to compensate for the capsule flippase function (Fig. 2, B and C). The survivors on the plates, collectively termed the “output” library, are expected to carry the cognate *cpsJ* and noncognate *cpsJ* alleles at the ectopic locus, capable of complementing the deleted *cpsJ* allele.

These *cpsJ* alleles at the ectopic locus were identified and quantified using DNA barcode sequencing (Bar-seq). The number of reads was normalized to reads per million (RPM). The fitnesses of the mutants harboring different *cpsJ* alleles were inferred by the changes in the relative abundance of barcodes, represented by the ratios of the RPMs in the output library to the input library (Fig. 2A). If the ratio remained unchanged or increased, it suggested that the deletion mutant could survive with the ectopically expressed *cpsJ* allele. This screen allowed us to examine combinations of *cpsJ* alleles and substrates for potential cross-complementation events.

As a proof of concept, we transformed the prototypical serotype 2 strain D39W with the pooled *cpsJ* constructs, followed by deleting *cps2J*. The results agreed with the previous complementation study except for *cps10BJ* and *cps33DJ* (Fig. 2D) (*21*). We hypothesize that *cps10BJ* and *cps33DJ* could not be expressed ectopically. To confirm this speculation, we tested whether *cps33DJ* in the expression cassette could complement *cps33DJ* at the native locus in a serotype 33D capsule switch mutant. No viable colonies could be obtained when ∆*cps33DJ* was introduced into the serotype 33D background, regardless of whether the strain contained the barcoded *cps33DJ* cassette (table S5). Thus, this cassette is likely nonfunctional. We could not test the functionality of the *cps10BJ* cassette as we do not have the capsule-switch strain for serotype 10B. Thus, we excluded these alleles in this study as they may not be functional ectopically.

**Fig. 3.**
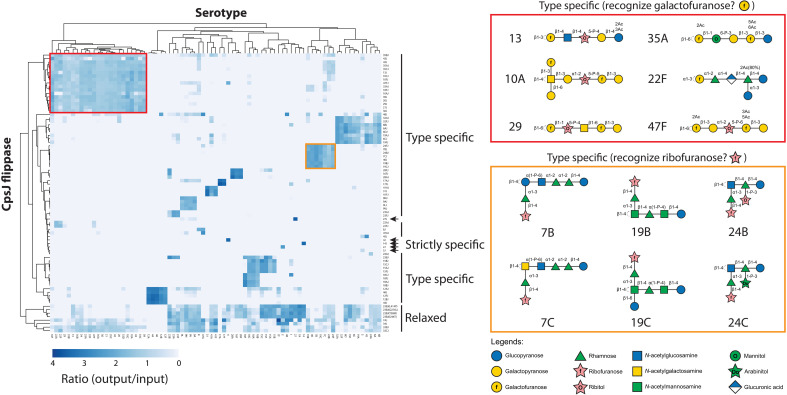
Capsule transporters can be classified into three groups. Hierarchical clustering done by Clustvis (*49*) on the heatmap shown in Fig. 2E revealed three classes of *cpsJ* alleles. Type-specific CpsJ transporters are specific to certain CPS precursors, likely recognizing some structural features like galactofuranose and ribofuranose residues at the nonreducing ends (red and orange boxes on the right). Strictly specific CpsJ flippases (as indicated by the arrows) exclusively transport their cognate precursors. Last, CpsJ flippases with relaxed specificity (“relaxed”), such as Cps23BJ** and Cps7AJ, can transport many types of cargo. The reducing ends are on the right, and the curved arrows on the left indicate the polymerization sites.

Next, we introduced the barcoded *cpsJ* constructs into the rest of the capsule-switch strains (*29*). On average, we recovered ~500,000 transformants in the input library. The output library contains ~1 million transformants, indicating that the capsule-switch mutants remained transformable. Most of the barcoded *cpsJ* constructs were functional because they were capable of complementing their cognate flippases (Fig. 2E). However, similar to *cps33DJ*, ectopically expressed *cps1J* and *cps7FJ* were unable to complement their cognate flippases. Possibly, the expression levels of these flippases were too low. We also encountered difficulty amplifying the flanking regions of *cps25AJ*, *cps25FJ*, and *cps38J*. Therefore, these *cpsJ* alleles were excluded. A total of 80 *cpsJ* alleles were tested in 79 capsule-switch strains. Thus, 6320 substrate-flippase combinations were examined (Fig. 2E). After hierarchical clustering, we identified three categories of *cpsJ* flippases: “Strictly specific” flippases can only flip their cognate cargo. “Type-specific” flippases appear to recognize structural components of the substrate and have limited ability to substitute each other. Last, several flippases have relaxed specificity (e.g., *cps7AJ* and *cps34J*), capable of transporting many noncognate cargos (Fig. 3).

### Cps23BJ**, Cps7AJ, Cps33BJ, Cps33CJ, and Cps34J could transport many capsule precursors

As expected, the four cps23BJ** alleles we isolated could transport additional precursors (≈40% of all precursors tested) compared to *cps23BJ*^+^ (≈10% of all precursors tested). This result confirmed that the additional mutations in cps23BJ** significantly reduced substrate selectivity (Fig. 3). Yet, cps23BJ** could not transport teichoic acid precursors (*30*). Several *cpsJ* alleles (*cps7AJ*, *cps33BJ*, *cps33CJ*, and *cps34J*) have more relaxed specificity than cps23BJ** (fig. S2). Although not genetically engineered to reduce their specificity, they could transport up to ≈50% of the noncognate precursors tested. However, despite their ability to transport many substrates, they also could not transport teichoic acid precursors (table S6).

### Type-specific flippases transport structurally similar precursors

We demonstrate that the primary sequences of *cpsJ* were associated with the CPS precursors by which they transport (*21*). As expected, flippases in the same serogroup are more likely to complement each other (Fig. 3). We search for common glycan motifs in the substrates shared by the type-specific flippases to identify sugar residues recognized by the transporter. For example, flippases in serogroups 10 and 35 seem to recognize CPSs that contain galactofuranose at the nonreducing ends. Similarly, some serogroups 7, 19, and 24 flippases may detect ribofuranose of the branch chain because this residue is the only constituent shared by these serotypes (Fig. 3 and fig. S2). As capsule synthesis starts from the reducing end, recognizing sugar residues at the nonreducing end might ensure only the completed lipid-linked precursor is translocated across the cell membrane.

### Transport of CPS precursor is likely size dependent

Substrate binding to the central binding cavity is thought to trigger a conformational change that leads to the transition of the flippase to the outward-facing state. This conformation subsequently releases the substrate (*26*, *31*). If the CPS substrates are too large, they may be unable to fit into the substrate binding pocket due to steric hindrance. The serotype 17A precursor is the largest among pneumococcal CPS precursors, with eight sugar residues in the repeating unit. Consistently, the serotype 17A cargo could only be transported by Cps17AJ and its close homolog Cps17FJ (fig. S3).

**Fig. 4.**
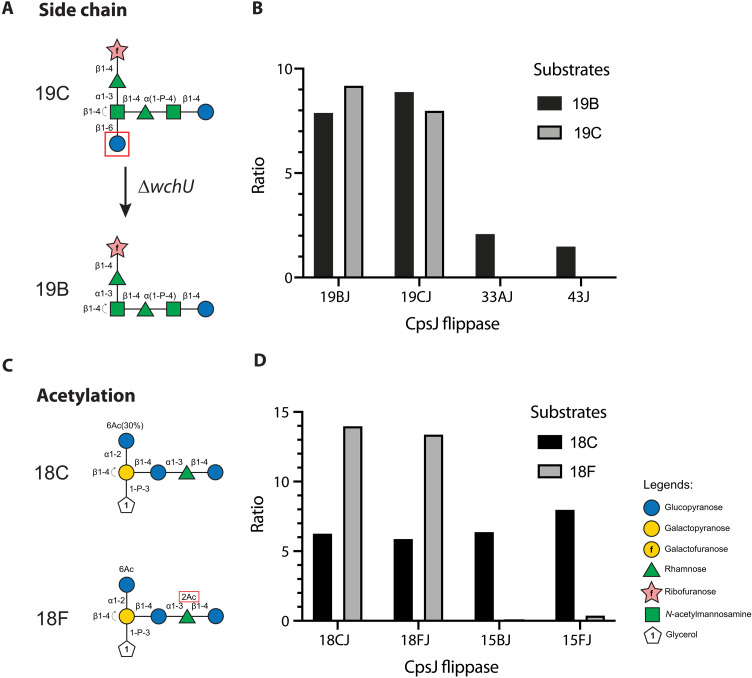
Substrate features that affect transport by CpsJ. (**A**) The additional glucose residue in serotype 19C may impede substrate translocation by Cps33AJ and Cps43J. (**B**) The lack of a functional *wchU* (i.e., 19B) enabled complementation of *cps19CJ* by *cps33AJ* and *cps43J*. Shown are the results of Bar-seq. An increase in the ratios of the read counts in the libraries after and before *cpsJ* deletions suggests transportation of the indicated substrates by the ectopic *cpsJ* allele. The findings were validated by reconstructing the strains. Briefly, strains NUS4071 [CPS19B // P-*cps33AJ*], NUS4072 [CPS19B // P-*cps43J*], NUS4074 [CPS19C // P-*cps33AJ*], NUS4075 [CPS19C // P-*cps43J*], NUS4188 [CPS19C ∆*wchU* // P-*cps33AJ*], and NUS4189 [CPS19C ∆*wchU* P-*cps43J*] were grown in BHI broth at 37°C in 5% CO_2_ until they reached the early exponential phase. Cultures were transformed with the ∆*cps19CJ* or ∆*cps19BJ* cassette as indicated. Transformants were plated on blood agar plates and incubated overnight at 37°C in 5% CO_2_ before imaging. See also Table 1. Experiments were done twice with similar results. (**C**) Acetylation impedes translocation of the serotype 18F substrate by Cps15BJ. (**D**) Cps15BJ could complement Cps18CJ but not Cps18FJ. The findings were validated by reconstructing the strains. Briefly, strains NUS4203 [CPS18C // P-*cps15BJ*], NUS4204 [CPS18C // P-*cps15FJ*], NUS4205 [CPS18F // P-*cps15BJ*], and NUS4206 [CPS18F // P-*cps15FJ*] were grown in BHI broth at 37°C in 5% CO_2_ until they reached the early exponential phase. Cultures were transformed with the ∆*cps18FJ* or ∆*cps18CJ* cassette as indicated. Transformants were plated on blood agar plates and incubated overnight at 37°C in 5% CO_2_ before imaging. See also Table 2. Experiments were done twice with similar results. See also fig. S5.

Smaller CPS precursors are more likely to be transported by noncognate flippases. For example, the serotype 19B and 19C precursors are identical, except the latter has an additional glucose residue at the nonreducing end of the branch chain (Fig. 4A). Compared to serotype 19C, serotype 19B precursors could be transported by additional flippases like Cps20J, Cps33AJ, and Cps43J (Fig. 4B, fig. S4, and table S7). To validate this result, we tested whether Cps33AJ and Cps43J could complement Cps19BJ and Cps19CJ. Consistently, Cps33AJ and Cps43J could not transport serotype 19C cargo, likely because of the additional glucose residue (Table 1). To examine whether this glucose residue prevents cross-complementation, we deleted *wchU* in serotype 19C, which is the glycosyltransferase responsible for installing the branch chain glucose residue (Fig. 4A and Table 1). *wchU* is dispensable for growth, likely because *cps19CJ* could transport the incomplete precursor (*30*). When deleted, it converted serotype 19C into serotype 19B (Fig. 4A). Similarly, introducing *wchU* into a serotype 19B strain converted it back to serotype 19C (*30*). ∆*wchU* allowed *cps33AJ* and *cps43J* to complement *cps19CJ* (Table 1), suggesting that the extra glucose residue of the serotype 19C CPS prevents *cps33AJ* and *cps43J* from complementing *cps19CJ*, likely because the substrate cannot fit into the central aqueous cavity of the flippase.

**Table 1.** The addition of sugar residue in the serotype 19C capsule affects cross-complementation. –, not tested.

Genotype of recipient cells	Transformants obtained when the indicated amplicon was introduced (CFU)	
	∆*cps19BJ::*P*-erm*	∆*cps19CJ::*P*-erm*
*rpsL1* CPS19B	6^*^	–
*rpsL1* CPS19B P-*kan*-*cps19BJ*	>300	–
*rpsL1* CPS19B P-*kan*-*cps33AJ*	>300	–
*rpsL1* CPS19B P-*kan*-*cps43J*	>300	–
*rpsL1* CPS19C	–	7^*^
*rpsL1* CPS19C P-*kan*-*cps19CJ*	–	>300
*rpsL1* CPS19C P-*kan*-*cps33AJ*	–	10^*^
*rpsL1* CPS19C P-*kan*-*cps33AJ* ∆*wchU*	–	>300
*rpsL1* CPS19C P-*kan*-*cps43J*	–	8^*^
*rpsL1* CPS19C P-*kan*-*cps43J* ∆*wchU*	–	>300

*These clones are likely suppressors because the cells were unencapsulated.

### Acetylation affects substrate translocation

The precursor of serotype 18F is similar to that of 18C, except it is acetylated at the rhamnose residue (Fig. 4C) (*10*). While many transporters could flip both precursors, only the unacetylated serotype 18C cargo could be transported by flippases in serogroups 10 and 15 as well as serotypes 29 and 46 (Fig. 4D and fig. S4). We then examined whether Cps15BJ and Cps15FJ could transport serotypes 18C and 18F precursors (Fig. 4C and Table 2). Cps15BJ could only substitute the function of Cps18CJ but not Cps18FJ, indicating that acetylation affects the transport of the capsule substrates (Table 2).

**Table 2.** Acetylation of the serotype 18F capsule affects cross-complementation. –, not tested.

Genotype of recipient cells	Transformants obtained when the indicated amplicon was introduced (CFU)	
	∆*cps18CJ::*P*-erm*	∆*cps18FJ::*P*-erm*
*rpsL1* CPS18C P-*kan*-*cps18CJ*	>300	–
*rpsL1* CPS18C P-*kan*-*cps15BJ*	>300	–
*rpsL1* CPS18C P-*kan*-*cps15FJ*	>300	–
*rpsL1* CPS18F P-*kan*-*cps18FJ*	–	>300
*rpsL1* CPS18F P-*kan*-*cps15BJ*	–	3^*^
*rpsL1* CPS18F P-*kan*-*cps15FJ*	–	>300

*These clones are likely suppressors because the cells were unencapsulated.

Similarly, acetylation may affect capsule precursor transport by Cps23BJ**. The precursor of serotype 33A, but not 33F, is acetylated (*10*). The acetylation site is at the galactofuranose residue near the reducing end (fig. S5). We noticed that cells expressing Cps23BJ** formed smaller colonies if they also expressed serotype 33A, but not 33F, capsule (fig. S5), suggesting that acetylation somehow interferes with precursor transport by Cps23BJ**. The reduction of colony sizes was not due to the loss of the capsule, as judged by immunostaining of the serogroup 33 capsule (fig. S5). The phenotype was similar in the merodiploid strain harboring Cps33AJ and Cps23BJ** (fig. S5). Thus, this result cannot be explained by the inability of Cps23BJ** to flip the serotype 33A precursor. One possible explanation is that Cps23BJ** may flip incomplete but acetylated precursors of serotype 33A. If so, it may indicate that acetylation occurs in the cytoplasm, contrary to the earlier prediction (*12*). We attempted to test this hypothesis by inactivating the acetyltransferase WcjE. Yet, we could not obtain viable ∆*wcjE* mutants, likely because this gene is essential for growth.

### The fitness costs of reducing the specificity of capsule flippases

**Fig. 5.**
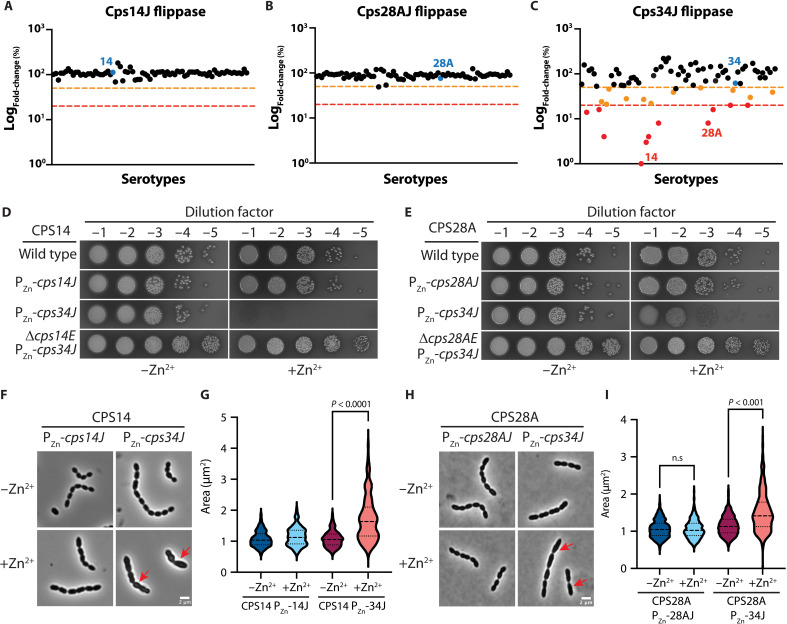
Expression of flippases with relaxed specificity causes growth defects. (**A** to **C**) Cells expressing Cps34J but not Cps14J or Cps28J exhibited growth defects. The cognate flippases are highlighted in blue. Dots below the orange line indicate a >2-fold decrease in abundance, and dots below the red line have a >5-fold decrease. (**D**) Cps34J is toxic in the serotype 14 background. Strains NUS0403, NUS4643, NUS4644, and NUS5047 were grown in BHI broth at 37°C in 5% CO_2_ until the OD_600_ was between 0.2 and 0.4. Cultures were normalized based on their optical density, serially diluted, and spotted on blood agar without (left) or with (right) added ZnCl_2_ and MnCl_2_. (**E**) Strains NUS0671, NUS4207, NUS4285, and NUS5048 were grown in BHI and spotted on blood agar plates as described in (D). (**F**) Strains NUS4643 and NUS4644 were grown to the early log phase and normalized to an OD_600_ of 0.1. Growth was continued for an hour with or without the supplement of ZnCl_2_ and MnCl_2_ before imaging by phase contrast microscopy. Red arrows indicate defective cells. Scale bar, 2 μm. (**G** and **I**) The areas of the cells were quantified by MicrobeJ [*n* = 889 for (G) and 1689 for (I)]. *P* values were computed by Mann-Whitney *U* tests. n.s., not significant. (**H**) Strains NUS4207 and NUS4285 were grown and imaged as described in (F). Shown in (D), (E), (F), and (H) are representative images from three biological replicates. See also figs. S6 to S15.

The flippase is thought to be a molecular checkpoint to retain incomplete CPS precursors in the cytoplasm (*21*). Strains harboring a flippase with relaxed specificity may lead to growth defects because incomplete precursors may be accidentally transported to the outer leaflet of the cell membrane (*21*). Because there is no known mechanism to flip these precursors back to the cytoplasm, they become dead-end metabolites that sequester Und-P and disrupt PG synthesis. When we examined the number of reads in the input libraries, we detected fewer barcode reads representing flippases with relaxed specificity (i.e., Cps7AJ, Cps33BJ, Cps33CJ, Cps34J, and Cps23BJ**) in some serotypes (Fig. 3 and fig. S2). This observation suggests that these flippase alleles are toxic. To understand the mechanism, we identified serotypes with a more than 50% decrease in the number of reads compared to the mean (figs. S6 and S7). We chose Cps34J as a prototype to study flippase toxicity by introducing the P-*kan*-*cps34J* cassette into isogenic capsule-switch mutants of serotypes 14 and 28A (Fig. 5, A to C). Alternatively, the cassette was introduced into isogenic strains where the initiating phosphoglycosyl transferase *cpsE* was inactivated. ∆*cpsE* alleviates the lethality caused by lesions in the capsule synthesis pathway (*20*), and thus, the ∆*cpsE* strain serves as a positive control. Consistent with Cps34J being toxic in strains producing serotype 14 and 28A capsules, the P-*kan*-*cps34J* cassette could only be introduced in strains harboring ∆*cps14E* or ∆*cps28AE* but not their isogenic parents (Fig. 5, D and E, and table S8). We showed that the serotype 14 and 28A strains remained transformable because an unrelated cassette (i.e., P-*kan*-*cps28AJ*) could be readily introduced. Consistent with the Bar-seq results, *cps34J* led to a more pronounced growth defect in serotype 14 compared to serotype 28A (Fig. 5, C to E). Why Cps34J is toxic only in these serotypes remains enigmatic, as we could not detect correlations between the CpsJ sequences, the structure of the substrates, and the phenotype (fig. S8). Together, we provide experimental evidence that expressing flippases with relaxed specificity may have a fitness cost.

To study the phenotype associated with the expression of Cps34J, we placed *cps34J* downstream of an inducible promoter at the ectopic locus [∆*bgaA*::P_Zn_-*cps34J*] in the serotype 14 and 28A background (Fig. 5, D and E). When Zn^2+^ was added, *cps34J* expression was induced, and these strains formed smaller colonies and had a slower growth rate than their parent strains in liquid culture (Fig. 5, D and E, and fig. S9). Consistent with PG synthesis being inhibited, we detected cell shape defects when *cps34J* was expressed (Fig. 5, F to I). These defects are dependent on *cps14E* or *cps28AE* expression (fig. S9) (NUS4911[CPS14 ∆*cps14E* // P_Zn_-*cps14E* // P-*kan*-*cps34J*] or NUS4600 [CPS28A ∆*cps28AE* // P_Zn_-*cps28AE* // P-*kan*-*cps34J*]), indicating that *cps34J* toxicity requires capsule synthesis. Next, we measured the relative amount of CpsJ by fusing *cpsJ* at the native or ectopic locus with a C-terminal FLAG tag (DYKDDDDK), followed by immunoblotting. Overall, the expression level of *cpsJ-*FLAG at the ectopic locus is higher than the native locus (fig. S9). The expression of *cpsE* has little effect on CpsJ levels (fig. S9). When expressed at the ectopic locus, Cps34J-FLAG levels were comparable in the serotype 14, 28A, and 34 capsule-switch mutants with less than twofold differences (fig. S9). Thus, the toxicity of *cps34J* cannot be explained by the pleiotropic effect caused by overexpression of an essential membrane protein. We propose that flippases with relaxed specificity transport incomplete precursors across the cell membrane, thereby sequestering Und-P and inhibiting PG synthesis.

### A cassette containing multiple flippases allows the translocation of noncognate CPS substrates

We showed that expressing Cps23BJ** renders several CPS glycosyltransferases dispensable (*30*), suggesting that reducing the specificity of the flippase could widen the bottleneck of genetic glycoengineering. To this end, we constructed the “*TRIO*” cassette by expressing Cps7AJ, Cps12AJ, and Cps19AJ in a single construct. These flippases were selected based on the results of the cross-complementation experiment, which predicted that this combination could transport many types of CPS precursors (Fig. 3). We demonstrated that the *TRIO* cassette is functional and supports the trafficking of cognate substrates (table S9 and fig. S10). In addition, we confirmed that the *TRIO* cassette, but not Cps23BJ**, could replace Cps17FJ and Cps33BJ (table S9 and fig. S11). Next, we tested whether the *TRIO* cassette allows deletions of glycosyltransferases that remained essential in the presence of Cps23BJ** (table S10) (*30*). Among the 13 GTs tested, 11 were still required for growth regardless of whether the cells harboring cps23BJ** or the *TRIO* cassette. Two of the GTs that were previously annotated essential (WcwH in serotype 7A and WcrK in serotype 35C) are dispensable for growth (fig. S11) (*30*). Our results indicate that while the *TRIO* cassette could presumably flip more types of substrates, it is insufficient to relax the constraint of genetic glycoengineering by trafficking incomplete precursors.

### Machine learning unveils additional residues that may govern specificity

Our findings also provide a training dataset for machine learning approaches. For simplicity, we binarized our results by defining complementation as no reduction in the barcode abundance corresponding to the specific *cpsJ* allele (i.e., output/input ratios greater than or equal to 1). Root mean square deviations (RMSDs) of the AlphaFold prediction models of CpsJ weakly correlate with the complementation outcome (Pearson’s correlation = −0.25, *P* < 2.2 × 10^−16^). We used a supervised machine learning approach to determine amino acid residues important for specificity. A random forest model was trained on experimentally determined complementation outcomes of each serotype and flippase pair. It revealed two positions (residues 254 and 339 in Cps23BJ) that may determine specificity. These residues are in the α helices near the central aqueous cavity (fig. S12). When position 254 or its equivalent residue is isoleucine, leucine, or proline, CpsJ seems more specific than having a valine residue at that position (fig. S13). Consistent with this position being important for substrate selection, changing the proline residue at position 254 to serine further reduces the specificity of Cps23BJ (*21*) (Fig. 1E).

Similarly, threonine at position 339 or the equivalent residue is associated with relaxed specificities (fig. S13). To test whether this position determines specificity, we changed the equivalent threonine residue in Cps34J to phenylalanine [i.e., Cps34J(T333F)]. Unmutagenized Cps34J could complement Cps41AJ and Cps47AJ (Fig. 3). To investigate whether Cps34J(T333F) could also complement these flippases, we introduced *cps34J*(T333F) into the capsule-switched mutants of serotype 41A and 47A. Next, we deleted *cps41AJ* and *cps47AJ* to test whether we could obtain viable transformants. Fewer colonies were obtained when *cps47AJ* was deleted in serotype 47A strains harboring *cps34J*(T333F) but not *cps34J*^+^ (table S11). Moreover, deleting *cps41AJ* in the capsule-switch mutant of serotype 41A that expressed *cps34J*(T333F) but not *cps34J*^+^ resulted in transformants that formed smaller colonies. Cps34J(T333F) is functional because it could complement Cps34J in the serotype 34 background (table S11). These results confirmed that position 339 is involved in determining specificity. Introducing an amino acid residue with a bulky group like phenylalanine at this location likely hinders the noncognate substrate from being transported by the flippase.

## DISCUSSION

Here, we examined the specificity of MOP transporters by identifying cross-complementation events in ~6000 combinations of capsule transporters and substrates. Our results indicate that the specificity of *cpsJ* varies significantly, ranging from strictly specific flippases to variants capable of flipping many noncognate substrates. Substrate selectivity could be considerably widened by introducing only two amino acid changes (e.g., Cps23BJ**), some of which might have occurred naturally (e.g., Cps7AJ and Cps34J). Relaxing the specificity of CpsJ enables changes in the capsule structure (*30*). Nevertheless, it may cause growth defects, depending on the production of certain capsule types. This leads to a model that nonspecific flippases may inadvertently transport incomplete precursors across the cell membrane. Moreover, we performed structural modeling and machine learning experiments to predict the amino acid residues that govern specificity. Some of these findings were confirmed experimentally. Overall, this study provides insights into the mechanism by which MOP flippases select and transport their cargo.

The sizes of the precursors play an essential role in substrate selection. For example, acetylation and extra branch chains could block substrate translocation by CpsJ. Structural modeling and computational docking experiments predicted that the glucose residue on the side chain of the serotype 19C precursor likely clashes with the transmembrane helix of Cps33AJ and Cps43J but not Cps19BJ (fig. S14). Yet, docking the serotype 18C precursor to Cps15BJ could not explain why acetylation would block Cps15BJ from flipping the substrate. Occasionally, exceptions exist that argue against the notion that flippases transporting larger cargo are less specific. For example, the first three sugar residues of serotype 19B and 19C CPS are identical to those of serotype 19A and 19F (*21*). They have a common precursor [Rha-ManNAc-Glu-PP-Und] in their pathways. While serotype 19B and 19C precursors have additional sugar residues, Cps19BJ and Cps19CJ were unable to complement Cps19AJ and Cps19FJ (Fig. 3). In addition, we found that sugar residues at the nonreducing ends may be recognized by the capsule flippase (Fig. 3). Interactions between these residues and the substrate binding pocket may trigger a conformational change of the flippase from the inward-facing to the outward-facing state, thereby releasing the cargo to the other side of the cell membrane.

In addition to amino changes around the extracellular gate (e.g., T38M and I246T in Cps23BJ) (*21*, *27*, *28*), we found that mutations at the intracellular gate could also relax specificity (e.g., Q276L and G347V in Cps23BJ) (Fig. 1E and fig. S1). The residues involved may stabilize the two loops of the helix bundles. When mutated, the inward-open state may be destabilized (*32*), leading to a spontaneous transition to the outward-open state that releases the noncognate substrates. It is tempting to speculate that these amino acid changes can occur naturally to increase the flexibility of the capsule pathway because the inability to flip the altered CPS substrate is likely lethal. We found that Cps7AJ and Cps33BJ could flip a structurally diverse set of cargo like Cps23BJ**, except the latter were isolated after two rounds of genetic selection (Fig. 3). Cps7AJ, Cps33BJ, Cps33CJ, and Cps34J could not flip lipid II, suggesting that they retain some levels of specificity. We speculate that capsule flippases avoid lipid II as a protective mechanism because they may also flip lipid I.

It remains to be determined why certain flippases can accommodate a broader range of substrates. As discussed above, flippase variants with relaxed specificity may be evolutionary intermediates for diversifying the CPS composition. In addition to the postulation that flipping incomplete precursors sequesters Und-P (fig. S15), the unfinished precursors flipped may inhibit the polymerase and the ligase because they are structural analogs of their cognate substrates (fig. S15). Then, why does pneumococcus keep these flippases? One possibility is that these flippases confer a competitive advantage in the host because the cells can adjust their CPS in response to anti-capsular antibodies. Besides reducing surface antigenicity, changing sugar residues and glycosidic linkages may produce better capsules for evading immune cells like Kupffer cells in the liver (*33*). These cells are known to capture pathogens using specific glycan receptors (*33*). Once the new capsule is installed, the fidelity of the pathway can be restored by increasing the stringency of the flippase, avoiding the fitness costs associated with the expression of nonspecific flippases. Future work is under way to elucidate the phylogenetic relationships of capsule types to confirm this postulation.

MOP transporters are not the only class of membrane proteins that can flip a variety of substrates. The ABC transporter Wzk flips diverse lipid-linked precursors such as O-antigens and lipid II (*34*, *35*). Likewise, the Wzm-Wzt exporter has no apparent specificity for the glycan portion of the cargo unless it contains C-terminal carbohydrate-binding modules that recognize the terminal sugar (*36*). Cells expressing these flippases usually have no noticeable growth defects (*5*, *37*). While we found a few dozen scenarios contradicting this observation, flippases with relaxed specificity seem to cause mild phenotypes, if any. These results suggest that there are mechanisms to remove incomplete precursors on the wrong side of the membrane. For example, the tyrosine kinase system CapAB in *Staphylococcus aureus* releases Und-P by cleaving the glycan portion of the lipid-linked precursors in vitro (*38*). It may facilitate the removal of incomplete precursors that are flipped. Another possible mechanism is to control the lateral diffusion of the incomplete precursors by metabolic channeling (*39*). Consistent with this speculation, CPS enzymes in serotype 2 are organized into a multienzyme complex (*40*). Therefore, glycosyltransferases may outcompete the flippase in terms of access to the incomplete precursors, which may explain why the nonspecific flippases are not toxic in many serotypes. Last, the incomplete precursors may also flip back into the cytoplasm. These possible fail-safe mechanisms are to be examined in the future. In addition, flippase variants with a further reduction in specificity will be isolated.

## MATERIALS AND METHODS

### Media and culturing conditions

The strains used in this study are listed in table S12. Cells were grown in brain heart infusion (BHI) broth (Thermo Fisher Scientific) or on tryptic soy agar plates supplemented with 5% (vol/vol) sheep blood (blood plates) (Biomed Diagnostics) at 37°C in 5% CO_2_. Antibiotics purchased from Sigma-Aldrich were used at a final concentration of 0.3 mg/ml for erythromycin (Erm), 300 mg/ml for streptomycin, and 250 mg/ml for kanamycin (Kan). When indicated, ZnCl_2_ and MnCl_2_ were added to the liquid culture at final concentrations of 500 and 50 μM, respectively. Mn^2+^ was added to the medium to alleviate Zn^2+^ toxicity (*41*, *42*).

### Strain construction

Primers for generating PCR amplicons are listed in table S13. In general, PCR fragments were synthesized using high-fidelity Phusion DNA polymerase (NEB, M0530S) according to the manufacturer’s instructions. PCR products were evaluated by agarose gel electrophoresis and purified using the QIAquick PCR purification kit (Qiagen, 28106). Pneumococcal cultures were grown to an OD_600_ (optical density at 600 nm) of 0.1 to 0.3, normalized to an OD_600_ of 0.03, before adding competence stimulating peptide to induce competence (*43*). After induction, pneumococcal cells were transformed with cassettes assembled by Gibson assembly (*44*). Transformants were selected on blood plates supplemented with the indicated antibiotics. For the construction of barcoded *cpsJ* strains, IU1781 [D39W *rpsL*] was transformed with cassettes harboring a Kan-resistant gene driven by a constitutive promoter (P-*kan*), the corresponding *cpsJ* allele, and a unique 9-nucleotide barcode using the primers and templates listed in table S13. Transformants were selected for Kan resistance. All constructed strains were validated by PCR using either GoTaq DNA polymerase (Promega, M712) or 2X PowerPol polymerase (ABclonal, RK20718), followed by Sanger sequencing.

### Isolation of cps23BJ**

The P-*kan-cps23BJ*(P254S) cassette (*21*) was PCR mutagenized using the oligonucleotides listed in table S13 as described before (*21*). Briefly, the amplicon was synthesized with 30 cycles of amplification using error-prone GoTaq DNA polymerase. The same amplicon was amplified using proofreading Phusion DNA polymerase as the negative control. PCR products were purified using the Qiagen PCR purification kit. Strain NUS1992 [*rpsL1* ∆*ytgP*::P-*erm* // ∆CEP::P_Zn_-*ytgP*] was transformed with the PCR amplicons and selected for Kan resistance in the presence of ZnCl_2_/MnCl_2_. Transformants were pooled, serially diluted, and selected for survivors (cps23BJ**) without ZnCl_2_/MnCl_2_ supplementation. Under this condition, surviving cells likely harbor mutations in the *cps23BJ* alleles that substitute *ytgP*. These transformants were then pooled. DNA was extracted from the pooled transformants and was used to transform the parent strain NUS1992. A significant increase (~100-fold) in plating efficiency was observed compared to the cells transformed with the *cps23BJ*(P254S) amplicon without PCR mutagenesis. In total, 64 *cps23BJ* alleles were sequenced, and the causative mutations were validated by transforming the alleles back into the parental strain NUS1992 or by site-directed mutagenesis when there was more than one mutation in the *cps23BJ*(P254S) allele (*21*).

### Library construction and Bar-seq

Amplicons of the barcoded *cpsJ* alleles [CEP::P-*kan*-*cpsJ*-barcode] were amplified by high-fidelity Phusion DNA polymerase using the oligonucleotides listed in table S13 and purified. The concentration and yield of the barcoded *cpsJ* amplicons were normalized by their band intensities after agarose gel electrophoresis. The barcoded *cpsJ* amplicons were then pooled and introduced into the isogenic capsule-switched strains previously constructed (*29*). These capsule switch mutants were constructed by replacing the *cps* genes between *dexB* and *aliA* of D39W with the *cps* operon of the other serotypes. Changing the *cps* locus is sufficient for displaying a different capsule type in the D39W genetic background. Because these strains are isogenic, the pool of *cpsJ* constructs could be inserted into the same genetic locus [i.e., the CEP site (*45*)], and the transformants were selected for Kan resistance. Transformants on the plate were collected by adding BHI broth and scraping the plates with a spreader. The cell suspension was pooled and stored at −80°C or directly used for subsequent experiments. The pooled transformants were normalized to an OD_600_ of 0.05 and incubated for 2 hours at 37°C in 5% CO_2_. After the induction of competence, cultures were transformed with 10 μl of PCR amplicon containing an Erm-resistant cassette that is flanked by the respective *cps* operon (∆*cpsJ*::P-*erm*) to delete the corresponding native *cpsJ* in each serotype. Cells were incubated for 1.5 hours at 37°C in 5% CO_2_ and plated onto blood plates supplemented with Erm. Because *cpsJ* is required for growth, the parent capsule-switched strains without the ectopic *cpsJ* cassette were transformed with the ∆*cpsJ*::P-*erm* cassette as a negative control to estimate the number of spontaneous suppressors. Survivors on the plates were collected and pooled. Genomic DNA was purified using the DNeasy blood and tissue kit (Qiagen, 69606). The DNA barcodes were amplified by the KAPA Library Amplification Kit (Roche, KK2611) using the primers in table S13. These primers contain another barcode for multiplexing, as described previously (*29*). Up to seven libraries were pooled and sequenced through the Novaseq PE250 (2 million reads per sample) or PE150 platform (6 million reads per sample). Data were demultiplexed with CLC genome workbench (Qiagen) and exported to Microsoft Excel for further processing. The number of barcode reads was normalized to reads per one million reads (RPM). To determine the relative abundance of the noncognate *cpsJ* alleles, the number of barcode reads (RPM) from the output library was divided by the input library (RPM).

### Transformation efficiency tests for evaluating the capsule flippase function

Pneumococcal cultures were grown to an OD_600_ of 0.1 to 0.3 and normalized to an OD_600_ of 0.03 before competence-stimulating peptide was added to induce competence. After inducing competence, pneumococcal cultures were transformed with PCR fragments harboring antibiotic markers flanked by the indicated regions to replace *cpsJ* by homologous recombination. Cultures were incubated for 1.5 hours at 37°C in 5% CO_2,_ and the transformants were selected on plates with the appropriate antibiotics.

### Bioinformatics analyses

CpsJ sequences were retrieved from the National Center for Biotechnology Information website and converted to protein sequences via A plasmid Editor (ApE) (*46*). Structural models of CpsJ were generated by AlphaFold2 (*47*) and visualized with PyMOL (http://pymol.org). Conserved residues of the CpsJ flippases were determined by Consurf (*48*). The RMSD value between the pair of flippase structures was generated via PyMOL. Structures of the CPS repeating units were downloaded from the Carbohydrate Structure database (http://csdb.glycoscience.ru/) and converted into SMILES file for visualization with DataWarrior (https://openmolecules.org/datawarrior/). The Tanimoto coefficient that compares two CPS structures was generated by ChemMine Tools (https://chemminetools.ucr.edu/). Phylogenetic relationship and hierarchical clustering are plotted using Clustvis (*49*). For the settings, no scaling is applied to rows. Rows (representing serotypes) and columns (representing CpsJ flippases) are clustered using “Manhattan distance” and “complete linkage” options. Docking of CPS substrates with the CpsJ structural model was performed using Chai-1 (https://lab.chaidiscovery.com).

CpsJ amino acid primary sequences were aligned using the MUSCLE alignment algorithm using default parameters in MEGA (*50*). Data were handled in RStudio (v 2023.09.1+494), and the random forest models were run using SciKit-Learn (https://scikit-learn.org). All relevant files and codes are deposited on GitHub (https://github.com/ally-hawk/flippase.chua2024).

### Measurement of growth and microscopy

Overnight cultures of the indicated strains were grown in BHI. When the OD_600_ reached 0.2 to 0.4, cultures were normalized to an OD_600_ of 0.2 and diluted to an OD_600_ of 0.01 in BHI with or without ZnCl_2_/MnCl_2_. The diluted cultures were distributed into a 96-well plate at a final volume of 200 μl. Growth was monitored at 37°C for 12 hours using a Tecan microplate reader. OD_600_ readings were taken every 10 min immediately after a short pulse of 30 s of shaking.

To visualize the cells after induction of noncognate CpsJ expression, overnight cultures were diluted to an OD_600_ of 0.05 in BHI supplemented with or without ZnCl_2_/MnCl_2_ and grew for 2 hours at 37°C. The cells were harvested by centrifugation at 8000*g* for 1 min at room temperature. The supernatant was removed, and the pellets were resuspended in 100 μl of BHI. Cells were mounted on a glass slide with an agar pad and imaged with an IX81 phase-contrast microscope (Olympus). Micrographs were analyzed and quantified using MicrobeJ, as described previously (*51*).

### Immunofluorescence microscopy

Cultures were grown to an OD_600_ between 0.2 and 0.4, normalized to an OD_600_ of 0.2, and heat inactivated at 56°C for 45 min. Cells were harvested by centrifugation at 20,000*g* for 2 min at room temperature, washed with 1× phosphate-buffered saline (PBS), and resuspended in 100 μl of cross-absorbed anti-CPS antisera of serotype 33 (Statens Serum Institut) at a dilution of 1:300. The mixture was incubated on ice for 5 min. Cells were collected by centrifugation, washed twice with 1× PBS, and resuspended in 100 μl of 1× PBS. Antirabbit Alexa Fluor 488 antibodies (Thermo Fisher A11034) were added at a 1:100 dilution to the cells and incubated on ice for 5 min. The labeled cells were washed once with 1× PBS, resuspended in 3 μl of SlowFade mounting media (S36936 Invitrogen), and visualized with an IX81 microscope (Olympus).

## Supplementary Materials

### The PDF file includes:

Figs. S1 to S15 Tables S1 to S13 Legend for data S1 References
