# Supplementary material for: Massively parallel barcode sequencing revealed the interchangeability of capsule transporters in Streptococcus pneumoniae
Source: Sci Adv. 2025 Jan 24;11(4):eadr0162. doi: 10.1126/sciadv.adr0162 (PMC11759038; doi:10.1126/sciadv.adr0162)
Supplement: Supplementary file 1 — Figs. S1 to S15 Tables S1 to S13 Legend for data S1 References [file sciadv.adr0162_sm.pdf]

Science Advances
AAAS

Supplementary Materials for

Massively parallel barcode sequencing revealed the interchangeability of capsule transporters in *Streptococcus pneumoniae*

Wan-Zhen Chua *et al.*

Corresponding author: Lok-To Sham, lsham@nus.edu.sg

Sci. Adv. **11**, eadr0162 (2025)
DOI: 10.1126/sciadv.adr0162

The PDF file includes:

Figs. S1 to S15
Tables S1 to S13
Legend for data S1
References

Other Supplementary Material for this manuscript includes the following:

Data S1

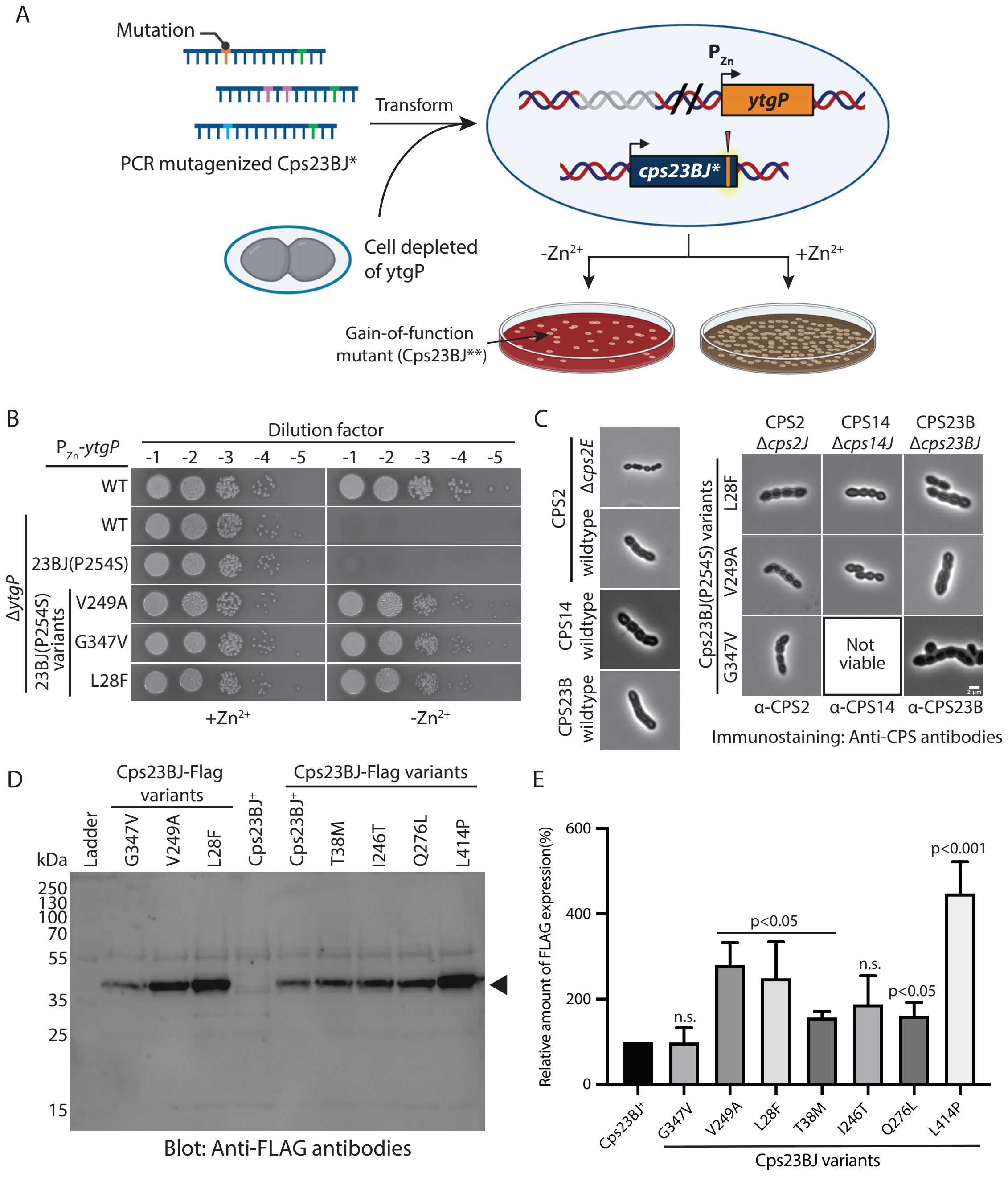

Fig. S1. Cps23BJ variants support lipid II transport. (A)** A schematic for isolating gain-of-function Cps23BJ** mutants that transport lipid II. The *cps23BJ*(*P254S*) allele was PCR mutagenized. The mutagenized *cps23BJ*(*P254S*) allele was introduced into the strain depleted of the cell wall flippase (*ytgP*). Gain-of-function mutants were then selected in the absence of the inducer and identified by Sanger sequencing. (**B**) Strains NUS1992 [ΔCEP::P_{Zn}-*ytgP* Δ*ytgP*::P-*erm*], NUS2247 [ΔCEP::P_{Zn}-*ytgP*], NUS5030 [P-*kan-cps23BJ*(P254S, V249A)], NUS5065 [P-*kan-cps23BJ*(P254S)], NUS5140 [P-*kan-cps23BJ*(P254S, L28F)] and NUS5049 [P-*kan-*

cps23BJ(P254S, G347V)] were grown in BHI broth at 37°C in 5% CO_2 until they reached the early exponential phase. Cultures were serially diluted and spotted on blood agar plates with or without supplements of Zn^{2+} and Mn^{2+}. Plates were imaged after incubation at 37°C in 5% CO_2 overnight. (**C**) Cps23BJ(P254S L28F), Cps23BJ(P254S V249A), but not Cps23BJ(P254S G347V) support transport of serotype 2, 14, and 23B cargo. Shown are phase-contrast micrographs of the indicated strains after staining with the anti-CPS antibodies. Scale bar, 2 µm. (**D**) Cells expressing the indicated Cps23BJ or Cps23J-FLAG variants were grown in the BHI medium and normalized to an OD_{600} = 0.3. Proteins were extracted, and the FLAG-tagged proteins were detected by immunoblotting using anti-FLAG antibodies. The black arrow indicates the expected size of the corresponding Cps23BJ-FLAG variants. Cps23BJ^{+} represent the wildtype allele. (**E**) Quantification of the CpsJ variants in strains indicated in (**D**). The intensity of bands was detected and quantified by Fiji (https://fiji.sc). Plotted are the averages and standard deviations of three biological replicates. P-values were calculated by the Student's t-test. n.s., not significant. Shown in panels B, C, and D are representative images from three biological replicates.

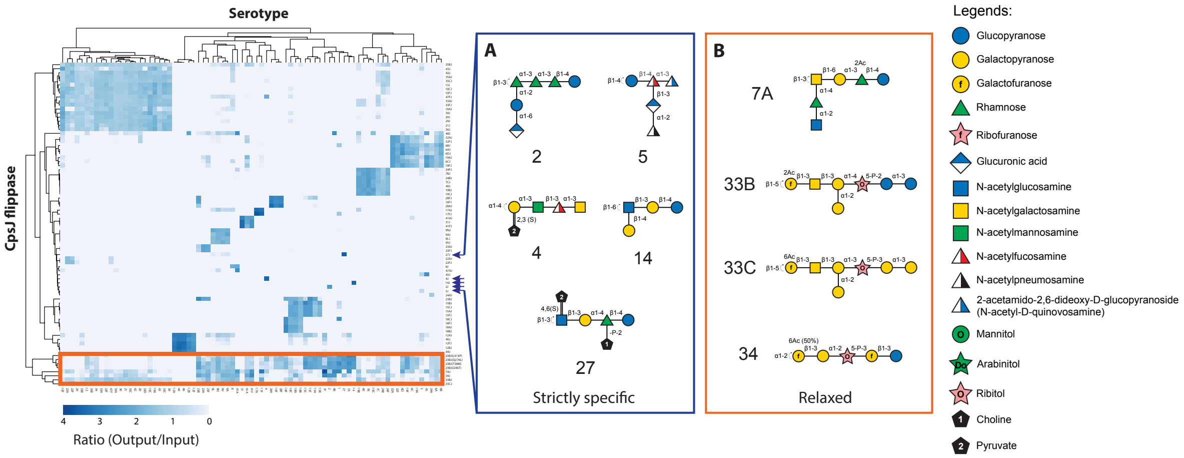

Fig. S2. Capsule flippases can be classified into three groups. 'Type specific' CpsJ flippases likely recognize terminal residues at the non-reducing ends of the CPS precursors, such as ribofuranose or galactofuranose (**Fig. 3**). (**A**) CpsJ flippases that are 'strictly specific' are indicated by blue arrows. The substrates by which they transport are shown in the blue box. CpsJ flippases with relaxed specificities are highlighted with the orange box on the left, and their cognate cargo is shown in (**B**). Examples include Cps7AJ and Cps34J. Glycan structures are drawn in the Symbol Nomenclature for Glycans (SNFG) format (10, 53). The residues at the reducing ends are on the right, whereas the curved arrows on the left represent the polymerization sites.

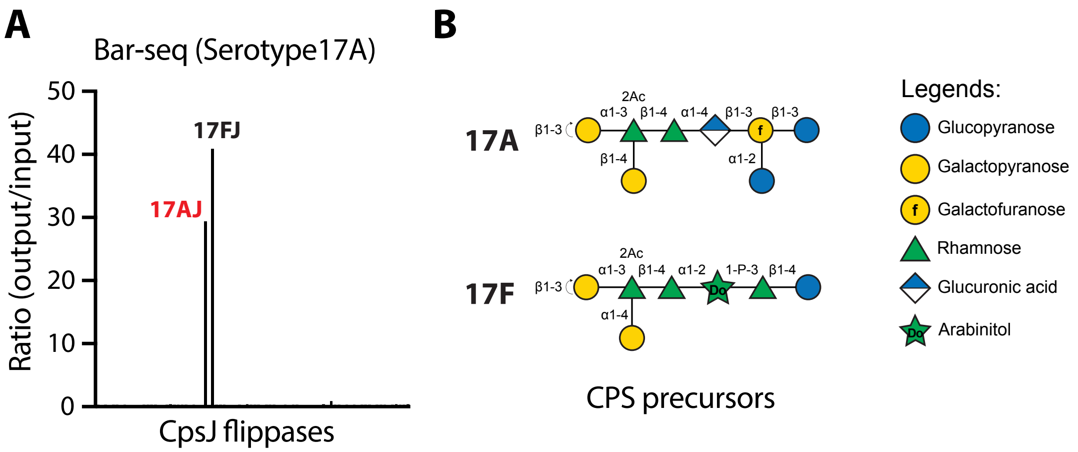

Fig. S3. Cps17AJ can only be complemented by itself and Cps17FJ. (**A**) Shown is the result of barcode sequencing depicting the number of reads obtained before (input) and after (output) the native Cps17AJ was deleted. A high output-to-input ratio indicates complementation by the corresponding ectopic *cpsJ* allele. (**B**) Structures of the serotype 17A and 17F CPSs in the SNFG format (53). The residues at the reducing ends are on the right, whereas the curved arrows on the left represent the polymerization sites.

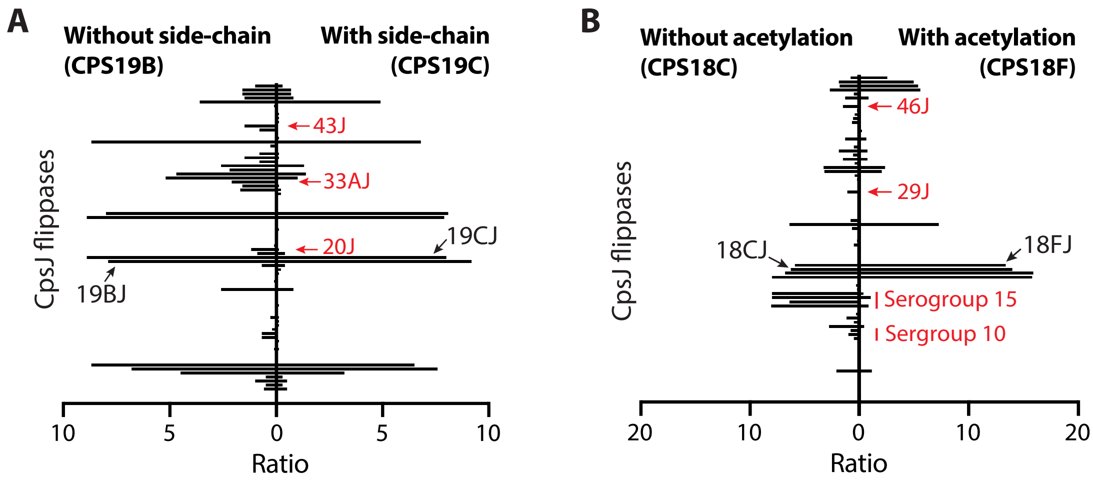

Fig. S4. Side chains and acetylation affect capsule precursor transport. (A) Bar-seq results of serotypes 19B and 19C are shown. The x-axis shows the complementation ratio, calculated by dividing the reads per million reads (RPM) in the output library (after *cpsJ* deletion) by the input library (before *cpsJ* deletion). The y-axis represents the CpsJ flippases of the different serotypes. Non-cognate flippases that complemented Cps19BJ but not Cps19CJ are indicated in red. **(B)** Bar-seq results of serotypes 18C and 18F. The x-axis shows the complementation ratio, calculated by dividing the output library (after *cpsJ* deletion) by the input library (before *cpsJ* deletion). The y-axis represents the CpsJ flippases of the different serotypes. Non-cognate flippases that complemented Cps18CJ but not Cps18FJ are indicated in red.

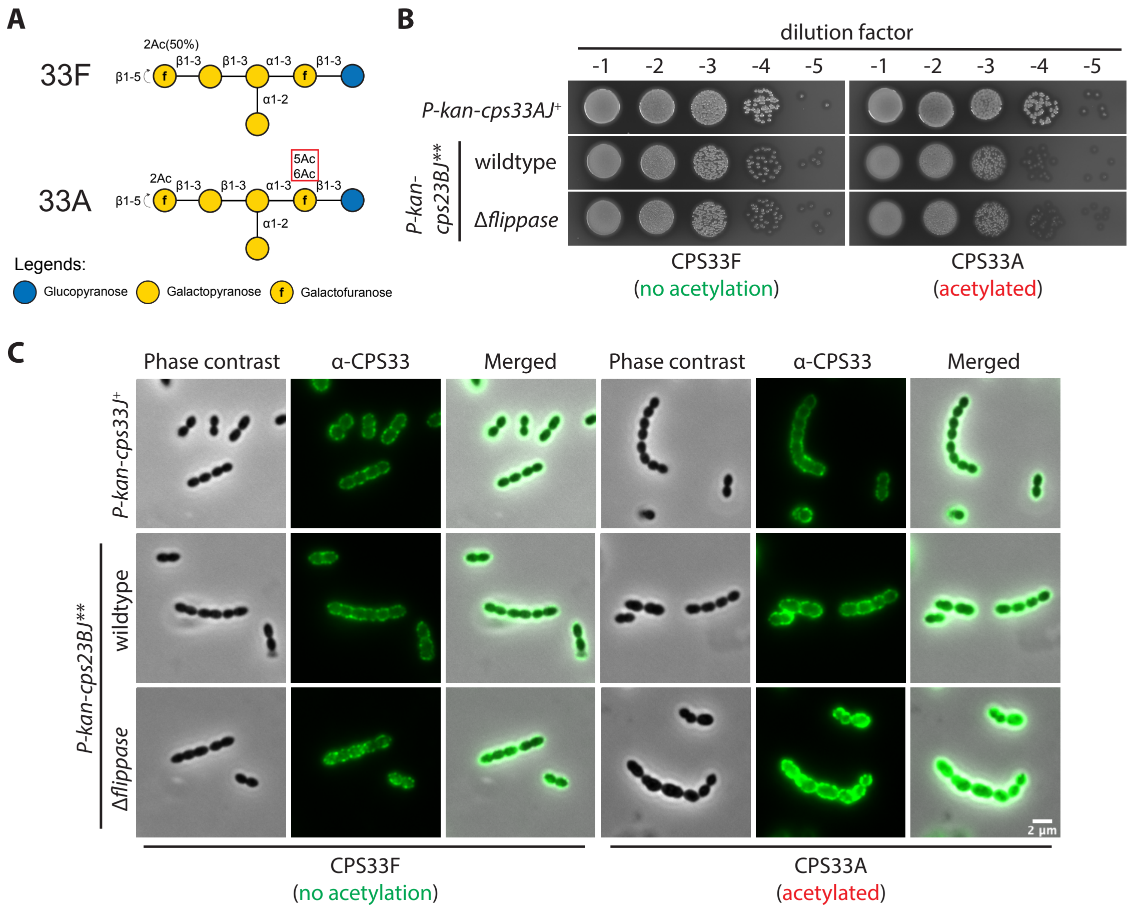

Fig. S5. Cps23BJ is toxic in the serotype 33A but not 33F background.** (**A**) The repeating units of serotypes 33A and 33F CPS differ only in the acetylation at the second galactofuranose residue from the reducing end (indicated by the red box). (**B**) Strains NUS0326 [serotype 33F isogenic capsule-switch mutant; CPS33F], NUS0332 [serotype 33A isogenic capsule-switch mutant; CPS33A], NUS3802 [CPS33F P-*kan-cps23BJ*(P254S, I246T)], NUS3801 [CPS33A P-*kan-cps23BJ*(P254S, I246T)], NUS3904 [CPS33F Δ*cps33FJ* P-*kan-cps23BJ*(P254S, I246T)], and NUS3903 [CPS33A Δ*cps33AJ* P-*kan-cps23BJ*(I246T)] were grown in BHI broth at 37°C in 5% CO_2 until they reached the early exponential phase. Cultures were serially diluted and spotted on blood agar. The plates were incubated at 37°C in 5% CO_2 overnight before imaging. (**C**) Cultures of the indicated strains were grown in the BHI medium at 37°C in 5% CO_2 until OD_{600} reaches 0.2. Cells were harvested by centrifugation, washed, and stained with anti-serotype 33 antisera, and the capsule was detected by labeling with Alexa Fluor 488 conjugated anti-rabbit secondary antibodies. Bar, 2 µm. Shown in panels B and C are representative images from three biological replicates.

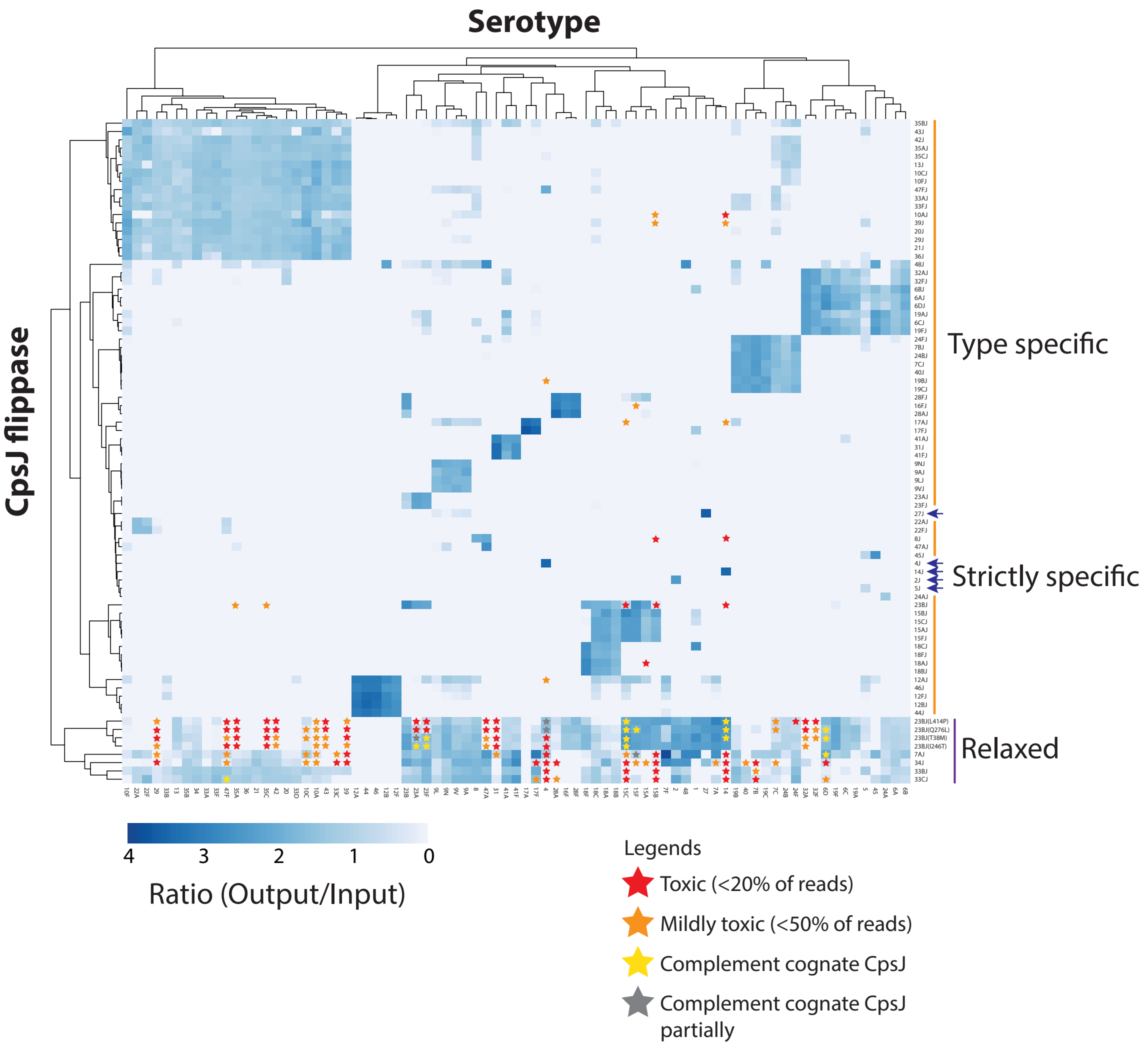

Fig. S6. Flippases with relaxed specificities are toxic in some serotypes. Depicted is a heatmap illustrating the cross-complementation events after the native *cpsJ* has been deleted (see **Fig. 3**). Fitness of the merodiploid mutants (before the native *cpsJ* deletion) carrying various flippases alleles are inferred by the changes in the relative abundance of the barcodes (see **Data S1**), which is calculated by the reads per million reads (RPM) of the indicated strain over the averages of all capsule switch strains. CpsJ alleles that lead to a five-fold decrease in the barcode abundance are defined as toxic (red), whereas those that result in a two-fold decrease are partially toxic (orange). Nevertheless, while some of these *cpsJ* alleles cause growth defects, they could complement the cognate *cpsJ* (yellow). Some of them can partially complement each other, and they are colored grey.

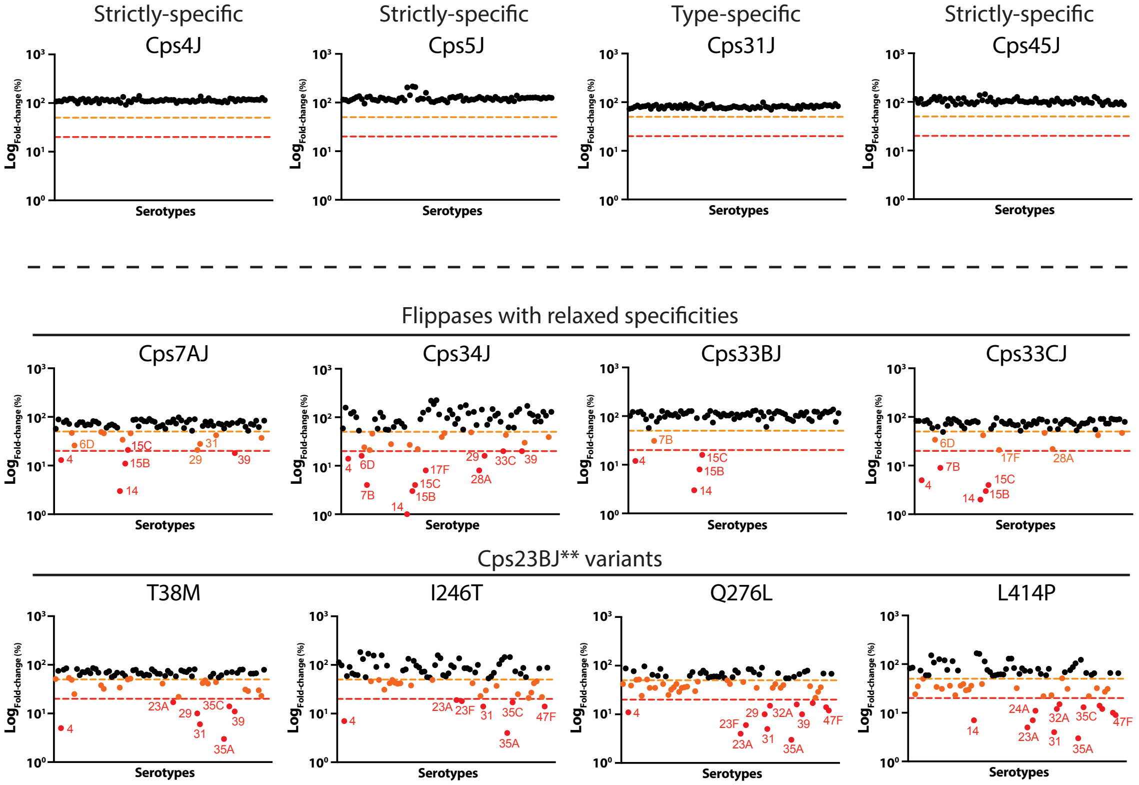

Fig. S7. Examples of toxic *cpsJ* alleles. The degree of toxicity of the *cpsJ* alleles in each CPS-switch mutant was determined by changes in the barcode abundance, as indicated in the legend of **Fig. S6**. A reduction in this number indicates a decrease in fitness. This scenario is common in strains harboring flippase alleles with relaxed specificity (i.e., Cps7AJ, Cps33BJ, Cps33CJ, Cps34J, and Cps23BJ**). The orange line indicates a two-fold reduction, and the red line indicates a five-fold reduction.

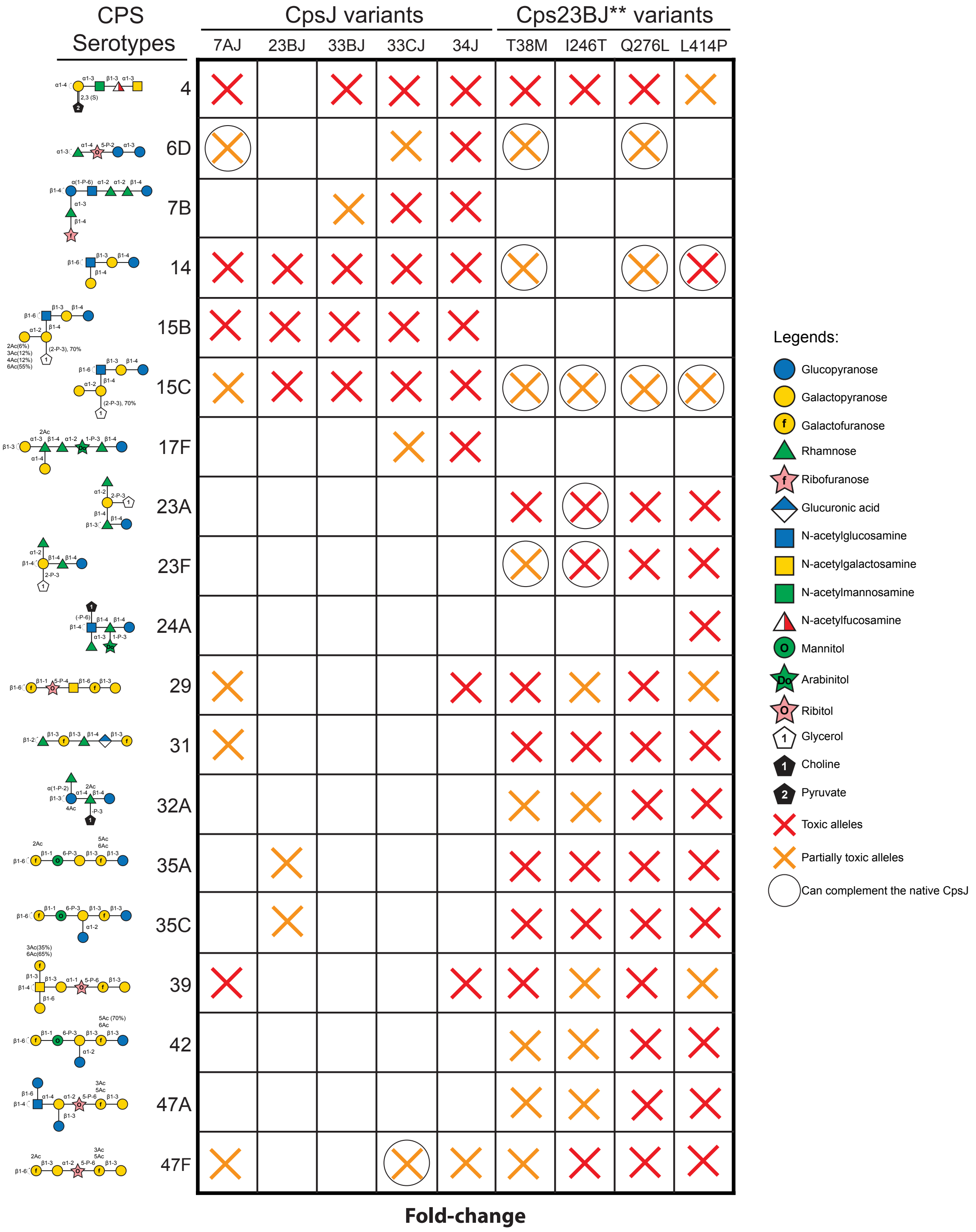

Fig. S8. Structure of the CPS that resulted in a growth defect when expressed in strains harboring the indicated *cpsJ* alleles. The toxic *cpsJ* alleles and the serotypes in which they caused growth defects are tabulated. Orange crosses indicate a two-fold decrease in fitness, while red crosses depict a five-fold decrease. The fitness was determined by the change in RPM of the *cpsJ* alleles in the indicated serotypes compared to the average across all serotypes. The black circles highlight the *cpsJ* alleles that can compensate for the function of the cognate CpsJ.

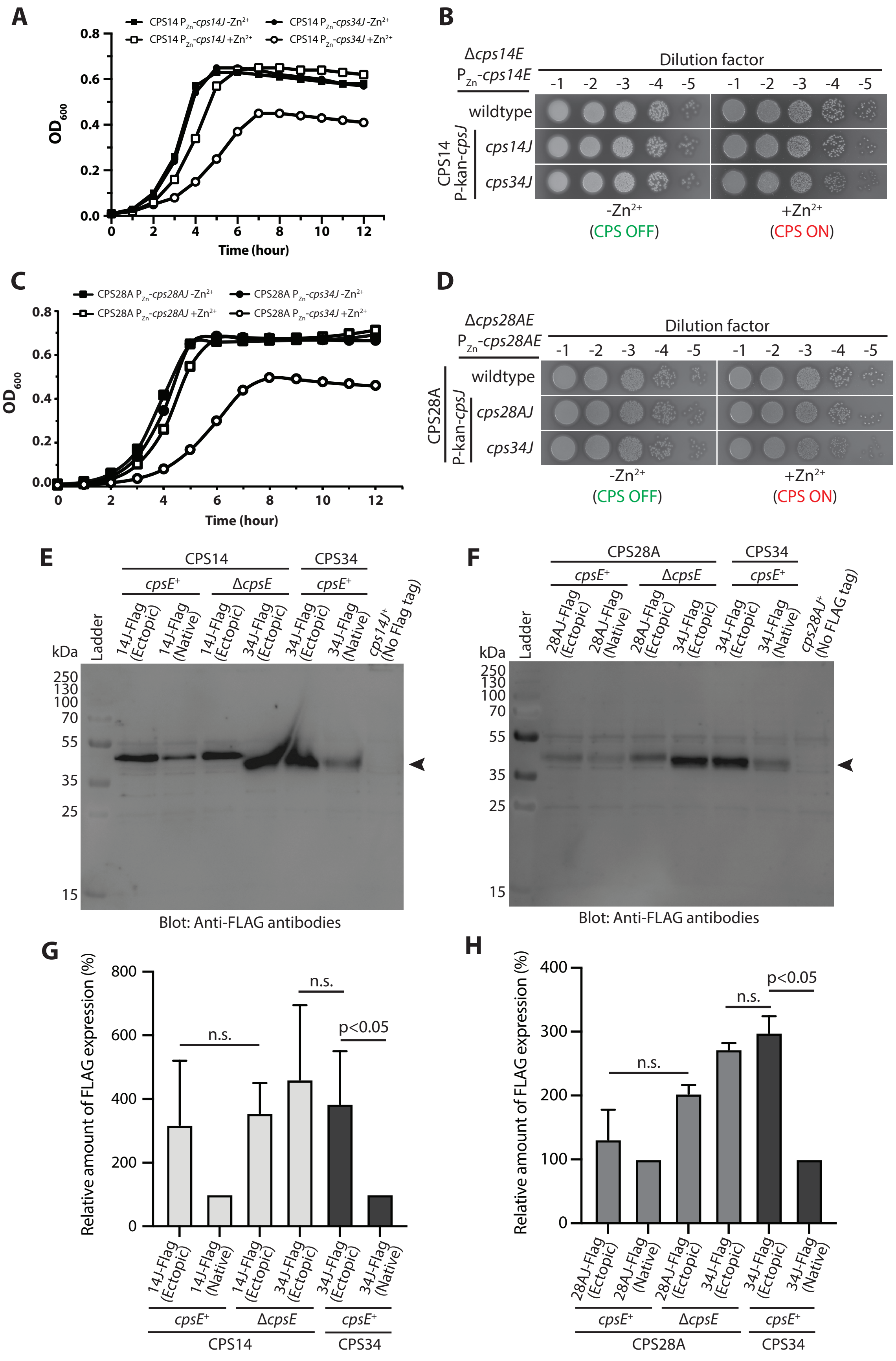
A
B
C
D
E
F
G
H
CPS14 P_{Zn}-cps14J -Zn^{2+}
CPS14 P_{Zn}-cps14J +Zn^{2+}
CPS14 P_{Zn}-cps34J -Zn^{2+}
CPS14 P_{Zn}-cps34J +Zn^{2+}
OD_{600}
Time (hour)
Δcps14E
P_{Zn}-cps14E
Dilution factor
-1 -2 -3 -4 -5
wildtype
cps14J
cps34J
CPS14 P-kan-cpsJ
-Zn^{2+} (CPS OFF)
+Zn^{2+} (CPS ON)
CPS28A P_{Zn}-cps28AJ -Zn^{2+}
CPS28A P_{Zn}-cps28AJ +Zn^{2+}
CPS28A P_{Zn}-cps34J -Zn^{2+}
CPS28A P_{Zn}-cps34J +Zn^{2+}
Δcps28AE
P_{Zn}-cps28AE
cps28AJ
CPS28A P-kan-cpsJ
CPS14
CPS34
CPS28A
cpsE+
ΔcpsE
Ladder
kDa
14J-Flag (Ectopic)
14J-Flag (Native)
34J-Flag (Ectopic)
34J-Flag (Native)
cps14J+ (No Flag tag)
28AJ-Flag (Ectopic)
28AJ-Flag (Native)
cps28AJ+ (No FLAG tag)
250 130 100 70 55 35 25 15
Blot: Anti-FLAG antibodies
Relative amount of FLAG expression (%)
n.s.
p<0.05

Fig. S9. Expression of Cps34J is toxic in the serotype 14 and 28A background. (**A**) Strains NUS4643 [CPS14 P_{Zn}-*cps14J*] and NUS4644 [CPS14 P_{Zn}-*cps34J*] were grown in BHI at 37°C in 5% CO_2. Cells were diluted to OD_{600} of 0.01 in BHI with or without added Zn^{2+}/Mn^{2+} and continued to grow at 37°C. Growth was monitored by measuring the OD_{600}. (**B**) Strains NUS0403 [CPS14], NUS4910 [CPS14 Δ*cps14E* P_{Zn}-*cps14E* P-kan-*cps14J*] and NUS4911 [CPS14 Δ*cps14E* P_{Zn}-*cps14E* P-kan-*cps34J*] were grown in BHI. Cultures were normalized by their optical densities, serially diluted, and spotted on blood agar without (left) or with (right) $ZnCl_2$ and $MnCl_2$. Plates were imaged after overnight incubation at 37°C in 5% CO_2. **(C)** Strains NUS4285 [CPS28A P_{Zn}-*cps28AJ*] and NUS4207 [CPS28A P_{Zn}-*cps34J*] were grown in BHI and their growth was monitored as described in (**A**). (**D**) Strains NUS0671 [CPS28A], NUS4599 [CPS28A Δ*cps28AE* P_{Zn}-*cps28AE* P-kan-*cps28AJ*] and NUS4600 [CPS28A Δ*cps28AE* P_{Zn}-*cps28AE* P-kan-*cps34J*] were grown in BHI and spotted on blood agar plates as described in (**B**)**.** The Cps14J, Cps28AJ and Cps34J variants are stable in serotype 14 (**E**) and 28A (**F**). Cells expressing the indicated CpsJ or CpsJ-FLAG variants were grown in the BHI medium and normalized to an OD_{600} of 0.3. Protein samples were prepared by boiling the cells in 2x Laemmli sample buffer, and the FLAG-tagged proteins were detected by immunoblotting using anti-FLAG antibodies. The black arrow indicates the expected size of the corresponding CpsJ-FLAG variants. Quantification of the CpsJ variants in serotypes 14 (**G**) and 28A (**H**) indicated in (**E**) and (**F**), respectively. Plotted are the averages and standard deviations of three biological replicates. P-values were calculated using the Student's t-test. n.s., not significant. Shown in panels B, D, E, and F are representative images from three biological replicates.

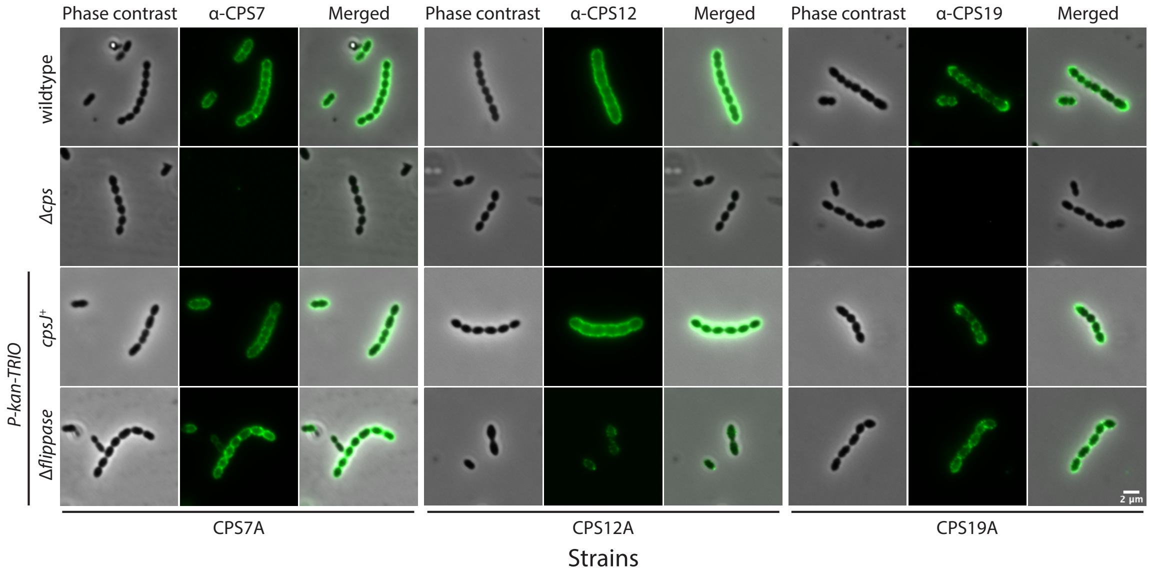

Fig. S10. The TRIO cassette is functional. Strains NUS0667 [serotype 7A capsule-switch mutant; CPS7A], NUS0669 [serotype 12A capsule-switch mutant; CPS12A], NUS0282 [serotype 19A capsule-switch mutant; CPS19A], NUS0114 [Δ*cps*], NUS4961 [CPS7A P-*kan-TRIO*], NUS4770 [CPS12A P-*kan-TRIO*], NUS4771 [CPS19A P-*kan-TRIO*], NUS5031 [CPS7A Δ*cps7AJ*::P-*erm* P-*kan-TRIO*], NUS5412 [CPS12A Δ*cps12AJ*::P-*erm* P-*kan-TRIO*], and NUS4823 [CPS19A Δ*cps19AJ*::P-*erm* P-*kan-TRIO*] were grown in the BHI medium at 37ºC in 5% CO_2 until OD_{600} reaches 0.2. Cells were collected by centrifugation, washed, and stained with anti-serogroup 7 (α-CPS7), anti-serogroup 12 (α-CPS12), or anti-serogroup 19 (α-CPS19) antisera, respectively. The capsule was detected by labeling with Alexa Fluor 488 conjugated anti-rabbit secondary antibodies. Bar, 2 µm. Shown are representative images from two biological replicates.

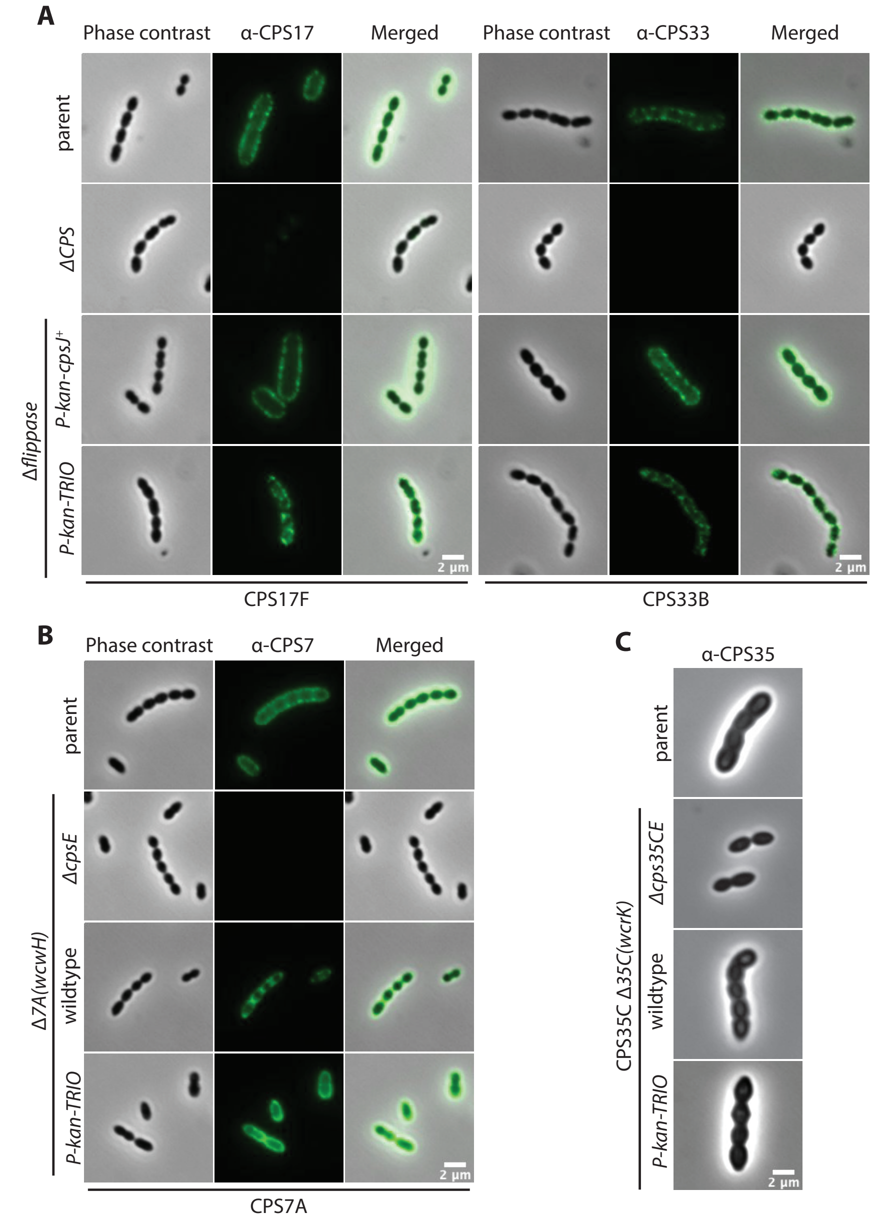

Fig. S11. The TRIO cassette complements Cps17FJ and Cps33BJ. (**A**) Strains NUS0114 [Δ*cps*], NUS0313 [serotype 17F capsule-switch mutant (CPS17F)], NUS5562 [CPS17F Δ*cps17FJ* P-*kan-cps17FJ*+], NUS5563 [CPS17F Δ*cps17FJ* P-*kan-TRIO*], NUS0308 [serotype 33B capsule-switch mutant (CPS33B)], NUS5579 [CPS33B Δ*cps33BJ* P-kan-*cps33BJ*+], NUS5561 [CPS33B Δ*cps33BJ* P-*kan-TRIO*] were grown in the BHI medium at 37°C in 5% CO_2 until OD_{600} reaches 0.2. Cells were harvested by centrifugation, washed, and stained with anti-serogroup 17 (α-CPS17) or anti-serogroup 33 (α-CPS33), respectively. The capsule was detected by labeling with Alexa Fluor 488 conjugated anti-rabbit secondary antibodies. Bar, 2 µm. (**B**) *wcwH* in serotype7A is dispensable for growth. Strains NUS0667 [serotype 7A capsule-switch mutant (CPS7A)], NUS5564 [Cps7A Δ*wcwH*], NUS5565 [CPS7A Δ*cps7AE* Δ*wcwH*], and NUS5566 [Cps7A Δ*wcwH* P-kan-*TRIO*] were grown in the BHI medium at 37°C in 5% CO_2 until OD_{600} reaches 0.2. Cells were harvested by centrifugation, washed, and stained with anti-serogroup 7 (α-CPS7),

followed by labeling with Alexa Fluor 488 conjugated anti-rabbit secondary antibodies. Bar, 2 µm. (**C**) *wcrK* in serotype 35C is dispensable for growth. Strains NUS0369 [serotype 35C capsule-switch mutant (CPS35C)], NUS2399 [CPS35C Δ*wcrK*], NUS5634 [CPS35C Δ*cps35CE* Δ*wcrK*], and NUS2190 [CPS35C Δ*wcrK* P-kan-*TRIO*] were grown in the BHI medium at 37°C in 5% CO_2 until OD_{600} reaches 0.2. Cells were harvested by centrifugation, washed, stained with anti-serogroup 35 (α-CPS35), and visualized by phase-contrast microscopy. Bar, 2 µm. Shown are representative images from two biological replicates.

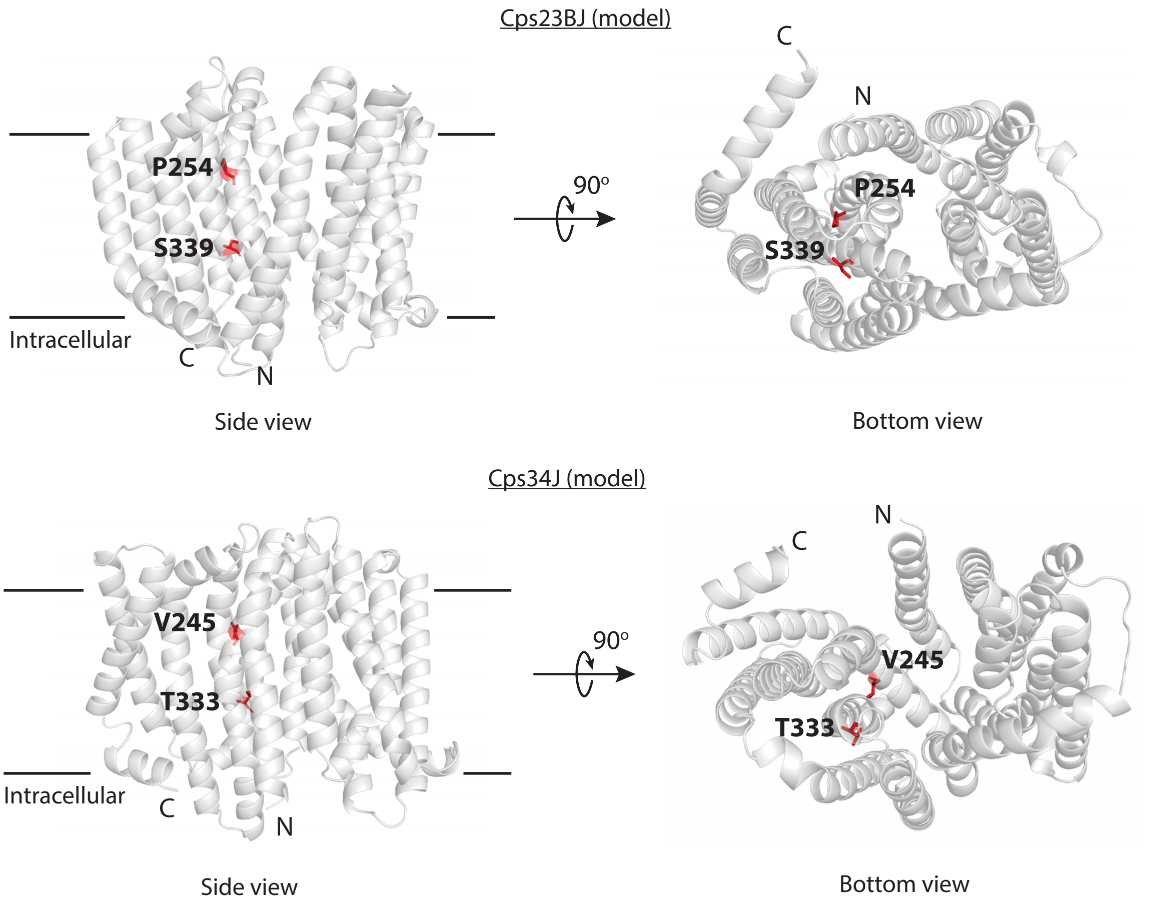

V245(=P254 of Cps23BJ)

T333(=S339 of Cps23BJ)

Fig. S12. Machine learning predicts residues important for governing CpsJ specificity. The structural models of Cps23BJ (Top) and Cps34J (Bottom) generated by AlphaFold2 are shown. Highlighted in red are the amino acid residues associated with cross-complementation (i.e., residues 254 and 339 of Cps23BJ or the equivalent residues).

```
2J      -LSRRYNYLLKNIGLLTLSNFGSKILVFLMVPLYTSVLSTSDYGTYDLFNTTISLLIPII   59
23BJ    --MSKYKELAKNTGTFALANFSSKILIFLLVPIYTKVLTTTEYGFYDLVYTTIQLLVPIL   58
7AJ     --MKTIKNYAYKSF----YQLFLIIVPFITIPYVSRILGAELIGINSYTNTIISYFVLIA   54
14J     MSNKISKNLAYNIG----YQLIGIAFPLITSPYLSRILGAENLGIHSFTISVALYFMMFM   56
35BJ    --MKVLKNYAYNLS----YQLLVIILPIITTPYVTRVFSSNDLGTYGYFNSIVTYFILLA   54
33BJ    --MKVLKNYAYNLS----YQLLVIILPIITTPYVTRVFSSDDLGTYGYFNSIVTYFILLA   54
34J     --MKVLKNYAYNLS----YQLLVIILPIITTPYVTRVFSSDDLGTYGYFNSIVTYFILLA   54
              :    :       ::    . ::  *  : :: :   *  .   :    :: :

2J      SINISEGVLRFALDEKND-SSIVYSIGWNIIIKGFLVVVLGIIFNNIFNIFPLLKENSIT   118
23BJ    TLNISEAVMRFLMKEDVS-KKSVFSIAILDIFLGSIIFCLLLLVNQIFSLSELISQYSIY   117
7AJ     NLGILIYGNRTIAYHR-ESIEERSKKFWEIVSIKLLVAIVAYVIFIIFLFF-YSKYSWVF   112
14J     LLGIANYGNRTIATVKREGKEILSKTFWNIYYVQLLMSVLVTIAYLIYLYFWVSSYKFIA   116
35BJ    TLGVANYGTKVISGHR----KEIEKNFWGIYSLQLGATVFSLTLYCLLCLTLPFMQNPVA   110
33BJ    TLGVANYGTKVISGHR----KQIQKNFLGIYSLQLGATVLSLSLYALLCLTLPFMQNPVA   110
34J     TLGVANYGTKVISGHR----EEIQKNFWGIYSLQLGATVLSLALYVLLCLILPFMQNPVA   110
         :.:     :          .   .              .      :           :

2J      FL-----------LLYLSTIVYQFLSSFIRGID--KVSILSIAAILNTISILGFNILFLI   165
23BJ    IM-----------AIFAFYTLNNFLIQYSKGID--KIGVTAISGVISAAVMLSMNILLLV   164
7AJ     VIQSVQIIATAFDISWLFDGVEDFKRTVVRNFLVKIISIILIFTFVKSTE----------   162
14J     ILQLFLLLSNAVDITWLFYGLEDFKQIVFRNALVKLLGLFLIFSFVHESS----------   166
35BJ    YILGLALVSKGLDISWLFQGLEDFRKITVRNITVKFVGVISIFLFVKSAD----------   160
33BJ    YILGLSLVSKGLDISWLFQGLEDFRKITVRNITVKLVGVISIFLFVKSSN----------   160
34J     YILGLSLVSKGLDISWLFQGLEDFRKITVRNITVKLVGVISIFLFVKSAN----------   160
         :             :    : :*     :.     :.:  *  .:

2J      IIPLGLVGYFWSNILGLVLPSLYLIYKISQYNIKYTSL----QNKKLQQRLVSYSIPLIL   221
23BJ    VLNWGLLGFFIANICGYVIPCVYIIVKLKLWDLFEL-K----IDRSLQWEMIYYTLPLIL   219
7AJ     ----DFDKYIWITV------GSTLMGNLTLWSYLHHYIIKIPIKSLKLSEHLVPILTLFI   212
14J     ----DLWKYTLING------GVTLVGQLLLWGQLKGRLSWVKIQKKDLLSHIKPILVLFI   216
35BJ    ----DLYLYVFLLT------IFELLGQLSMWVPAREFIGRPHFDLEYARHHLKPVILLFL   210
33BJ    ----DLYLYVFLLT------IFELFGQFSMWIPAREFIGRPHFDIEYARHHLKPVILLFL   210
34J     ----DLYLYVFLLT------IFELLGQLSMWLPAREFIDRPHFDLEYAKPHLKPVILLFL   210
            .:  :              :. ::  :            .       :   : *::

                                          254
2J      NSLGWWINNAIDRYVVIAFCGVAVNGIYSVGYKIPSILNIFANIFNQA---WILSSVKSY   278
23BJ    NTLSWWVNNTSDRYIITVIIGIQASAIISVAYKIPQIFSTISAIFIQS---WQISAIKIQ   276
7AJ     PQIASIVFMSINKILLGNISTISQAGYFENADKVIRILLALVSSIGVVVFPKVAHAYRSG   272
14J     PVLAISIFSNMDKYMLGLMVGVKQVGFYDNANRIIDIPKALIAALEAVMLPRTSYLLAEG   276
35BJ    PQVAISLYVTLDRTMLGALASTKDVGIYDQALKLVNILLTLVTSLGSVMLPRVSNLLSSG   270
33BJ    PQVAISLYVTLDRTMLGALASTKDVGIYDQALKLVNILLTLVTSLGSVMLPRVAHLLATD   270
34J     PQVAISLYVTLDRTMLGALASTKDVGIYDQALKLVNILLTLVTSLGSVMLPRVANLLATG   270
          :.  :    :: ::  :      .  . . ::  *   :   :

2J      RDEDSEYFFSQVYNKYNMIMVLISGLLISCSKILAKFLYMNEFYDAWKFVPFLLIANVFG   338
23BJ    EEKEGNTFISKMLLYYNALLLIIASGIILFVKPISNILFGASFYSAWTLVPFLIISSLFN   336
7AJ     DMKRVLGLTYMTFDAVNIITIPIVVGIVSISPTFSSIFFGTEFQGIDKVLSVLVLELIFM   332
14J     QEEKSNYYIEVTILYAMMISSVLIFGIISVSDIFSLVFWGEEFLESGRLIAAMAPVFVFS   336
35BJ    DHKAVNKMHEISFLIYNLVIFPIMAGMLIVNDDFVTFFLGQDFQEARYAIAIMIFRMFFI   330
33BJ    DHKAVNRMHEMSFFIYNLVIFPMMAGILIVNDDFVQFFLGQDFQDARYAIAIMIFRMFFI   330
34J     NHRAVNKMHEISFLIYNLVIFPMMAGILIVNDDFVQFFLGQDFQDARYAIAIMSFRMFFI   330
          .               :   :   ::     :  .:   .*      :  :    .*

          339
2J      AISGFAGGI-FSAVKDSKIYSQSTLVGAIVNIIFTFVFVYYYGAIGAAIATMISYFVVWI   397
23BJ    AISGYIGAI-MGAKMDTKNIAKSALVGMIANVFLNIVLTFLMGLQGITISTMIASFLIFY   395
7AJ     GYTSVLGSQYLIVTGQTYFLSISVFLGIFSTVISSFFFIPIYGALGSAISSVIGEASIMI   392
14J     VPGNIIRTQYLIPRAKDKDYVLSLIIGALVNILLNCFLIKPFGAMGATISTVLAEFVLYG   396
35BJ    GWTNIMGIQILIPHNQNKEFMLSTTIPAIVSVGLNLLLLPKFGYIGAAIVSVLTEALVWA   390
33BJ    GWTNIMGIQMLIPHNQNKEFMISTTAPAIISVGLNLLFLPKLGYIGAAIVSVLTEALVWA   390
34J     GWTNIMGTQILIPHNQNKEFMISTTAPAIVSVGLNLLFLPKLGYIGAAIVSVSTEALVWA   390
           .      :    .      *     : .:  . .:    *  * :* ::     :

2J      IRVHTMRKYIKLKIFIRRDVFSYVLLIFQS-IVLWLENSYILYP----------IQVVLF   446
23BJ    MRKDSVEEIAPETY--RAIYLSWFLLVVEASLLVY-----IDFI----------IGATLV   438
7AJ     GEIYLLRNQVDFYYLYRDVPKYMIASAVMY-ISISSL-NYFISSPFVSLLSSIAMGAVTY   450
14J     VQFWTVRRDLDFKKYLKNGFIFYLFGMIMY-LAIIAAKAHLQYN-IINLVLLIVLGGIVY   454
35BJ    IQLYYIRAYLKDVPIIRTMIKIILASAIMY-SVLLVSKTYIHFSPTINVLVFVVLGGIIY   449
33BJ    IQLFYTRRYLKEVPIIGSMSKIILASAIMY-GLLLSSKTVIHFSPTLNVLAFAALGGIIY   449
34J     IQLYFTRRYLKEVPIIGSLTKIVLASAIMY-GILLGSKTLIQFSPTINVIVFAVLGGIIY   449
         .    .                .   .     :      :             :

2J      LLLVM-L-------FYKEIKSIIGELKKFLT*--   469
23BJ    TLINLFL-------LKDTLKPLCLKLLKGFK*--   462
7AJ     VTVVLLL--CPRI-VI----KLLNKNTRFF*---   473
14J     TGFCCFYILISRNVHFEILREKIKRKIGYENIL*   487
35BJ    LFSV----LSLKVVDVIELKQVIRKN*-------   471
33BJ    LFAI----LSLKVVDVKELKQIIRKN*-------   471
34J     LFAI----LSLKVVDIKELKHIIKK*--------   470
                                .
```

Legend

Strictly-specific
Type-specific
Relaxed

Fig. S13. Residues that may determine flippases specificity. Amino acid sequences of the strictly specific (i.e. Cps2J, Cps14J), type-specific (i.e. Cps23BJ, Cps35BJ), and relaxed flippases (i.e. Cps7AJ, Cps33BJ, Cps34J) were aligned using Clustal Omega (https://www.ebi.ac.uk/jdispatcher/msa/clustalo) and were highlighted in blue, red, and yellow respectively. The red letters denote the differences of residues at positions equivalent to 254 and 339 of Cps23BJ.

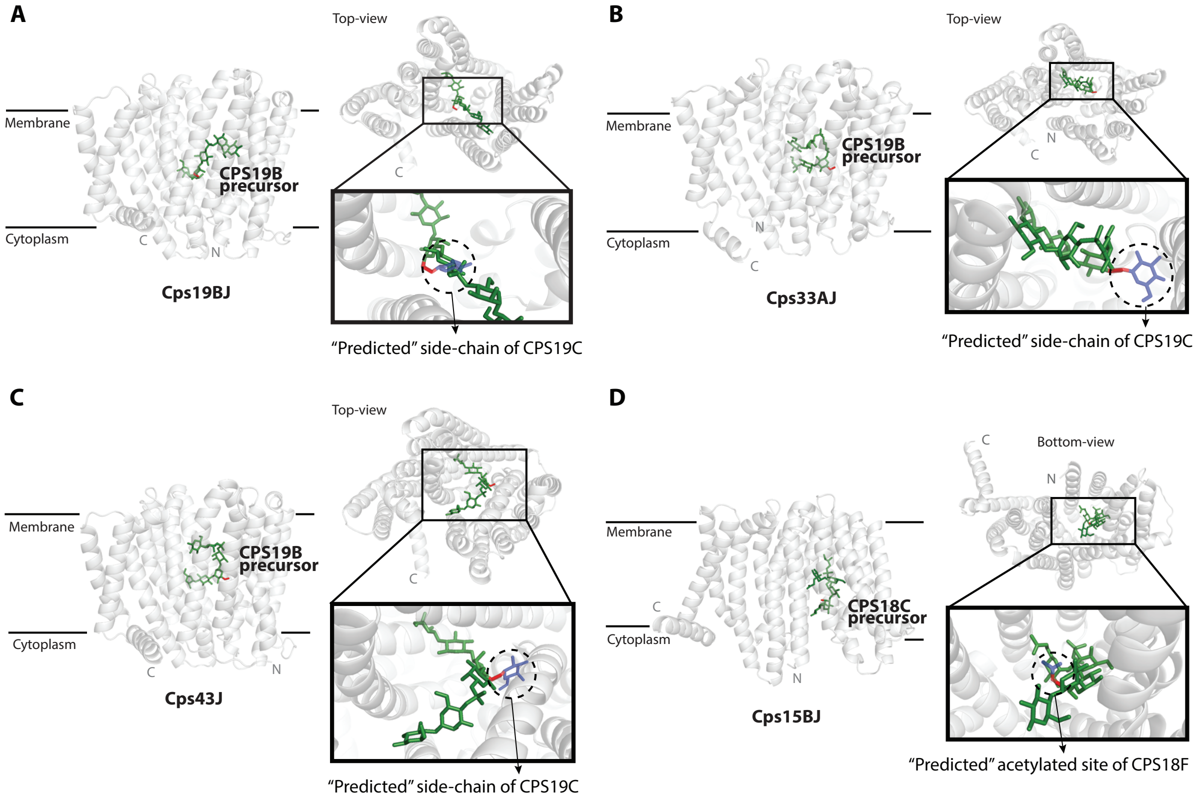

Fig. S14. Docking of serotype 19B CPS precursors to the structural model of (**A**) Cps19BJ and (**B**) Cps33AJ and (**C**) Cps43J, respectively. The glucose residue on the side chain of serotype 19C precursor is predicted to clash with the transmembrane helix of Cps33AJ and Cps43J, but not Cps19BJ. (**D**) Docking of serotype 18C precursor to the structural model of Cps15BJ flippase. The acetylated site on serotype 18F precursor is predicted to face the central aqueous cavity. Oligosaccharides and the flippase sequences of the indicated serotypes were retrieved from the Bacterial Carbohydrate Structure Database (CSDB) and the National Center for Biotechnology Information (NCBI), respectively. The docking experiments were performed using Chai-1 (https://lab.chaidiscovery.com).

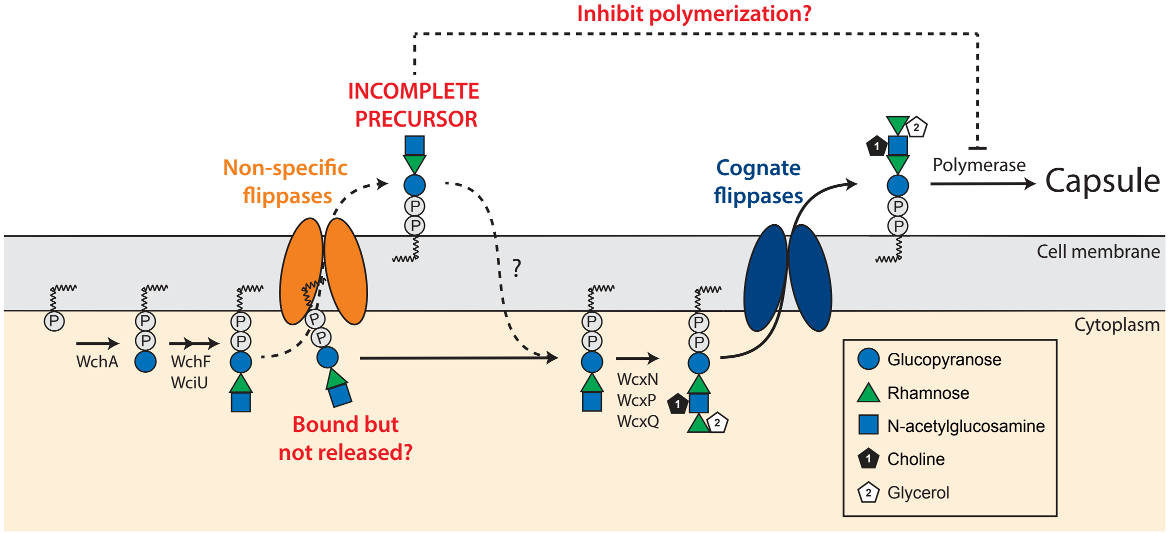

Fig. S15. A model that illustrates the importance of flippase specificity in the cell. Depicted is the serotype 28A CPS pathway. WchA installs the first glucose residue to C55-P. Other glycosyltransferases and synthetic enzymes (WchF, WciU, WcxN, WcxP, and WcxQ) add other residues to the glucosylated C55-P to produce the repeating unit. The lipid-linked product is then flipped and polymerized. A flippase with relaxed specificity may compete with the glycosyltransferase for the incomplete lipid-linked precursors. As they are structural homologs of the completed substrates, they may inhibit downstream enzymes such as the polymerase. Alternatively, the flippase may bind to the lipid-linked precursors non-specifically. Either way, the pathway is stalled, leading to the sequestration of C55-P and killing the cell.

Table S1. Cps23BJ** variants do not transport lipid-linked teichoic acid precursors

**Genotype of recipient cells**	**Transformants obtained when the indicated amplicon was introduced (CFU)**		
	**None**	**Δ*pgdA*:: P-*erm*[a]**	**Δ*tacF*:: P-*erm***
rpsL1 Δ*cps2J*<>*tacF* Δ*bgaA*::P_{Zn}-*cps2J*	-	-	>300
rpsL1 Δ*bgaA*::P-*kan-cps23BJ*(P254S, V249A)	0	>300	7[b]
rpsL1 Δ*bgaA*::P-*kan-cps23BJ*(P254S, G347V)	0	>300	6[b]
rpsL1 Δ*bgaA*::P-*kan-cps23BJ*(P254S, L28F)	0	>300	7[b]

"-", not tested.

[a] The Δ*pgdA*::P-*erm* cassette was used as a control for transformation efficiency. *pgdA* encodes a dispensable peptidoglycan N-acetylglucosamine deacetylase.

[b] Diagnostic PCR was performed and showed that *tacF* was not deleted in these isolates.

Table S2. Cps23BJ** variants transport the cognate precursor

**Genotype of recipient cells**	**Transformants obtained when the indicated amplicon was introduced (CFU)**	
	**None**	**Δ*cps23BJ*::P-*erm***
rpsL1 CPS23B (a capsule-switch mutant of serotype 23B; NUS0329)	-	0
rpsL1 CPS23B Δ*bgaA*::P-*kan*-*cps23BJ*⁺	0	>300
rpsL1 CPS23B Δ*bgaA*::P-*kan*-*cps23BJ* (P254S, V249A)	-	>300
rpsL1 CPS23B Δ*bgaA*::P-*kan*-*cps23BJ* (P254S, G347V)	-	>300
rpsL1 CPS23B Δ*bgaA*::P-*kan*-*cps23BJ* (P254S, L28F)	-	>300

"-" denotes that the transformation reaction was not tested.

Table S3. Cps23BJ** variants transport the serotype 2 precursor

Genotype of recipient cells	Transformants obtained when the indicated amplicon was introduced (CFU)	
	**None**	**Δ*cps2J*::P-*erm***
rpsL1 (strain IU1781, serotype 2)	-	0
rpsL1 Δ*bgaA*::P-*kan-cps23BJ*(P254S, I246T)	0	>300
rpsL1 Δ*bgaA*::P-*kan-cps23BJ*(P254S, V249A)	-	>300
rpsL1 Δ*bgaA*::P-*kan-cps23BJ*(P254S, G347V)	-	>300
rpsL1 Δ*bgaA*::P-*kan-cps23BJ*(P254S, L28F)	-	>300

"-" denotes that the transformation reaction was not tested.

Table S4. Some Cps23BJ** variants transport the serotype 14 precursor

**Genotype of recipient cells**	**Transformants obtained when the indicated amplicon was introduced (CFU)**	
	**None**	**Δ*cps14J*::P-*erm***
rpsL1 CPS14 (a capsule-switch mutant of serotype 14; NUS0403)	-	5[a]
rpsL1 CPS14 Δ*bgaA*::P$_{SpxB}$-*cps23BJ* (P254S)	-	3[a]
rpsL1 CPS14 Δ*bgaA*::P-*kan*-*cps23BJ* (P254S, I246T)	0	>300
rpsL1 CPS14 Δ*bgaA*::P-*kan*-*cps23BJ* (P254S, V249A)	-	>300
rpsL1 CPS14 Δ*bgaA*::P-*kan*-*cps23BJ* (P254S, G347V)	-	20[a]
rpsL1 CPS14 Δ*bgaA*::P-*kan*-*cps23BJ* (P254S, L28F)	-	>300

"-", not tested.

[a] Diagnostic PCR was performed and showed that *cps14J* was not deleted in these isolates.

Table S5. Cps33DJ at the ectopic locus is non-functional.

Genotype of recipient cells	Transformants obtained when the indicated amplicons was introduced (CFU)		
	**None**	**Δ*bgaA*::P-*erm*[a]**	**Δ*cps33DJ*::P-*erm***
rpsL1 CPS33D (a capsule-switch mutant of serotype 33D; NUS0672)	-	>300	0
rpsL1 CPS33D Δ*cps33DE::*P-*spec-rpsL*$^+$	-	-	>300
rpsL1 CPS33D ΔCEP::P-*kan-cps33DJ*$^+$	0	>300	0

"-", not tested.

[a] The Δ*bgaA*::P-*erm* cassette was used as a control for transformation efficiency. *bgaA* encodes a dispensable beta-galactosidase that is unrelated to capsule synthesis.

Table S6. Relaxed flippase variants do not transport lipid-linked teichoic acid precursors

**Genotype of recipient cells**	**Transformants obtained when the indicated amplicon was introduced (CFU)**		
	**None**	**Δ*pgdA*:: P-*erm*[a]**	**Δ*tacF*:: P-*erm***
rpsL1 Δ*CEP*::P_{Zn}-*tacF*	-	-	>300
rpsL1 Δ*CEP*::P-*kan-cps7AJ-barcode*	0	>300	1[b]
rpsL1 Δ*CEP*::P-*kan-cps33BJ-barcode*	0	>300	7[b]
rpsL1 Δ*CEP*::P-*kan-cps33CJ-barcode*	0	>300	4[b]
rpsL1 Δ*CEP*::P-*kan-cps34J-barcode*	0	>300	5[b]

"-", not tested.
[a] The Δ*pgdA*::P-*erm* cassette was used as a control for transformation efficiency. *pgdA* encodes a dispensable peptidoglycan N-acetylglucosamine deacetylase.
[b] Diagnostic PCR was performed and showed that *tacF* was not deleted in these isolates.

Table S7. Side-chain of CPS19C affects complementation.

Genotype of recipient cells	Transformants obtained when the indicated amplicon was introduced (CFU)	
	Δ*cps19BJ::*P*-erm*	Δ*cps19CJ::*P*-erm*
rpsL1 CPS19B P-*kan-cps19BJ*	>300	-
rpsL1 CPS19B P-*kan-cps20J*	>300	-
rpsL1 CPS19C P-*kan-cps19CJ*	-	>300
rpsL1 CPS19C P-*kan-cps20J*	-	7[a]

"-", not tested.

[a] These clones are likely suppressors because the cells were unencapsulated.

Table S8. Cps34J is toxic in serotypes 14 and 28A

Genotype of recipient cells	Transformants obtained when the indicated amplicons was introduced (CFU)		
	**None**	**Δ*CEP*::P-*kan*-*cps14J***	**Δ*CEP*::P-*kan*-*cps34J***
rpsL1 CPS14	0	>300	1[a]
rpsL1 CPS14 Δ*cps14E*::P-*erm*	-	-	>300

Genotype of recipient cells	Transformants obtained when the indicated amplicons was introduced (CFU)		
	**None**	**Δ*CEP*::P-*kan*-*cps28AJ***	**Δ*CEP*::P-*kan*-*cps34J***
rpsL1 CPS28A	0	>300	3[a]
rpsL1 CPS28A Δ*cps28AE*::P-*erm*	-	-	>300

"-", not tested.

[a] Mutations were acquired within the Cps34J flippase region. For CPS14: stop codon. For CPS28A: Incorrect band size for diagnostic PCR; G130V; and G236W.

Table S9. The TRIO cassette could transport cognate and non-cognate substrates.

Genotype of recipient cells	Transformants obtained when the indicated amplicons was introduced (CFU)	
	**None**	**Δ*cps7AJ*::P-*erm***
rpsL1 CPS7A	-	2[a]
rpsL1 CPS7A ΔCEP::P-*kan-cps7AJ*	-	>300
rpsL1 CPS7A ΔCEP::P-*kan-cps7AJ-L-cps12AJ-L-cps19AJ*	0	>300 (tiny)

Genotype of recipient cells	Transformants obtained when the indicated amplicons was introduced (CFU)	
	**None**	**Δ*cps12AJ*::P-*erm***
rpsL1 CPS12A	-	0
rpsL1 CPS12A ΔCEP::P-*kan-cps12AJ*	-	>300
rpsL1 CPS12A ΔCEP::P-*kan-cps7AJ-L-cps12AJ-L-cps19AJ*	0	164

Genotype of recipient cells	Transformants obtained when the indicated amplicons was introduced (CFU)	
	**None**	**Δ*cps19AJ*::P-*erm***
rpsL1 CPS19A	-	3[a]
rpsL1 CPS19A ΔCEP::P-*kan-cps19AJ*	-	214
rpsL1 CPS19A ΔCEP::P-*kan-cps7AJ-L-cps12AJ-L-cps19AJ*	0	>300

Genotype of recipient cells	Transformants obtained when the indicated amplicons was introduced (CFU)	
	**None**	**Δ*cps33BJ*::P-*erm***
rpsL1 CPS33B	-	0
rpsL1 CPS33B ΔCEP::P-*kan-cps33BJ*	-	>300
rpsL1 CPS33B Δ*bgaA*::P-*kan-cps23BJ***	-	0
rpsL1 CPS33B ΔCEP::P-*kan-cps7AJ-L-cps12AJ-L-cps19AJ*	0	>300

**Genotype of recipient cells**	**Transformants obtained when the indicated amplicons was introduced (CFU)**	
	**None**	**Δ*cps17FJ*::P-*erm***
rpsL1 CPS17F	-	42[a]
rpsL1 CPS17F ΔCEP::P-*kan-cps17FJ*	-	>300
rpsL1 CPS17F Δ*bgaA*::P-*kan-cps23BJ***	-	>300 (tiny)[b]
rpsL1 CPS17F ΔCEP::P-*kan-cps7AJ-L-cps12AJ-L-cps19AJ*	0	>300

"-", not tested.

[a] Diagnostic PCR demonstrated that the corresponding amplicons were not introduced in these isolates.

[b] These clones are likely suppressors because the cells did not survive after re-streaking or were unencapsulated.

Table S10. TRIO does not allow deletions of essential GTs

**Genotype of recipient cells**	**Transformants obtained when the indicated amplicons was introduced (CFU)**	
	**None**	**Δ*cps5(whaD)*::P-*erm***
rpsL1 CPS5	-	>300 (tiny)[b]
rpsL1 CPS5 Δ*cps5E::*P-*spec-rpsL*	-	88
rpsL1 CPS5 Δ*bgaA*::P-*kan-cps23BJ***	-	3[a]
rpsL1 CPS5 ΔCEP::P-*kan-cps7AJ-L-cps12AJ-L-cps19AJ*	0	0

**Genotype of recipient cells**	**Transformants obtained when the indicated amplicons was introduced (CFU)**	
	**None**	**Δ*cps7A(wcwH)*::P-*erm***
rpsL1 CPS7A	-	>300 small
rpsL1 CPS7A Δ*cps7AE::*P-*spec-rpsL*	-	>300
rpsL1 CPS7A Δ*bgaA*::P-*kan-cps23BJ***	-	3[a]
rpsL1 CPS7A ΔCEP::P-*kan-cps7AJ-L-cps12AJ-L-cps19AJ*	0	>100 small

**Genotype of recipient cells**	**Transformants obtained when the indicated amplicons was introduced (CFU)**	
	**None**	**Δ*cps18B(wciY)*::P-*erm***
rpsL1 CPS18B	-	0
rpsL1 CPS18B Δ*cps18BE::*P-*spec-rpsL*	-	>300
rpsL1 CPS18B Δ*bgaA*::P-*kan-cps23BJ***	-	3[a]
rpsL1 CPS18B ΔCEP::P-*kan-cps7AJ-L-cps12AJ-L-cps19AJ*	0	2[a]

**Genotype of recipient cells**	**Transformants obtained when the indicated amplicons was introduced (CFU)**	
	**None**	**Δ*cps19C(wchR)*::P-*erm***
rpsL1 CPS19C	-	0
rpsL1 CPS19C Δ*cps19CE::*P-*spec-rpsL*	-	>300

rpsL1 CPS19C Δ*bgaA*::P-*kan-cps23BJ***	-	2[a]
rpsL1 CPS19C ΔCEP::P-*kan-cps7AJ-L-cps12AJ-L-cps19AJ*	0	0

Genotype of recipient cells	Transformants obtained when the indicated amplicons was introduced (CFU)	
	None	Δ*cps23F(wchX)*::P-*erm*
rpsL1 CPS23F	-	0
rpsL1 CPS23F Δ*cps23FE::*P-*spec-rpsL*	-	>300
rpsL1 CPS23F Δ*bgaA*::P-*kan-cps23BJ***	-	27[a]
rpsL1 CPS23F ΔCEP::P-*kan-cps7AJ-L-cps12AJ-L-cps19AJ*	0	3[a]

Genotype of recipient cells	Transformants obtained when the indicated amplicons was introduced (CFU)	
	None	Δ*cps27(whaL)*::P-*erm*
rpsL1 CPS27	-	0
rpsL1 CPS27 Δ*cps27E::*P-*spec-rpsL*	-	>300
rpsL1 CPS27 Δ*bgaA*::P-*kan-cps23BJ***	-	0
rpsL1 CPS27 ΔCEP::P-*kan-cps7AJ-L-cps12AJ-L-cps19AJ*	0	49 (tiny)[b]

Genotype of recipient cells	Transformants obtained when the indicated amplicons was introduced (CFU)	
	None	Δ*cps27(wcrN)*::P-*erm*
rpsL1 CPS27	-	0
rpsL1 CPS27 Δ*cps27E::*P-*spec-rpsL*	-	>300
rpsL1 CPS27 Δ*bgaA*::P-*kan-cps23BJ***	-	22[a]
rpsL1 CPS27 ΔCEP::P-*kan-cps7AJ-L-cps12AJ-L-cps19AJ*	0	10[a]

Genotype of recipient cells	Transformants obtained when the indicated amplicons was introduced (CFU)

	None	Δ*cps32F(wchQ)*::P-*erm*
rpsL1 CPS32F	-	1[a]
rpsL1 CPS32F Δ*cps32FE::*P-*spec-rpsL*	-	>300
rpsL1 CPS32F Δ*bgaA*::P-*kan-cps23BJ***	-	155
rpsL1 CPS32F ΔCEP::P-*kan-cps7AJ-L-cps12AJ-L-cps19AJ*	0	13[a]

**Genotype of recipient cells**	**Transformants obtained when the indicated amplicons was introduced (CFU)**	
	**None**	**Δ*cps32F(wcrN)*::P-*erm***
rpsL1 CPS32F	-	5[a]
rpsL1 CPS32F Δ*cps32FE::*P-*spec-rpsL*	-	>300
rpsL1 CPS32F Δ*bgaA*::P-*kan-cps23BJ***	-	227
rpsL1 CPS32F ΔCEP::P-*kan-cps7AJ-L-cps12AJ-L-cps19AJ*	0	13[a]

**Genotype of recipient cells**	**Transformants obtained when the indicated amplicons was introduced (CFU)**	
	**None**	**Δ*cps33F(wciF)*::P-*erm***
rpsL1 CPS33F	-	1[a]
rpsL1 CPS33F Δ*cps33FE::*P-*spec-rpsL*	-	>300
rpsL1 CPS33F Δ*bgaA*::P-*kan-cps23BJ***	-	2[a]
rpsL1 CPS33F ΔCEP::P-*kan-cps7AJ-L-cps12AJ-L-cps19AJ*	0	3[a]

**Genotype of recipient cells**	**Transformants obtained when the indicated amplicons was introduced (CFU)**	
	**None**	**Δ*cps35C(wcrK)*::P-*erm***
rpsL1 CPS35C	-	>300
rpsL1 CPS35C Δ*cps35CE::*P-*spec-rpsL*	-	>300
rpsL1 CPS35C Δ*bgaA*::P-*kan-cps23BJ***	-	>300
rpsL1 CPS35C ΔCEP::P-*kan-cps7AJ-L-cps12AJ-L-cps19AJ*	0	>300

Genotype of recipient cells	Transformants obtained when the indicated amplicons was introduced (CFU)	
	**None**	**Δ*cps41A(wcrW)*::P-*erm***
rpsL1 CPS41A	-	1[a]
rpsL1 CPS41A Δ*cps41AE::*P-*spec-rpsL*	-	>300
rpsL1 CPS41A Δ*bgaA*::P-*kan-cps23BJ***	-	1[a]
rpsL1 CPS41A ΔCEP::P-*kan-cps7AJ-L-cps12AJ-L-cps19AJ*	0	0

Genotype of recipient cells	Transformants obtained when the indicated amplicons was introduced (CFU)	
	**None**	**Δ*cps41A(wcrQ)*::P-*erm***
rpsL1 CPS41A	-	>100 (tiny)[b]
rpsL1 CPS41A Δ*cps41AE::*P-*spec-rpsL*	-	>300
rpsL1 CPS41A Δ*bgaA*::P-*kan-cps23BJ***	-	0
rpsL1 CPS41A ΔCEP::P-*kan-cps7AJ-L-cps12AJ-L-cps19AJ*	0	0

"-", not tested.

[a] Diagnostic PCR demonstrated that the corresponding amplicons were not introduced in these isolates.

[b] These clones are likely suppressors because the cells did not survive after re-streaking or were unencapsulated.

Table S11. Predicted residue that is involved in complementation

Genotype of recipient cells	Transformants obtained when the indicated amplicons was introduced (CFU)	
	**None**	**Δ*cps34J*::P-*erm***
rpsL1 CPS41A P-*kan-cps34J*$^+$	-	>300
rpsL1 CPS47A P-*kan-cps34J*$^+$	-	>300
rpsL1 CPS34 P-*kan-cps34J*(T333F)	-	>300
rpsL1 CPS41A P-*kan-cps34J*(T333F)	-	>300[a] (tiny)
rpsL1 CPS47A P-*kan-cps34J*(T333F)	0	6[a]

"-", not tested.

[a] These clones are likely suppressors because the cells were unencapsulated.

Table S12. Bacterial strains in this study.

Strain	Relevant genotype	Deviation	Selectable marker	Source
IU1690	Serotype 2 strain D39W	-	None	(54)
IU1781	*rpsL1*	*rpsL1* x IU1690	Str^{R}	(55)
IU5122	*rpsL1* Δ*cps* CEP::*kan-rpsL*	-	Kan^{R}	(56)
AKF_Spn024	D39 Δ*bgaA*::*tet*-P_{Zn}::*pbp1a*	-	Tet^{R}	(57)
SpnYL001	TIGR4S Δ*cps*::*sacB-kan-rpsL*$^{+}$	-	Kan^{R}, Str^{S}, Suc^{S}	(58)
NUS0064	*rpsL1* Δ*cps2J*::P-*kan-rpsL*$^{+}$ Δ*bgaA*::P_{Zn}-*cps2J*	-	Kan^{R}, Str^{S}, Zn^{2+}	(21)
NUS0114	*rpsL1* Δ*cps*::*sacB-kan-rpsL*$^{+}$	Δ*cps*::*sacB-kan-rpsL*$^{+}$ x IU1781	Kan^{R}, Str^{S}, Suc^{S}	(29)
NUS0723	*rpsL1* Δ*cps2J<>tacF* Δ*bgaA*::P_{Zn}-*cps2J*	-	Str^{R}	(21)
HMS0019	*rpsL1* Δ*CEP*::P-*kan-rpsL*	Δ*CEP*::P-*kan-rpsL* x IU1781	Kan^{R}, Str^{S}	(30)
NUS2500	*rpsL1* CPS14 Δ*bgaA*::P_{spxB}-*cps23BJ*(P254S)	-	Str^{R}	(30)
NUS2863	*rpsL1* Δ*bgaA*::P-*kan-cps23BJ*(P254S, T38M)	Δ*bgaA*::P-*kan-cps23BJ*(P254S, T38M) x IU1781	Kan^{R}	(30)
NUS2864	*rpsL1* Δ*bgaA*::P-*kan-cps23BJ*(P254S, I246T)	Δ*bgaA*::P-*kan-cps23BJ*(P254S, I246T) x IU1781	Kan^{R}	(30)
NUS2865	*rpsL1* Δ*bgaA*::P-*kan-cps23BJ*(P254S, Q276L)	Δ*bgaA*::P-*kan-cps23BJ*(P254S, Q276L) x IU1781	Kan^{R}	(30)
NUS2866	*rpsL1* Δ*bgaA*::P-*kan-cps23BJ*(P254S, L414P)	Δ*bgaA*::P-*kan-cps23BJ*(P254S, L414P) x IU1781	Kan^{R}	(30)
NUS2247	*rpsL1* Δ*CEP::*P_{Zn}-*ytgP*	Δ*CEP::*P_{Zn}-*ytgP* x HMS0019	Str^{R}	(30)
NUS1992	*rpsL1* ΔCEP::P_{Zn}-*ytgP* Δ*ytgP*::P-*erm*	Δ*ytgP*::P-*erm* x NUS2247	Erm^{R}	(30)
NUS3248	*rpsL1* CPS33B Δ*bgaA*::P-*kan-cps23BJ*(P254S, I246T)	Δ*bgaA*::P-*kan-cps23BJ*(P254S, I246T) x NUS0308	Kan^{R}	(30)
NUS0269	*rpsL1* CPS34 (backcrossed) Δ*bgaA*::P-*kan-rpsL*$^{+}$	Δ*bgaA*::P-*kan-rpsL*$^{+}$ × NUS0265	Kan^{R}, Str^{S}	This study
NUS0304	*rpsL1* CPS34 Δ*bgaA*::P_{Zn}-*cps34J*	Δ*bgaA*::P_{Zn}-*cps34J* × NUS0269	Str^{R}	This study

NUS5030	*rpsL1* ΔCEP::P_{Zn}-*ytgP* Δ*ytgP*::P-*erm* Δ*bgaA*::P-*kan-cps23BJ*(P254S, V249A)	Δ*bgaA*::P-*kan-cps23BJ*(P254S, V249A) x NUS1992	Erm^R ,Kan^R	This study
NUS5049	*rpsL1* ΔCEP::P_{Zn}-*ytgP* Δ*ytgP*::P-*erm* Δ*bgaA*::P-*kan-cps23BJ*(P254S, G347V)	Δ*bgaA*::P-*kan-cps23BJ*(P254S, G347V) x NUS1992	Erm^R ,Kan^R	This study
NUS5410	*rpsL1 ΔCEP::Pzn-ytgP ΔytgP::p-erm ΔbgaA::P-kan-cps23BJ(P254S, L28F)*	*ΔbgaA::P-kan-cps23BJ(P254S, L28F)* x NUS1992	Erm^R ,Kan^R	This study
NUS5065	*rpsL1* ΔCEP::P_{Zn}-*ytgP* Δ*ytgP*::P-*erm* Δ*bgaA*::P-*kan-cps23BJ*(P254S)	Δ*bgaA*::P-*kan-cps23BJ*(P254S) x NUS1992	Erm^R ,Kan^R	This study
NUS5077	*rpsL1* Δ*bgaA*::P-*kan-cps23BJ*(P254S, V249A)	Δ*bgaA*::P-*kan-cps23BJ*(P254S, V249A) x IU1781	Kan^R	This study
NUS5078	*rpsL1* Δ*bgaA*::P-*kan-cps23BJ*(P254S, G347V)	Δ*bgaA*::P-*kan-cps23BJ*(P254S, G347V) x IU1781	Kan^R	This study
NUS5079	*rpsL1* Δ*bgaA*::P-*kan-cps23BJ*(P254S, L28F)	Δ*bgaA*::P-*kan-cps23BJ*(P254S, L28F) x IU1781	Kan^R	This study
NUS5080	*rpsL1* CPS23B Δ*bgaA*::P-*kan-cps23BJ*(P254S, V249A)	Δ*bgaA*::P-*kan-cps23BJ*(P254S, V249A) x NUS0329	Kan^R	This study
NUS5081	*rpsL1* CPS23B Δ*bgaA*::P-*kan-cps23BJ*(P254S, G347V)	Δ*bgaA*::P-*kan-cps23BJ*(P254S, G347V) x NUS0329	Kan^R	This study
NUS5082	*rpsL1* CPS23B Δ*bgaA*::P-*kan-cps23BJ*(P254S, L28F)	Δ*bgaA*::P-*kan-cps23BJ*(P254S, L28F) x NUS0329	Kan^R	This study
NUS5083	*rpsL1* CPS14 Δ*bgaA*::P-*kan-cps23BJ*(P254S, V249A)	Δ*bgaA*::P-*kan-cps23BJ*(P254S, V249A) x NUS0403	Kan^R	This study
NUS5084	*rpsL1* CPS14 Δ*bgaA*::P-*kan-cps23BJ*(P254S, G347V)	Δ*bgaA*::P-*kan-cps23BJ*(P254S, G347V) x NUS0403	Kan^R	This study
NUS5085	*rpsL1* CPS14 Δ*bgaA*::P-*kan-cps23BJ*(P254S, L28F)	Δ*bgaA*::P-*kan-cps23BJ*(P254S, L28F) x NUS0403	Kan^R	This study
NUS5336	*rpsL1* CPS33D Δ*cps33DE*::P-*spec-rpsL*$^+$	Δ*cps33DE*::P-*spec-rpsL*$^+$ x NUS0672	Spec^R, Str^S	This study
NUS3468	*rpsL1* CPS5 Δ*cps5E*::P-*spec-rpsL*$^+$	Δ*cps5E*::P-*spec-rpsL*$^+$ x NUS0327	Spec^R, Str^S	This study
NUS2212	*rpsL1* CPS7A Δ*cps7AE*::P-*spec-rpsL*$^+$	Δ*cps7AE*::P-*spec-rpsL*$^+$ x NUS0667	Spec^R, Str^S	This study

NUS2946	*rpsL1* CPS18B Δ*cps18BE*::P-*spec-rpsL*$^+$	Δ*cps18BE*::P-*spec-rpsL*$^+$ x NUS0330	$Spec^R$, Str^S	This study
NUS2214	*rpsL1* CPS19C Δ*cps19CE*::P-*spec-rpsL*$^+$	Δ*cps19CE*::P-*spec-rpsL*$^+$ x NUS0316	$Spec^R$, Str^S	This study
NUS2947	*rpsL1* CPS23F Δ*cps23FE*::P-*spec-rpsL*$^+$	Δ*cps23FE*::P-*spec-rpsL*$^+$ x NUS0389	$Spec^R$, Str^S	This study
NUS2919	*rpsL1* CPS27 Δ*cps27E*::P-*spec-rpsL*$^+$	Δ*cps27E*::P-*spec-rpsL*$^+$ x NUS0463	$Spec^R$, Str^S	This study
NUS5370	*rpsL1* CPS32F Δ*cps32FE*::P-*spec-rpsL*$^+$	Δ*cps32FE*::P-*spec-rpsL*$^+$ x NUS0342	$Spec^R$, Str^S	This study
NUS2948	*rpsL1* CPS33F Δ*cps33FE*::P-*spec-rpsL*$^+$	Δ*cps33FE*::P-*spec-rpsL*$^+$ x NUS0326	$Spec^R$, Str^S	This study
NUS3461	*rpsL1* CPS35C Δ*cps35CE*::P-*spec-rpsL*$^+$	Δ*cps35CE*::P-*spec-rpsL*$^+$ x NUS0369	$Spec^R$, Str^S	This study
NUS3464	*rpsL1* CPS41A Δ*cps41AE*::P-*spec-rpsL*$^+$	Δ*cps41AE*::P-*spec-rpsL*$^+$ x NUS0374	$Spec^R$, Str^S	This study
NUS3213	*rpsL1* CPS5 Δ*bgaA*::P-*kan-cps23BJ*(P254S, I246T)	Δ*bgaA*::P-*kan-cps23BJ*(P254S, I246T) x NUS0327	Kan^R	(30)
NUS3214	*rpsL1* CPS7A Δ*bgaA*::P-*kan-cps23BJ*(P254S, I246T)	Δ*bgaA*::P-*kan-cps23BJ*(P254S, I246T) x NUS0667	Kan^R	(30)
NUS3092	*rpsL1* CPS18B Δ*bgaA*::P-*kan-cps23BJ*(P254S, I246T)	Δ*bgaA*::P-*kan-cps23BJ*(P254S, I246T) x NUS0330	Kan^R	(30)
NUS3068	*rpsL1* CPS19C Δ*bgaA*::P-*kan-cps23BJ*(P254S, I246T)	Δ*bgaA*::P-*kan-cps23BJ*(P254S, I246T) x NUS0316	Kan^R	(30)
NUS3222	*rpsL1* CPS23F Δ*bgaA*::P-*kan-cps23BJ*(P254S, I246T)	Δ*bgaA*::P-*kan-cps23BJ*(P254S, I246T) x NUS0389	Kan^R	(30)
NUS3226	*rpsL1* CPS27 Δ*bgaA*::P-*kan-cps23BJ*(P254S, I246T)	Δ*bgaA*::P-*kan-cps23BJ*(P254S, I246T) x NUS0463	Kan^R	(30)
NUS3228	*rpsL1* CPS32F Δ*bgaA*::P-*kan-cps23BJ*(P254S, I246T)	Δ*bgaA*::P-*kan-cps23BJ*(P254S, I246T) x NUS0342	Kan^R	(30)
NUS3251	*rpsL1* CPS33F Δ*bgaA*::P-*kan-cps23BJ*(P254S, I246T)	Δ*bgaA*::P-*kan-cps23BJ*(P254S, I246T) x NUS0326	Kan^R	(30)
NUS3252	*rpsL1* CPS35C Δ*bgaA*::P-*kan-cps23BJ*(P254S, I246T)	Δ*bgaA*::P-*kan-cps23BJ*(P254S, I246T) x NUS0369	Kan^R	(30)
NUS3095	*rpsL1* CPS41A Δ*bgaA*::P-*kan-cps23BJ*(P254S, I246T)	Δ*bgaA*::P-*kan-cps23BJ*(P254S, I246T) x NUS0374	Kan^R	(30)
**Clinical isolates**				

PATH106	Serotype 1	-	-	CDC
NUH0001	Serotype 3	-	-	NUH
CCUG37285	Serotype 4	-	-	CCUG
PATH46	Serotype 5	-	-	CDC
NUH0002	Serotype 6A	-	-	NUH
NUH0003	Serotype 6B	-	-	NUH
NUH0004	Serotype 6C	-	-	NUH
PATH3390	Serotype 6D	-	-	CDC
NUH0005	Serotype 7F	-	-	NUH
PATH2477	Serotype 7A	-	-	CDC
PATH1803	Serotype 7B	-	-	CDC
PATH203	Serotype 7C	-	-	CDC
NUH0006	Serotype 8	-	-	NUH
PATH4969	Serotype 9A	-	-	CDC
PATH2478	Serotype 9L	-	-	CDC
PATH98	Serotype 9N	-	-	CDC
PATH30	Serotype 9V	-	-	CDC
PATH1539	Serotype 10F	-	-	CDC
PATH691	Serotype 10A	-	-	CDC
PATH2459	Serotype 10B	-	-	CDC
PATH2460	Serotype 10C	-	-	CDC
PATH122	Serotype 12F	-	-	CDC
PATH1706	Serotype 12A	-	-	CDC
PATH2479	Serotype 12B	-	-	CDC
PATH1886	Serotype 13	-	-	CDC
NUH0007	Serotype 14	-	-	NUH
NUH0011	Serotype 15F	-	-	NUH
CCUG69630	Serotype 15A	-	-	CCUG
NUH0009	Serotype 15B	-	-	NUH
NUH0010	Serotype 15C	-	-	NUH
PATH1702	Serotype 16F	-	-	CDC
PATH680	Serotype 17F	-	-	CDC
PATH2480	Serotype 17A	-	-	CDC
PATH4599	Serotype 18F	-	-	CDC
PATH4560	Serotype 18A	-	-	CDC
PATH269	Serotype 18B	-	-	CDC
NUH0012	Serotype 18C	-	-	NUH
NUH0014	Serotype 19F	-	-	NUH
NUH0013	Serotype 19A	-	-	NUH
PATH2606	Serotype 19B	-	-	CDC
PATH2463	Serotype 19C	-	-	CDC
PATH682	Serotype 20	-	-	CDC
PATH57	Serotype 21	-	-	CDC
PATH115	Serotype 22F	-	-	CDC
NUH0015	Serotype 22A	-	-	NUH
NUH0017	Serotype 23F	-	-	NUH
NUH0016	Serotype 23A	-	-	NUH

PATH212	Serotype 23B	-	-	CDC
PATH20	Serotype 24F	-	-	CDC
PATH2464	Serotype 24A	-	-	CDC
PATH2465	Serotype 24B	-	-	CDC
PATH352	Serotype 25F	-	-	CDC
PATH2466	Serotype 25A	-	-	CDC
PATH2467	Serotype 27	-	-	CDC
PATH382	Serotype 28F	-	-	CDC
PATH9002	Serotype 28A	-	-	CDC
PATH4478	Serotype 29	-	-	CDC
PATH18	Serotype 31	-	-	CDC
PATH2468	Serotype 32F	-	-	CDC
PATH6653	Serotype 32A	-	-	CDC
PATH101	Serotype 33F	-	-	CDC
PATH1754	Serotype 33A	-	-	CDC
PATH1945	Serotype 33B	-	-	CDC
PATH344	Serotype 33C	-	-	CDC
PATH2481	Serotype 33D	-	-	CDC
CCUG2399	Serotype 34	-	-	CCUG
PATH1709	Serotype 35A	-	-	CDC
PATH51	Serotype 35B	-	-	CDC
PATH1895	Serotype 35C	-	-	CDC
PATH1833	Serotype 36	-	-	CDC
PATH112	Serotype 38	-	-	CDC
PATH2009	Serotype 39	-	-	CDC
PATH2469	Serotype 40	-	-	CDC
PATH2470	Serotype 41F	-	-	CDC
PATH2471	Serotype 41A	-	-	CDC
CCUG6568	Serotype 42	-	-	CCUG
PATH2472	Serotype 43	-	-	CDC
PATH2473	Serotype 44	-	-	CDC
PATH656	Serotype 45	-	-	CDC
PATH2474	Serotype 46	-	-	CDC
PATH2475	Serotype 47F	-	-	CDC
PATH2476	Serotype 47A	-	-	CDC
PATH1937	Serotype 48	-	-	CDC
**Flippase-switched mutants**[a]				
NUS0538	*rpsL1* Δ*cps2J*<>*cps1J* Δ*bgaA*::$\mathrm{P_{Zn}}$-*cps2J*	Δ*cps2J*<>*cps1J* × NUS0064	Str^R, Zn^{2+}	(21)
NUS0900	*rpsL1* Δ*cps2J*<>*cps4J* Δ*bgaA*::$\mathrm{P_{Zn}}$-*cps2J*	Δ*cps2J*<>*cps4J* × NUS0064	Str^R, Zn^{2+}	
NUS0527	*rpsL1* Δ*cps2J*<>*cps5J* Δ*bgaA*::$\mathrm{P_{Zn}}$-*cps2J*	Δ*cps2J*<>*cps5J* × NUS0064	Str^R, Zn^{2+}	
NUS0099	*rpsL1* Δ*cps2J*<>*cps6AJ* Δ*bgaA*::$\mathrm{P_{Zn}}$-*cps2J*	Δ*cps2J*<>*cps6AJ* × NUS0064	Str^R, Zn^{2+}	

NUS0774	*rpsL1 Δcps2J<>cps6BJ ΔbgaA*::P_{Zn}-*cps2J*	*Δcps2J<>cps6BJ* × NUS0064	Str^R, Zn^{2+}	
NUS0775	*rpsL1 Δcps2J<>cps6CJ ΔbgaA*::P_{Zn}-*cps2J*	*Δcps2J<>cps6CJ* × NUS0064	Str^R, Zn^{2+}	
NUS0776	*rpsL1 Δcps2J<>cps6DJ ΔbgaA*::P_{Zn}-*cps2J*	*Δcps2J<>cps6DJ* × NUS0064	Str^R, Zn^{2+}	
NUS0539	*rpsL1 Δcps2J<>cps7AJ ΔbgaA*::P_{Zn}-*cps2J*	*Δcps2J<>cps7AJ* × NUS0064	Str^R, Zn^{2+}	
NUS0531	*rpsL1 Δcps2J<>cps7BJ ΔbgaA*::P_{Zn}-*cps2J*	*Δcps2J<>cps7BJ* × NUS0064	Str^R, Zn^{2+}	
NUS0540	*rpsL1 Δcps2J<>cps7CJ ΔbgaA*::P_{Zn}-*cps2J*	*Δcps2J<>cps7CJ* × NUS0064	Str^R, Zn^{2+}	
NUS0541	*rpsL1 Δcps2J<>cps7FJ ΔbgaA*::P_{Zn}-*cps2J*	*Δcps2J<>cps7FJ* × NUS0064	Str^R, Zn^{2+}	
NUS0095	*rpsL1 Δcps2J<>cps8J ΔbgaA*::P_{Zn}-*cps2J*	*Δcps2J<>cps8J* × NUS0064	Str^R, Zn^{2+}	
NUS0489	*rpsL1 Δcps2J<>cps9AJ ΔbgaA*::P_{Zn}-*cps2J*	*Δcps2J<>cps9AJ* × NUS0064	Str^R, Zn^{2+}	
NUS0528	*rpsL1 Δcps2J<>cps9LJ ΔbgaA*::P_{Zn}-*cps2J*	*Δcps2J<>cps9LJ* × NUS0064	Str^R, Zn^{2+}	
NUS0784	*rpsL1 Δcps2J<>cps9NJ ΔbgaA*::P_{Zn}-*cps2J*	*Δcps2J<>cps9NJ* × NUS0064	Str^R, Zn^{2+}	
NUS0783	*rpsL1 Δcps2J<>cps9VJ ΔbgaA*::P_{Zn}-*cps2J*	*Δcps2J<>cps9VJ* × NUS0064	Str^R, Zn^{2+}	
NUS0549	*rpsL1 Δcps2J<>cps10AJ ΔbgaA*::P_{Zn}-*cps2J*	*Δcps2J<>cps10AJ* × NUS0064	Str^R, Zn^{2+}	
NUS0550	*rpsL1 Δcps2J<>cps10BJ ΔbgaA*::P_{Zn}-*cps2J*	*Δcps2J<>cps10BJ* × NUS0064	Str^R, Zn^{2+}	
NUS0456	*rpsL1 Δcps2J<>cps10CJ ΔbgaA*::P_{Zn}-*cps2J*	*Δcps2J<>cps10CJ* × NUS0064	Str^R, Zn^{2+}	
NUS0455	*rpsL1 Δcps2J<>cps10FJ ΔbgaA*::P_{Zn}-*cps2J*	*Δcps2J<>cps10FJ* × NUS0064	Str^R, Zn^{2+}	
NUS0854	*rpsL1 Δcps2J<>cps12AJ ΔbgaA*::P_{Zn}-*cps2J*	*Δcps2J<>cps12AJ* × NUS0064	Str^R, Zn^{2+}	
NUS0855	*rpsL1 Δcps2J<>cps12BJ ΔbgaA*::P_{Zn}-*cps2J*	*Δcps2J<>cps12BJ* × NUS0064	Str^R, Zn^{2+}	
NUS0856	*rpsL1 Δcps2J<>cps12FJ ΔbgaA*::P_{Zn}-*cps2J*	*Δcps2J<>cps12FJ* × NUS0064	Str^R, Zn^{2+}	
NUS0457	*rpsL1 Δcps2J<>cps13J ΔbgaA*::P_{Zn}-*cps2J*	*Δcps2J<>cps13J* × NUS0064	Str^R, Zn^{2+}	
NUS0098	*rpsL1 Δcps2J<>cps14J ΔbgaA*::P_{Zn}-*cps2J*	*Δcps2J<>cps14J* × NUS0064	Str^R, Zn^{2+}	
NUS0088	*rpsL1 Δcps2J<>cps15AJ ΔbgaA*::P_{Zn}-*cps2J*	*Δcps2J<>cps15AJ* × NUS0064	Str^R, Zn^{2+}	
NUS0577	*rpsL1 Δcps2J<>cps15BJ ΔbgaA*::P_{Zn}-*cps2J*	*Δcps2J<>cps15BJ* × NUS0064	Str^R, Zn^{2+}	

NUS0580	*rpsL1* Δ*cps2J*<>*cps15CJ* Δ*bgaA*::P_{Zn}-*cps2J*	Δ*cps2J*<>*cps15CJ* × NUS0064	Str^R, Zn^{2+}	
NUS0579	*rpsL1* Δ*cps2J*<>*cps15FJ* Δ*bgaA*::P_{Zn}-*cps2J*	Δ*cps2J*<>*cps15FJ* × NUS0064	Str^R, Zn^{2+}	
NUS0877	*rpsL1* Δ*cps2J*<>*cps16FJ* Δ*bgaA*::P_{Zn}-*cps2J*	Δ*cps2J*<>*cps16FJ* × NUS0064	Str^R, Zn^{2+}	
NUS0878	*rpsL1* Δ*cps2J*<>*cps17AJ* Δ*bgaA*::P_{Zn}-*cps2J*	Δ*cps2J*<>*cps17AJ* × NUS0064	Str^R, Zn^{2+}	
NUS0879	*rpsL1* Δ*cps2J*<>*cps17FJ* Δ*bgaA*::P_{Zn}-*cps2J*	Δ*cps2J*<>*cps17FJ* × NUS0064	Str^R, Zn^{2+}	
NUS0870	*rpsL1* Δ*cps2J*<>*cps18AJ* Δ*bgaA*::P_{Zn}-*cps2J*	Δ*cps2J*<>*cps18AJ* × NUS0064	Str^R, Zn^{2+}	
NUS0866	*rpsL1* Δ*cps2J*<>*cps18BJ* Δ*bgaA*::P_{Zn}-*cps2J*	Δ*cps2J*<>*cps18BJ* × NUS0064	Str^R, Zn^{2+}	
NUS0867	*rpsL1* Δ*cps2J*<>*cps18CJ* Δ*bgaA*::P_{Zn}-*cps2J*	Δ*cps2J*<>*cps18CJ* × NUS0064	Str^R, Zn^{2+}	
NUS0868	*rpsL1* Δ*cps2J*<>*cps18FJ* Δ*bgaA*::P_{Zn}-*cps2J*	Δ*cps2J*<>*cps18FJ* × NUS0064	Str^R, Zn^{2+}	
NUS0869	*rpsL1* Δ*cps2J*<>*cps19AJ* Δ*bgaA*::P_{Zn}-*cps2J*	Δ*cps2J*<>*cps19AJ* × NUS0064	Str^R, Zn^{2+}	
NUS0874	*rpsL1* Δ*cps2J*<>*cps19BJ* Δ*bgaA*::P_{Zn}-*cps2J*	Δ*cps2J*<>*cps19BJ* × NUS0064	Str^R, Zn^{2+}	
NUS0875	*rpsL1* Δ*cps2J*<>*cps19CJ* Δ*bgaA*::P_{Zn}-*cps2J*	Δ*cps2J*<>*cps19CJ* × NUS0064	Str^R, Zn^{2+}	
NUS0871	*rpsL1* Δ*cps2J*<>*cps19FJ* Δ*bgaA*::P_{Zn}-*cps2J*	Δ*cps2J*<>*cps19FJ* × NUS0064	Str^R, Zn^{2+}	
NUS0835	*rpsL1* Δ*cps2J*<>*cps20J* Δ*bgaA*::P_{Zn}-*cps2J*	Δ*cps2J*<>*cps20J* × NUS0064	Str^R, Zn^{2+}	
NUS0836	*rpsL1* Δ*cps2J*<>*cps21J* Δ*bgaA*::P_{Zn}-*cps2J*	Δ*cps2J*<>*cps21J* × NUS0064	Str^R, Zn^{2+}	
NUS0837	*rpsL1* Δ*cps2J*<>*cps22AJ* Δ*bgaA*::P_{Zn}-*cps2J*	Δ*cps2J*<>*cps22AJ* × NUS0064	Str^R, Zn^{2+}	
NUS0838	*rpsL1* Δ*cps2J*<>*cps22FJ* Δ*bgaA*::P_{Zn}-*cps2J*	Δ*cps2J*<>*cps22FJ* × NUS0064	Str^R, Zn^{2+}	
NUS0529	*rpsL1* Δ*cps2J*<>*cps23AJ* Δ*bgaA*::P_{Zn}-*cps2J*	Δ*cps2J*<>*cps23AJ* × NUS0064	Str^R, Zn^{2+}	
NUS0141	*rpsL1* Δ*cps2J*<>*cps23BJ* Δ*bgaA*::P_{Zn}-*cps2J*	Δ*cps2J*<>*cps23BJ* × NUS0064	Str^R, Zn^{2+}	
NUS0089	*rpsL1* Δ*cps2J*<>*cps23FJ* Δ*bgaA*::P_{Zn}-*cps2J*	Δ*cps2J*<>*cps23FJ* × NUS0064	Str^R, Zn^{2+}	
NUS0886	*rpsL1* Δ*cps2J*<>*cps24AJ* Δ*bgaA*::P_{Zn}-*cps2J*	Δ*cps2J*<>*cps24AJ* × NUS0064	Str^R, Zn^{2+}	
NUS0761	*rpsL1* Δ*cps2J*<>*cps24BJ* Δ*bgaA*::P_{Zn}-*cps2J*	Δ*cps2J*<>*cps24BJ* × NUS0064	Str^R, Zn^{2+}	
NUS0762	*rpsL1* Δ*cps2J*<>*cps24FJ* Δ*bgaA*::P_{Zn}-*cps2J*	Δ*cps2J*<>*cps24FJ* × NUS0064	Str^R, Zn^{2+}	

NUS0760	*rpsL1 Δcps2J<>cps25AJ ΔbgaA*::P_{Zn}-*cps2J*	*Δcps2J<>cps25AJ* × NUS0064	Str^R, Zn^{2+}	
NUS0695	*rpsL1 Δcps2J<>cps25FJ ΔbgaA*::P_{Zn}-*cps2J*	*Δcps2J<>cps25FJ* × NUS0064	Str^R, Zn^{2+}	
NUS0839	*rpsL1 Δcps2J<>cps27J ΔbgaA*::P_{Zn}-*cps2J*	*Δcps2J<>cps27J* × NUS0064	Str^R, Zn^{2+}	
NUS0840	*rpsL1 Δcps2J<>cps28AJ ΔbgaA*::P_{Zn}-*cps2J*	*Δcps2J<>cps28AJ* × NUS0064	Str^R, Zn^{2+}	
NUS0841	*rpsL1 Δcps2J<>cps28FJ ΔbgaA*::P_{Zn}-*cps2J*	*Δcps2J<>cps28FJ* × NUS0064	Str^R, Zn^{2+}	
NUS0901	*rpsL1 Δcps2J<>cps29J ΔbgaA*::P_{Zn}-*cps2J*	*Δcps2J<>cps29J* × NUS0064	Str^R, Zn^{2+}	
NUS0641	*rpsL1 Δcps2J<>cps31J ΔbgaA*::P_{Zn}-*cps2J*	*Δcps2J<>cps31J* × NUS0064	Str^R, Zn^{2+}	
NUS0652	*rpsL1 Δcps2J<>cps32AJ ΔbgaA*::P_{Zn}-*cps2J*	*Δcps2J<>cps32AJ* × NUS0064	Str^R, Zn^{2+}	
NUS0653	*rpsL1 Δcps2J<>cps32FJ ΔbgaA*::P_{Zn}-*cps2J*	*Δcps2J<>cps32FJ* × NUS0064	Str^R, Zn^{2+}	
NUS0356	*rpsL1 Δcps2J<>cps33AJ ΔbgaA*::P_{Zn}-*cps2J*	*Δcps2J<>cps33AJ* × NUS0064	Str^R, Zn^{2+}	
NUS0266	*rpsL1 Δcps2J<>cps33BJ ΔbgaA*::P_{Zn}-*cps2J*	*Δcps2J<>cps33BJ* × NUS0064	Str^R, Zn^{2+}	
NUS0142	*rpsL1 Δcps2J<>cps33CJ ΔbgaA*::P_{Zn}-*cps2J*	*Δcps2J<>cps33CJ* × NUS0064	Str^R, Zn^{2+}	
NUS0362	*rpsL1 Δcps2J<>cps33DJ ΔbgaA*::P_{Zn}-*cps2J*	*Δcps2J<>cps33DJ* × NUS0064	Str^R, Zn^{2+}	
NUS0363	*rpsL1 Δcps2J<>cps33FJ ΔbgaA*::P_{Zn}-*cps2J*	*Δcps2J<>cps33FJ* × NUS0064	Str^R, Zn^{2+}	
NUS0096	*rpsL1 Δcps2J<>cps34J ΔbgaA*::P_{Zn}-*cps2J*	*Δcps2J<>cps34J* × NUS0064	Str^R, Zn^{2+}	
NUS0143	*rpsL1 Δcps2J<>cps35AJ ΔbgaA*::P_{Zn}-*cps2J*	*Δcps2J<>cps35AJ* × NUS0064	Str^R, Zn^{2+}	
NUS0094	*rpsL1 Δcps2J<>cps35BJ ΔbgaA*::P_{Zn}-*cps2J*	*Δcps2J<>cps35BJ* × NUS0064	Str^R, Zn^{2+}	
NUS0578	*rpsL1 Δcps2J<>cps35CJ ΔbgaA*::P_{Zn}-*cps2J*	*Δcps2J<>cps35CJ* × NUS0064	Str^R, Zn^{2+}	
NUS0581	*rpsL1 Δcps2J<>cps36J ΔbgaA*::P_{Zn}-*cps2J*	*Δcps2J<>cps36J* × NUS0064	Str^R, Zn^{2+}	
NUS0694	*rpsL1 Δcps2J<>cps38J ΔbgaA*::P_{Zn}-*cps2J*	*Δcps2J<>cps38J* × NUS0064	Str^R, Zn^{2+}	
NUS0551	*rpsL1 Δcps2J<>cps39J ΔbgaA*::P_{Zn}-*cps2J*	*Δcps2J<>cps39J* × NUS0064	Str^R, Zn^{2+}	
NUS0654	*rpsL1 Δcps2J<>cps40J ΔbgaA*::P_{Zn}-*cps2J*	*Δcps2J<>cps40J* × NUS0064	Str^R, Zn^{2+}	
NUS0642	*rpsL1 Δcps2J<>cps41AJ ΔbgaA*::P_{Zn}-*cps2J*	*Δcps2J<>cps41AJ* × NUS0064	Str^R, Zn^{2+}	

NUS0643	*rpsL1 Δcps2J<>cps41FJ ΔbgaA*::P_{Zn}-*cps2J*	*Δcps2J<>cps41FJ* × NUS0064	Str^R, Zn^{2+}	
NUS1110	*rpsL1 Δcps2J<>cps42J ΔbgaA*::P_{Zn}-*cps2J*	*Δcps2J<>cps42J* × NUS0064	Str^R, Zn^{2+}	
NUS0644	*rpsL1 Δcps2J<>cps43J ΔbgaA*::P_{Zn}-*cps2J*	*Δcps2J<>cps43J* × NUS0064	Str^R, Zn^{2+}	
NUS0655	*rpsL1 Δcps2J<>cps44J ΔbgaA*::P_{Zn}-*cps2J*	*Δcps2J<>cps44J* × NUS0064	Str^R, Zn^{2+}	
NUS0645	*rpsL1 Δcps2J<>cps45J ΔbgaA*::P_{Zn}-*cps2J*	*Δcps2J<>cps45J* × NUS0064	Str^R, Zn^{2+}	
NUS0656	*rpsL1 Δcps2J<>cps46J ΔbgaA*::P_{Zn}-*cps2J*	*Δcps2J<>cps46J* × NUS0064	Str^R, Zn^{2+}	
NUS0657	*rpsL1 Δcps2J<>cps47AJ ΔbgaA*::P_{Zn}-*cps2J*	*Δcps2J<>cps47AJ* × NUS0064	Str^R, Zn^{2+}	
NUS0658	*rpsL1 Δcps2J<>cps47FJ ΔbgaA*::P_{Zn}-*cps2J*	*Δcps2J<>cps47FJ* × NUS0064	Str^R, Zn^{2+}	
NUS0659	*rpsL1 Δcps2J<>cps48J ΔbgaA*::P_{Zn}-*cps2J*	*Δcps2J<>cps48J* × NUS0064	Str^R, Zn^{2+}	
**Backcrossed capsule switched mutants**				
NUS0352	*rpsL1* CPS2	-	Str^R	
NUS1130	*rpsL1* CPS4	-	Str^R	
NUS0327	*rpsL1* CPS5	-	Str^R	
NUS1097	*rpsL1* CPS6A	-	Str^R	
NUS0325	*rpsL1* CPS6B	-	Str^R	
NUS0278	*rpsL1* CPS6C	-	Str^R	
NUS0309	*rpsL1* CPS6D	-	Str^R	
NUS0667	*rpsL1* CPS7A	-	Str^R	
NUS0668	*rpsL1* CPS7B	-	Str^R	
NUS0279	*rpsL1* CPS7C	-	Str^R	
NUS0277	*rpsL1* CPS8	-	Str^R	
NUS0345	*rpsL1* CPS9A	-	Str^R	
NUS0343	*rpsL1* CPS9L	-	Str^R	
NUS0353	*rpsL1* CPS9N	-	Str^R	
NUS0283	*rpsL1* CPS9V	-	Str^R	
NUS0280	*rpsL1* CPS10F	-	Str^R	
NUS0312	*rpsL1* CPS10A	-	Str^R	
NUS0315	*rpsL1* CPS10C	-	Str^R	
NUS0314	*rpsL1* CPS12F	-	Str^R	
NUS0669	*rpsL1* CPS12A	-	Str^R	
NUS0295	*rpsL1* CPS12B	-	Str^R	
NUS0293	*rpsL1* CPS13	-	Str^R	
NUS0403	*rpsL1* CPS14	-	Str^R	
NUS0397	*rpsL1* CPS15F	-	Str^R	
NUS0999	*rpsL1* CPS15A	-	Str^R	
NUS0340	*rpsL1* CPS15B	-	Str^R	
NUS0289	*rpsL1* CPS15C	-	Str^R	

NUS0292	*rpsL1* CPS16F	-	Str^R	
NUS0313	*rpsL1* CPS17F	-	Str^R	
NUS0344	*rpsL1* CPS17A	-	Str^R	
NUS0465	*rpsL1* CPS18F	-	Str^R	
NUS0391	*rpsL1* CPS18A	-	Str^R	
NUS0330	*rpsL1* CPS18B	-	Str^R	
NUS0364	*rpsL1* CPS18C	-	Str^R	
NUS0276	*rpsL1* CPS19F	-	Str^R	
NUS0282	*rpsL1* CPS19A	-	Str^R	
NUS0317	*rpsL1* CPS19B	-	Str^R	
NUS0316	*rpsL1* CPS19C	-	Str^R	
NUS0331	*rpsL1* CPS20	-	Str^R	
NUS0328	*rpsL1* CPS21	-	Str^R	
NUS0670	*rpsL1* CPS22F	-	Str^R	
NUS1054	*rpsL1* CPS22A	-	Str^R	
NUS0389	*rpsL1* CPS23F	-	Str^R	
NUS0399	*rpsL1* CPS23A	-	Str^R	
NUS0329	*rpsL1* CPS23B	-	Str^R	
NUS0311	*rpsL1* CPS24F	-	Str^R	
NUS0334	*rpsL1* CPS24A	-	Str^R	
NUS0247	*rpsL1* CPS24B	-	Str^R	
NUS0463	*rpsL1* CPS27	-	Str^R	
NUS0390	*rpsL1* CPS28F	-	Str^R	
NUS0671	*rpsL1* CPS28A	-	Str^R	
NUS0354	*rpsL1* CPS29	-	Str^R	
NUS1055	*rpsL1* CPS31	-	Str^R	
NUS0342	*rpsL1* CPS32F	-	Str^R	
NUS1098	*rpsL1* CPS32A	-	Str^R	
NUS0326	*rpsL1* CPS33F	-	Str^R	
NUS0332	*rpsL1* CPS33A	-	Str^R	
NUS0308	*rpsL1* CPS33B	-	Str^R	
NUS0290	*rpsL1* CPS33C	-	Str^R	
NUS0672	*rpsL1* CPS33D	-	Str^R	
NUS0265	*rpsL1* CPS34	-	Str^R	
NUS0368	*rpsL1* CPS35A	-	Str^R	
NUS0366	*rpsL1* CPS35B	-	Str^R	
NUS0369	*rpsL1* CPS35C	-	Str^R	
NUS0400	*rpsL1* CPS36	-	Str^R	
NUS0253	*rpsL1* CPS39	-	Str^R	
NUS0372	*rpsL1* CPS40	-	Str^R	
NUS0675	*rpsL1* CPS41F	-	Str^R	
NUS0374	*rpsL1* CPS41A	-	Str^R	
NUS1079	*rpsL1* CPS42	-	Str^R	
NUS0375	*rpsL1* CPS43	-	Str^R	
NUS0718	*rpsL1* CPS44	-	Str^R	
NUS1056	*rpsL1* CPS45	-	Str^R	
NUS1057	*rpsL1* CPS46	-	Str^R	

NUS0382	*rpsL1* CPS47F	-	Str^R	
NUS1080	*rpsL1* CPS47A	-	Str^R	
**9 nucleotide molecular-barcoded, isogenic-capsule switch mutants[b]**				
NUS0589	*rpsL1* CPS1 Δ*CEP*::P-*kan-rpsL*-GCTATCCGG	Δ*CEP*::P-*kan-rpsL*-GCTATCCGG x NUS0721	Kan^R, Str^S	
NUS0590	*rpsL1* CPS2 Δ*CEP*::P-*kan-rpsL*-TCGTATAAT	Δ*CEP*::P-*kan-rpsL*-TCGTATAAT x NUS0352	Kan^R, Str^S	
NUS1160	*rpsL1* CPS4 Δ*CEP*::P-*kan-rpsL*-CAATGGGGT	Δ*CEP*::P-*kan-rpsL*-CAATGGGGT x NUS1130	Kan^R, Str^S	
NUS1345	*rpsL1* CPS5 Δ*CEP*::P-*kan-rpsL*-TAGCAAATG	Δ*CEP*::P-*kan-rpsL*-TAGCAAATG x NUS0327	Kan^R, Str^S	
NUS1157	*rpsL1* CPS6A Δ*CEP*::P-*kan-rpsL*-GCAGCCTAA	Δ*CEP*::P-*kan-rpsL*-GCAGCCTAA x NUS1097	Kan^R, Str^S	
NUS0592	*rpsL1* CPS6B Δ*CEP*::P-*kan-rpsL*-ACGATATCG	Δ*CEP*::P-*kan-rpsL*-ACGATATCG x NUS0325	Kan^R, Str^S	
NUS0994	*rpsL1* CPS6C Δ*CEP*::P-*kan-rpsL*-GGGACTTGC	Δ*CEP*::P-*kan-rpsL*-GGGACTTGC x NUS0278	Kan^R, Str^S	
NUS1074	*rpsL1* CPS6D Δ*CEP*::P-*kan-rpsL*-GATGTACAT	Δ*CEP*::P-*kan-rpsL*-GATGTACAT x NUS0309	Kan^R, Str^S	
NUS1149	*rpsL1* CPS7F Δ*CEP*::P-*kan-rpsL*-GTAGTTATC	Δ*CEP*::P-*kan-rpsL*-GTAGTTATC x NUS0310	Kan^R, Str^S	
NUS1012	*rpsL1* CPS7A Δ*CEP*::P-*kan-rpsL*-CTGTATCGG	Δ*CEP*::P-*kan-rpsL*-CTGTATCGG x NUS0667	Kan^R, Str^S	(29)
NUS1004	*rpsL1* CPS7B Δ*CEP*::P-*kan-rpsL*-AACATCAAT	Δ*CEP*::P-*kan-rpsL*-AACATCAAT x NUS0668	Kan^R, Str^S	
NUS0593	*rpsL1* CPS7C Δ*CEP*::P-*kan-rpsL*-TGTGCATCG	Δ*CEP*::P-*kan-rpsL*-TGTGCATCG x NUS0279	Kan^R, Str^S	
NUS1028	*rpsL1* CPS8 Δ*CEP*::P-*kan-rpsL*-GGCCAGCAT	Δ*CEP*::P-*kan-rpsL*-GGCCAGCAT x NUS0277	Kan^R, Str^S	
NUS0975	*rpsL1* CPS9A Δ*CEP*::P-*kan-rpsL*-AATCATGTC	Δ*CEP*::P-*kan-rpsL*-AATCATGTC x NUS0345	Kan^R, Str^S	
NUS0594	*rpsL1* CPS9L Δ*CEP*::P-*kan-rpsL*-ATCTGGGAA	Δ*CEP*::P-*kan-rpsL*-ATCTGGGAA x NUS0343	Kan^R, Str^S	
NUS0595	*rpsL1* CPS9N Δ*CEP*::P-*kan-rpsL*-GTGGCCGTA	Δ*CEP*::P-*kan-rpsL*-GTGGCCGTA x NUS0353	Kan^R, Str^S	
NUS0596	*rpsL1* CPS9V Δ*CEP*::P-*kan-rpsL*-GGATAAATA	Δ*CEP*::P-*kan-rpsL*-GGATAAATA x NUS0283	Kan^R, Str^S	
NUS0982	*rpsL1* CPS10F Δ*CEP*::P-*kan-rpsL*-ATTCAATAA	Δ*CEP*::P-*kan-rpsL*-ATTCAATAA x NUS0280	Kan^R, Str^S	
NUS0986	*rpsL1* CPS10A Δ*CEP*::P-*kan-rpsL*-AGATGTCGT	Δ*CEP*::P-*kan-rpsL*-AGATGTCGT x NUS0312	Kan^R, Str^S	

NUS2369	*rpsL1* CPS2 Δ*CEP*::P-*kan-rpsL*-TTTGCTAGT	Δ*CEP*::P-*kan-rpsL*-TTTGCTAGT x IU1781	Kan^R, Str^S	This study
NUS0597	*rpsL1* CPS10C Δ*CEP*::P-*kan-rpsL*-ATACGTCTA	Δ*CEP*::P-*kan-rpsL*-ATACGTCTA x NUS0315	Kan^R, Str^S	(29)
NUS1025	*rpsL1* CPS11F Δ*CEP*::P-*kan-rpsL*-GGCGTCACC	Δ*CEP*::P-*kan-rpsL*-GGCGTCACC x NUS0371	Kan^R, Str^S	
NUS1005	*rpsL1* CPS11B Δ*CEP*::P-*kan-rpsL*-TGTGCAGGT	Δ*CEP*::P-*kan-rpsL*-TGTGCAGGT x NUS0296	Kan^R, Str^S	
NUS0995	*rpsL1* CPS11C Δ*CEP*::P-*kan-rpsL*-GGGCCGACG	Δ*CEP*::P-*kan-rpsL*-GGGCCGACG x NUS0291	Kan^R, Str^S	
NUS0598	*rpsL1* CPS11D Δ*CEP*::P-*kan-rpsL*-CCCTGATTT	Δ*CEP*::P-*kan-rpsL*-CCCTGATTT x NUS0294	Kan^R, Str^S	
NUS0599	*rpsL1* CPS12F Δ*CEP*::P-*kan-rpsL*-TAATAGCAC	Δ*CEP*::P-*kan-rpsL*-TAATAGCAC x NUS0314	Kan^R, Str^S	
NUS1006	*rpsL1* CPS12A Δ*CEP*::P-*kan-rpsL*-ACACGGGGC	Δ*CEP*::P-*kan-rpsL*-ACACGGGGC x NUS0669	Kan^R, Str^S	
NUS0987	*rpsL1* CPS12B Δ*CEP*::P-*kan-rpsL*-AATACCAGC	Δ*CEP*::P-*kan-rpsL*-AATACCAGC x NUS0295	Kan^R, Str^S	
NUS0600	*rpsL1* CPS13 Δ*CEP*::P-*kan-rpsL*-ACAAGCTAG	Δ*CEP*::P-*kan-rpsL*-ACAAGCTAG x NUS0293	Kan^R, Str^S	
NUS0661	*rpsL1* CPS14 Δ*CEP*::P-*kan-rpsL*-TCTCGCGGG	Δ*CEP*::P-*kan-rpsL*-TCTCGCGGG x NUS0403	Kan^R, Str^S	
NUS0602	*rpsL1* CPS15F Δ*CEP*::P-*kan-rpsL*-AACGCTCGT	Δ*CEP*::P-*kan-rpsL*-AACGCTCGT x NUS0397	Kan^R, Str^S	
NUS1024	*rpsL1* CPS15A Δ*CEP*::P-*kan-rpsL*-CAGCATACG	Δ*CEP*::P-*kan-rpsL*-CAGCATACG x NUS0999	Kan^R, Str^S	
NUS0603	*rpsL1* CPS15B Δ*CEP*::P-*kan-rpsL*-GTTATAAAT	Δ*CEP*::P-*kan-rpsL*-GTTATAAAT x NUS0340	Kan^R, Str^S	
NUS0991	*rpsL1* CPS15C Δ*CEP*::P-*kan-rpsL*-CAAAGGCTC	Δ*CEP*::P-*kan-rpsL*-CAAAGGCTC x NUS0289	Kan^R, Str^S	
NUS0976	*rpsL1* CPS16F Δ*CEP*::P-*kan-rpsL*-AGAGTGCAG	Δ*CEP*::P-*kan-rpsL*-AGAGTGCAG x NUS0292	Kan^R, Str^S	
NUS0605	*rpsL1* CPS17F Δ*CEP*::P-*kan-rpsL*-CCCATCTAA	Δ*CEP*::P-*kan-rpsL*-CCCATCTAA x NUS0313	Kan^R, Str^S	
NUS0983	*rpsL1* CPS17A Δ*CEP*::P-*kan-rpsL*-CGGTACGCA	Δ*CEP*::P-*kan-rpsL*-CGGTACGCA x NUS0344	Kan^R, Str^S	
NUS0606	*rpsL1* CPS18F Δ*CEP*::P-*kan-rpsL*-TCTCGATTG	Δ*CEP*::P-*kan-rpsL*-TCTCGATTG x NUS0465	Kan^R, Str^S	

NUS0977	*rpsL1* CPS18A Δ*CEP*::P-*kan-rpsL*-CTCGGTCGC	Δ*CEP*::P-*kan-rpsL*-CTCGGTCGC x NUS0391	Kan^R, Str^S	
NUS0608	*rpsL1* CPS18B Δ*CEP*::P-*kan-rpsL*-CTTTCACAC	Δ*CEP*::P-*kan-rpsL*-CTTTCACAC x NUS0330	Kan^R, Str^S	
NUS0609	*rpsL1* CPS18C Δ*CEP*::P-*kan-rpsL*-TCCGCGAAA	Δ*CEP*::P-*kan-rpsL*-TCCGCGAAA x NUS0364	Kan^R, Str^S	
NUS0992	*rpsL1* CPS19F Δ*CEP*::P-*kan-rpsL*-ATTCATACC	Δ*CEP*::P-*kan-rpsL*-ATTCATACC x NUS0276	Kan^R, Str^S	
NUS0988	*rpsL1* CPS19A Δ*CEP*::P-*kan-rpsL*-GCTCATTCA	Δ*CEP*::P-*kan-rpsL*-GCTCATTCA x NUS0282	Kan^R, Str^S	
NUS0610	*rpsL1* CPS19B Δ*CEP*::P-*kan-rpsL*-CTAGGTTGC	Δ*CEP*::P-*kan-rpsL*-CTAGGTTGC x NUS0317	Kan^R, Str^S	
NUS0993	*rpsL1* CPS19C Δ*CEP*::P-*kan-rpsL*-GAAGCCTAC	Δ*CEP*::P-*kan-rpsL*-GAAGCCTAC x NUS0316	Kan^R, Str^S	
NUS0978	*rpsL1* CPS20 Δ*CEP*::P-*kan-rpsL*-ACTGATATA	Δ*CEP*::P-*kan-rpsL*-ACTGATATA x NUS0331	Kan^R, Str^S	
NUS0611	*rpsL1* CPS21 Δ*CEP*::P-*kan-rpsL*-GCTAGAGCA	Δ*CEP*::P-*kan-rpsL*-GCTAGAGCA x NUS0328	Kan^R, Str^S	
NUS1001	*rpsL1* CPS22F Δ*CEP*::P-*kan-rpsL*-GGGCTCTTA	Δ*CEP*::P-*kan-rpsL*-GGGCTCTTA x NUS0670	Kan^R, Str^S	
NUS1151	*rpsL1* CPS22A Δ*CEP*::P-*kan-rpsL*-AAATTCGGA	Δ*CEP*::P-*kan-rpsL*-AAATTCGGA x NUS1054	Kan^R, Str^S	
NUS0612	*rpsL1* CPS23F Δ*CEP*::P-*kan-rpsL*-GTTGTAGAT	Δ*CEP*::P-*kan-rpsL*-GTTGTAGAT x NUS0389	Kan^R, Str^S	
NUS0613	*rpsL1* CPS23A Δ*CEP*::P-*kan-rpsL*-GCTCAATAC	Δ*CEP*::P-*kan-rpsL*-GCTCAATAC x NUS0399	Kan^R, Str^S	
NUS0614	*rpsL1* CPS23B Δ*CEP*::P-*kan-rpsL*-TCCAATCGG	Δ*CEP*::P-*kan-rpsL*-TCCAATCGG x NUS0329	Kan^R, Str^S	
NUS0615	*rpsL1* CPS24F Δ*CEP*::P-*kan-rpsL*-TTTTTTCGT	Δ*CEP*::P-*kan-rpsL*-TTTTTTCGT x NUS0311	Kan^R, Str^S	
NUS0616	*rpsL1* CPS24A Δ*CEP*::P-*kan-rpsL*-CGGGTGGCT	Δ*CEP*::P-*kan-rpsL*-CGGGTGGCT x NUS0334	Kan^R, Str^S	
NUS1008	*rpsL1* CPS24B Δ*CEP*::P-*kan-rpsL*-TTGACATCG	Δ*CEP*::P-*kan-rpsL*-TTGACATCG x NUS0247	Kan^R, Str^S	
NUS0618	*rpsL1* CPS27 Δ*CEP*::P-*kan-rpsL*-GGCTGAAAG	Δ*CEP*::P-*kan-rpsL*-GGCTGAAAG x NUS0463	Kan^R, Str^S	
NUS0619	*rpsL1* CPS28F Δ*CEP*::P-*kan-rpsL*-TAGCGCCGG	Δ*CEP*::P-*kan-rpsL*-TAGCGCCGG x NUS0390	Kan^R, Str^S	
NUS1009	*rpsL1* CPS28A Δ*CEP*::P-*kan-rpsL*-GTAAGGTAC	Δ*CEP*::P-*kan-rpsL*-GTAAGGTAC x NUS0671	Kan^R, Str^S	

NUS0620	*rpsL1* CPS29 Δ*CEP*::P-*kan-rpsL*-GCGCCTGGT	Δ*CEP*::P-*kan-rpsL*-GCGCCTGGT x NUS0354	Kan^R, Str^S	
NUS1159	*rpsL1* CPS31 Δ*CEP*::P-*kan-rpsL*-ATGGCAGGA	Δ*CEP*::P-*kan-rpsL*-ATGGCAGGA x NUS1055	Kan^R, Str^S	
NUS0621	*rpsL1* CPS32F Δ*CEP*::P-*kan-rpsL*-CTATGAAGC	Δ*CEP*::P-*kan-rpsL*-CTATGAAGC x NUS0342	Kan^R, Str^S	
NUS1158	*rpsL1* CPS32A Δ*CEP*::P-*kan-rpsL*-CAATACAAA	Δ*CEP*::P-*kan-rpsL*-CAATACAAA x NUS1098	Kan^R, Str^S	
NUS0622	*rpsL1* CPS33F Δ*CEP*::P-*kan-rpsL*-GGCTACATC	Δ*CEP*::P-*kan-rpsL*-GGCTACATC x NUS0326	Kan^R, Str^S	
NUS0984	*rpsL1* CPS33A Δ*CEP*::P-*kan-rpsL*-CTCACTCGG	Δ*CEP*::P-*kan-rpsL*-CTCACTCGG x NUS0332	Kan^R, Str^S	
NUS0989	*rpsL1* CPS33B Δ*CEP*::P-*kan-rpsL*-GTGGACGGA	Δ*CEP*::P-*kan-rpsL*-GTGGACGGA x NUS0308	Kan^R, Str^S	
NUS0623	*rpsL1* CPS33C Δ*CEP*::P-*kan-rpsL*-AACGCAGAA	Δ*CEP*::P-*kan-rpsL*-AACGCAGAA x NUS0290	Kan^R, Str^S	
NUS1002	*rpsL1* CPS33D Δ*CEP*::P-*kan-rpsL*-TTATGGTTA	Δ*CEP*::P-*kan-rpsL*-TTATGGTTA x NUS0672	Kan^R, Str^S	
NUS1152	*rpsL1* CPS34 Δ*CEP*::P-*kan-rpsL*-TCCAAAAAG	Δ*CEP*::P-*kan-rpsL*-TCCAAAAAG x NUS1078	Kan^R, Str^S	
NUS0990	*rpsL1* CPS35A Δ*CEP*::P-*kan-rpsL*-CATGTCCCA	Δ*CEP*::P-*kan-rpsL*-CATGTCCCA x NUS0368	Kan^R, Str^S	
NUS1182	*rpsL1* CPS35B Δ*CEP*::P-*kan-rpsL*-GGAGTCTAT	Δ*CEP*::P-*kan-rpsL*-GGAGTCTAT x NUS0366	Kan^R, Str^S	
NUS0979	*rpsL1* CPS35C Δ*CEP*::P-*kan-rpsL*-AAAGACTTG	Δ*CEP*::P-*kan-rpsL*-AAAGACTTG x NUS0369	Kan^R, Str^S	
NUS0985	*rpsL1* CPS36 Δ*CEP*::P-*kan-rpsL*-CACCCCTAG	Δ*CEP*::P-*kan-rpsL*-CACCCCTAG x NUS0400	Kan^R, Str^S	
NUS1003	*rpsL1* CPS39 Δ*CEP*::P-*kan-rpsL*-GACGCCAGG	Δ*CEP*::P-*kan-rpsL*-GACGCCAGG x NUS0253	Kan^R, Str^S	
NUS0625	*rpsL1* CPS40 Δ*CEP*::P-*kan-rpsL*-TTGAATCCG	Δ*CEP*::P-*kan-rpsL*-TTGAATCCG x NUS0372	Kan^R, Str^S	
NUS1007	*rpsL1* CPS41F Δ*CEP*::P-*kan-rpsL*-TATTTGGAG	Δ*CEP*::P-*kan-rpsL*-TATTTGGAG x NUS0675	Kan^R, Str^S	
NUS0626	*rpsL1* CPS41A Δ*CEP*::P-*kan-rpsL*-GTACGATGG	Δ*CEP*::P-*kan-rpsL*-GTACGATGG x NUS0374	Kan^R, Str^S	
NUS1153	*rpsL1* CPS42 Δ*CEP*::P-*kan-rpsL*-ATCAGTCTG	Δ*CEP*::P-*kan-rpsL*-ATCAGTCTG x NUS1079	Kan^R, Str^S	

NUS0627	*rpsL1* CPS43 Δ*CEP*::P-*kan-rpsL*-GGTGGGACG	Δ*CEP*::P-*kan-rpsL*-GGTGGGACG x NUS0375	Kan^R, Str^S	
NUS1010	*rpsL1* CPS44 Δ*CEP*::P-*kan-rpsL*-TGCTCCATT	Δ*CEP*::P-*kan-rpsL*-TGCTCCATT x NUS0718	Kan^R, Str^S	
NUS1155	*rpsL1* CPS45 Δ*CEP*::P-*kan-rpsL*-TATACCCTG	Δ*CEP*::P-*kan-rpsL*-TATACCCTG x NUS1056	Kan^R, Str^S	
NUS1156	*rpsL1* CPS46 Δ*CEP*::P-*kan-rpsL*-CGCAGGCTG	Δ*CEP*::P-*kan-rpsL*-CGCAGGCTG x NUS1057	Kan^R, Str^S	
NUS0628	*rpsL1* CPS47F Δ*CEP*::P-*kan-rpsL*-AACCATCGT	Δ*CEP*::P-*kan-rpsL*-AACCATCGT x NUS0382	Kan^R, Str^S	
NUS1154	*rpsL1* CPS47A Δ*CEP*::P-*kan-rpsL*-CCGCAAGAT	Δ*CEP*::P-*kan-rpsL*-CCGCAAGAT x NUS1080	Kan^R, Str^S	
NUS2396	*rpsL1* CPS2 Δ*CEP*::P-*kan-rpsL*-GACCGAGGA	Δ*CEP*::P-*kan-rpsL*-GACCGAGGA x IU1781	Kan^R, Str^S	This study
**9 nucleotide molecular-barcoded flippase cassettes**				
NUS2316	*rpsL1* Δ*CEP*::P-*kan-cps1J*-GCTATCCGG	Δ*CEP*::P-*kan-cps1J*-GCTATCCGG x IU1781	Kan^R	This study
NUS1822	*rpsL1* Δ*CEP*::P-*kan-cps2J*-TCGTATAAT	Δ*CEP*::P-*kan-cps2J*-TCGTATAAT x IU1781	Kan^R	This study
NUS2317	*rpsL1* Δ*CEP*::P-*kan-cps4J*-CAATGGGGT	Δ*CEP*::P-*kan-cps4J*-CAATGGGGT x IU1781	Kan^R	This study
NUS2318	*rpsL1* Δ*CEP*::P-*kan-cps5J*-TAGCAAATG	Δ*CEP*::P-*kan-cps5J*-TAGCAAATG x IU1781	Kan^R	This study
NUS0756	*rpsL1* Δ*CEP*::P-*kan-cps6AJ*-GCAGCCTAA	Δ*CEP*::P-*kan-cps6AJ*-GCAGCCTAA x IU1781	Kan^R	This study
NUS0765	*rpsL1* Δ*CEP*::P-*kan-cps6BJ*-ACGATATCG	Δ*CEP*::P-*kan-cps6BJ*-ACGATATCGx IU1781	Kan^R	This study
NUS0781	*rpsL1* Δ*CEP*::P-*kan-cps6CJ*-GGGACTTGC	Δ*CEP*::P-*kan-cps6CJ*-GGGACTTGC x IU1781	Kan^R	This study
NUS0793	*rpsL1* Δ*CEP*::P-*kan-cps6DJ*-GATGTACAT	Δ*CEP*::P-*kan-cps6DJ*-GATGTACAT x IU1781	Kan^R	This study
NUS1051	*rpsL1* Δ*CEP*::P-*kan-cps7AJ*-CTGTATCGG	Δ*CEP*::P-*kan-cps7AJ*-CTGTATCGG x IU1781	Kan^R	This study
NUS1061	*rpsL1* Δ*CEP*::P-*kan-cps7BJ*-AACATCAAT	Δ*CEP*::P-*kan-cps7BJ*-AACATCAAT x IU1781	Kan^R	This study
NUS1107	*rpsL1* Δ*CEP*::P-*kan-cps7CJ*- TGTGCATCG	Δ*CEP*::P-*kan-cps7CJ*-TGTGCATCG x IU1781	Kan^R	This study
NUS1117	*rpsL1* Δ*CEP*::P-*kan-cps7FJ*-GTAGTTATC	Δ*CEP*::P-*kan-cps7FJ*-GTAGTTATC x IU1781	Kan^R	This study
NUS2937	*rpsL1* Δ*CEP*::P-*kan-cps8J*-TCTGGAGCG	Δ*CEP*::P-*kan-cps8J*-TCTGGAGCG x IU1781	Kan^R	This study
NUS2006	*rpsL1* Δ*CEP*::P-*kan-cps9AJ*-AATCATGTC	Δ*CEP*::P-*kan-cps9AJ*-AATCATGTC x IU1781	Kan^R	This study

NUS1994	*rpsL1* Δ*CEP*::P-*kan-cps9LJ*-ATCTGGGAA	Δ*CEP*::P-*kan-cps9LJ*-ATCTGGGAA x IU1781	Kan^R	This study
NUS1624	*rpsL1* Δ*CEP*::P-*kan-cps9NJ*-GTGGCCGTA	Δ*CEP*::P-*kan-cps9NJ*-GTGGCCGTA x IU1781	Kan^R	This study
NUS1458	*rpsL1* Δ*CEP*::P-*kan-cps9VJ*-GGATAAATA	Δ*CEP*::P-*kan-cps9VJ*-GGATAAATA x IU1781	Kan^R	This study
NUS1449	*rpsL1* Δ*CEP*::P-*kan-cps10AJ*-AGATGTCGT	Δ*CEP*::P-*kan-cps10AJ*-AGATGTCGT x IU1781	Kan^R	This study
NUS2394	*rpsL1* Δ*CEP*::P-*kan-cps10BJ*-TTTGCTAGT	Δ*CEP*::P-*kan-cps10BJ*-TTTGCTAGT x IU1781	Kan^R	This study
NUS1288	*rpsL1* Δ*CEP*::P-*kan-cps10CJ*-ATACGTCTA	Δ*CEP*::P-*kan-cps10CJ*-ATACGTCTA x IU1781	Kan^R	This study
NUS1256	*rpsL1* Δ*CEP*::P-*kan-cps10FJ*-ATTCAATAA	Δ*CEP*::P-*kan-cps10FJ*-ATTCAATAA x IU1781	Kan^R	This study
NUS2286	*rpsL1* Δ*CEP*::P-*kan-cps12AJ*-ACACGGGGC	Δ*CEP*::P-*kan-cps12AJ*-ACACGGGGC x IU1781	Kan^R	This study
NUS2287	*rpsL1* Δ*CEP*::P-*kan-cps12BJ*-TAATAGCAC	Δ*CEP*::P-*kan-cps12BJ*-TAATAGCAC x IU1781	Kan^R	This study
NUS2288	*rpsL1* Δ*CEP*::P-*kan-cps12FJ*-AATACCAGC	Δ*CEP*::P-*kan-cps12FJ*-AATACCAGC x IU1781	Kan^R	This study
NUS2289	*rpsL1* Δ*CEP*::P-*kan-cps13J*-ACAAGCTAG	Δ*CEP*::P-*kan-cps13J*-ACAAGCTAG x IU1781	Kan^R	This study
NUS2290	*rpsL1* Δ*CEP*::P-*kan-cps14J*-TCTCGCGGG	Δ*CEP*::P-*kan-cps14J*-TCTCGCGGG x IU1781	Kan^R	This study
NUS2291	*rpsL1* Δ*CEP*::P-*kan-cps15AJ*-CAGCATACG	Δ*CEP*::P-*kan-cps15AJ*-CAGCATACG x IU1781	Kan^R	This study
NUS2292	*rpsL1* Δ*CEP*::P-*kan-cps15BJ*-GTTATAAAT	Δ*CEP*::P-*kan-cps15BJ*-GTTATAAAT x IU1781	Kan^R	This study
NUS2293	*rpsL1* Δ*CEP*::P-*kan-cps15CJ*-CAAAGGCTC	Δ*CEP*::P-*kan-cps15CJ*-CAAAGGCTC x IU1781	Kan^R	This study
NUS2294	*rpsL1* Δ*CEP*::P-*kan-cps15FJ*-AACGCTCGT	Δ*CEP*::P-*kan-cps15FJ*-AACGCTCGT x IU1781	Kan^R	This study
NUS2295	*rpsL1* Δ*CEP*::P-*kan-cps16FJ*-AGAGTGCAG	Δ*CEP*::P-*kan-cps16FJ*-AGAGTGCAG x IU1781	Kan^R	This study
NUS2319	*rpsL1* Δ*CEP*::P-*kan-cps17AJ*-CGGTACGCA	Δ*CEP*::P-*kan-cps17AJ*-CGGTACGCA x IU1781	Kan^R	This study
NUS2320	*rpsL1* Δ*CEP*::P-*kan-cps17FJ*-CCCATCTAA	Δ*CEP*::P-*kan-cps17FJ*-CCCATCTAA x IU1781	Kan^R	This study
NUS2321	*rpsL1* Δ*CEP*::P-*kan-cps18AJ*-CTCGGTCGC	Δ*CEP*::P-*kan-cps18AJ*-CTCGGTCGC x IU1781	Kan^R	This study
NUS2322	*rpsL1* Δ*CEP*::P-*kan-cps18BJ*-CTTTCACAC	Δ*CEP*::P-*kan-cps18BJ*-CTTTCACAC x IU1781	Kan^R	This study
NUS2323	*rpsL1* Δ*CEP*::P-*kan-cps18CJ*-TCCGCGAAA	Δ*CEP*::P-*kan-cps18CJ*-TCCGCGAAA x IU1781	Kan^R	This study
NUS2324	*rpsL1* Δ*CEP*::P-*kan-cps18FJ*-TCTCGATTG	Δ*CEP*::P-*kan-cps18FJ*-TCTCGATTG x IU1781	Kan^R	This study
NUS2332b	*rpsL1* Δ*CEP*::P-*kan-cps19AJ*-GCTCATTCA	Δ*CEP*::P-*kan-cps19AJ*-GCTCATTCA x IU1781	Kan^R	This study

NUS2333b	*rpsL1* Δ*CEP*::P-*kan-cps19BJ*-CTAGGTTGC	Δ*CEP*::P-*kan-cps19BJ*-CTAGGTTGC x IU1781	Kan^R	This study
NUS2334b	*rpsL1* Δ*CEP*::P-*kan-cps19CJ*-GAAGCCTAC	Δ*CEP*::P-*kan-cps19CJ*-GAAGCCTAC x IU1781	Kan^R	This study
NUS2335b	*rpsL1* Δ*CEP*::P-*kan-cps19FJ*-ATTCATACC	Δ*CEP*::P-*kan-cps19FJ*-ATTCATACC x IU1781	Kan^R	This study
NUS2336	*rpsL1* Δ*CEP*::P-*kan-cps20J*-ACTGATATA	Δ*CEP*::P-*kan-cps20J*-ACTGATATA x IU1781	Kan^R	This study
NUS2337	*rpsL1* Δ*CEP*::P-*kan-cps21J*-GCTAGAGCA	Δ*CEP*::P-*kan-cps21J*-GCTAGAGCA x IU1781	Kan^R	This study
NUS2338	*rpsL1* Δ*CEP*::P-*kan-cps22AJ*-AAATTCGGA	Δ*CEP*::P-*kan-cps22AJ*-AAATTCGGA x IU1781	Kan^R	This study
NUS2339	*rpsL1* Δ*CEP*::P-*kan-cps22FJ*-GGGCTCTTA	Δ*CEP*::P-*kan-cps22FJ*-GGGCTCTTA x IU1781	Kan^R	This study
NUS2276	*rpsL1* Δ*CEP*::P-*kan-cps23AJ*-GCTCAATAC	Δ*CEP*::P-*kan-cps23AJ*-GCTCAATAC x IU1781	Kan^R	This study
NUS2009	*rpsL1* Δ*CEP*::P-*kan-cps23BJ*-TCCAATCGG	Δ*CEP*::P-*kan-cps23BJ*-TCCAATCGG x IU1781	Kan^R	This study
NUS2277	*rpsL1* Δ*CEP*::P-*kan-cps23FJ*-GTTGTAGAT	Δ*CEP*::P-*kan-cps23FJ*-GTTGTAGAT x IU1781	Kan^R	This study
NUS2278	*rpsL1* Δ*CEP*::P-*kan-cps24AJ*-CGGGTGGCT	Δ*CEP*::P-*kan-cps24AJ*-CGGGTGGCT x IU1781	Kan^R	This study
NUS2279	*rpsL1* Δ*CEP*::P-*kan-cps24BJ*-TTGACATCG	Δ*CEP*::P-*kan-cps24BJ*-TTGACATCG x IU1781	Kan^R	This study
NUS2280	*rpsL1* Δ*CEP*::P-*kan-cps24FJ*-TTTTTTCGT	Δ*CEP*::P-*kan-cps24FJ*-TTTTTTCGT x IU1781	Kan^R	This study
NUS2282	*rpsL1* Δ*CEP*::P-*kan-cps27J*-GGCTGAAAG	Δ*CEP*::P-*kan-cps27J*-GGCTGAAAG x IU1781	Kan^R	This study
NUS2283	*rpsL1* Δ*CEP*::P-*kan-cps28AJ*-GTAAGGTAC	Δ*CEP*::P-*kan-cps28AJ*-GTAAGGTAC x IU1781	Kan^R	This study
NUS2284	*rpsL1* Δ*CEP*::P-*kan-cps28FJ*-TAGCGCCGG	Δ*CEP*::P-*kan-cps28FJ*-TAGCGCCGG x IU1781	Kan^R	This study
NUS2285	*rpsL1* Δ*CEP*::P-*kan-cps29J*-GCGCCTGGT	Δ*CEP*::P-*kan-cps29J*-GCGCCTGGT x IU1781	Kan^R	This study
NUS2264	*rpsL1* Δ*CEP*::P-*kan-cps31J*-ATGGCAGGA	Δ*CEP*::P-*kan-cps31J*-ATGGCAGGA x IU1781	Kan^R	This study
NUS2265	*rpsL1* Δ*CEP*::P-*kan-cps32AJ*-CAATACAAA	Δ*CEP*::P-*kan-cps32AJ*-CAATACAAA x IU1781	Kan^R	This study
NUS2266	*rpsL1* Δ*CEP*::P-*kan-cps32FJ*-CTATGAAGC	Δ*CEP*::P-*kan-cps32FJ*-CTATGAAGC x IU1781	Kan^R	This study
NUS2267	*rpsL1* Δ*CEP*::P-*kan-cps33AJ*-CTCACTCGG	Δ*CEP*::P-*kan-cps33AJ*-CTCACTCGG x IU1781	Kan^R	This study
NUS2011	*rpsL1* Δ*CEP*::P-*kan-cps33BJ*-GTGGACGGA	Δ*CEP*::P-*kan-cps33BJ*-GTGGACGGA x IU1781	Kan^R	This study
NUS2012	*rpsL1* Δ*CEP*::P-*kan-cps33CJ*-AACGCAGAA	Δ*CEP*::P-*kan-cps33CJ*-AACGCAGAA x IU1781	Kan^R	This study
NUS2268	*rpsL1* Δ*CEP*::P-*kan-cps33DJ*-TTATGGTTA	Δ*CEP*::P-*kan-cps33DJ*-TTATGGTTA x IU1781	Kan^R	This study

NUS2269	*rpsL1* Δ*CEP*::P-*kan-cps33FJ*-GGCTACATC	Δ*CEP*::P-*kan-cps33FJ*-GGCTACATC x IU1781	Kan^R	This study
NUS2340	*rpsL1* Δ*CEP*::P-*kan-cps34J*-TCCAAAAAG	Δ*CEP*::P-*kan-cps34J*-TCCAAAAAG x IU1781	Kan^R	This study
NUS2270	*rpsL1* Δ*CEP*::P-*kan-cps35AJ*-CATGTCCCA	Δ*CEP*::P-*kan-cps35AJ*-CATGTCCCA x IU1781	Kan^R	This study
NUS2271	*rpsL1* Δ*CEP*::P-*kan-cps35BJ*-GGAGTCTAT	Δ*CEP*::P-*kan-cps35BJ*-GGAGTCTAT x IU1781	Kan^R	This study
NUS2272	*rpsL1* Δ*CEP*::P-*kan-cps35CJ*-AAAGACTTG	Δ*CEP*::P-*kan-cps35CJ*-AAAGACTTG x IU1781	Kan^R	This study
NUS2273	*rpsL1* Δ*CEP*::P-*kan-cps36J*-CACCCCTAG	Δ*CEP*::P-*kan-cps36J*-CACCCCTAG x IU1781	Kan^R	This study
NUS2252	*rpsL1* Δ*CEP*::P-*kan-cps39J*-GACGCCAGG	Δ*CEP*::P-*kan- cps39J*-GACGCCAGG x IU1781	Kan^R	This study
NUS2253	*rpsL1* Δ*CEP*::P-*kan-cps40J*-TTGAATCCG	Δ*CEP*::P-*kan-cps40J*-TTGAATCCG x IU1781	Kan^R	This study
NUS2254	*rpsL1* Δ*CEP*::P-*kan-cps41AJ*-GTACGATGG	Δ*CEP*::P-*kan-cps41AJ*-GTACGATGG x IU1781	Kan^R	This study
NUS2255	*rpsL1* Δ*CEP*::P-*kan-cps41FJ*-TATTTGGAG	Δ*CEP*::P-*kan-cps41FJ*-TATTTGGAG x IU1781	Kan^R	This study
NUS2256	*rpsL1* Δ*CEP*::P-*kan-cps42J*-ATCAGTCTG	Δ*CEP*::P-*kan-cps42J*-ATCAGTCTG x IU1781	Kan^R	This study
NUS2257	*rpsL1* Δ*CEP*::P-*kan-cps43J*-GGTGGGACG	Δ*CEP*::P-*kan-cps43J*-GGTGGGACG x IU1781	Kan^R	This study
NUS2258	*rpsL1* Δ*CEP*::P-*kan-cps44J*-TGCTCCATT	Δ*CEP*::P-*kan-cps44J*-TGCTCCATT x IU1781	Kan^R	This study
NUS2259	*rpsL1* Δ*CEP*::P-*kan-cps45J*-TATACCCTG	Δ*CEP*::P-*kan-cps45J*-TATACCCTG x IU1781	Kan^R	This study
NUS2260	*rpsL1* Δ*CEP*::P-*kan-cps46J*-CGCAGGCTG	Δ*CEP*::P-*kan-cps46J*-CGCAGGCTG x IU1781	Kan^R	This study
NUS2261	*rpsL1* Δ*CEP*::P-*kan-cps47AJ*-CCGCAAGAT	Δ*CEP*::P-*kan-cps47AJ*-CCGCAAGAT x IU1781	Kan^R	This study
NUS2262	*rpsL1* Δ*CEP*::P-*kan-cps47FJ*-AACCATCGT	Δ*CEP*::P-*kan-cps47FJ*-AACCATCGT x IU1781	Kan^R	This study
NUS2263	*rpsL1* Δ*CEP*::P-*kan-cps48J*-GACCGAGGA	Δ*CEP*::P-*kan-cps48J*-GACCGAGGA x IU1781	Kan^R	This study
NUS2910	*rpsL1* Δ*CEP*::P-*kan-cps23BJ(P254S, T38M)*-GGCGTCACC	Δ*CEP*::P-*kan-cps23BJ(P254S, T38M)*-GGCGTCACC x IU1781	Kan^R	This study
NUS2911	*rpsL1* Δ*CEP*::P-*kan-cps23BJ(P254S, I246T)*-TGTGCAGGT	Δ*CEP*::P-*kan-cps23BJ(P254S, I246T)*-TGTGCAGGT x IU1781	Kan^R	This study
NUS2912	*rpsL1* Δ*CEP*::P-*kan-cps23BJ(P254S, Q276L)*-GGGCCGACG	Δ*CEP*::P-*kan-cps23BJ(P254S, Q276L)*-GGGCCGACG x IU1781	Kan^R	This study
NUS2913	*rpsL1* Δ*CEP*::P-*kan-cps23BJ(P254S, L414P)*-CCCTGATTT	Δ*CEP*::P-*kan-cps23BJ(P254S, L414P)*-CCCTGATTT x IU1781	Kan^R	This study

Barcoded strains				
NUS3801	*rpsL1* CPS33A ΔCEP::P-*kan-cps23BJ*(*P254S, I246T*)-barcode	ΔCEP::P-*kan-cps23BJ*(*P254S, I246T*)-barcode x NUS0332	Kan^R	This study
NUS3802	*rpsL1* CPS33F ΔCEP::P-*kan-cps23BJ*(*P254S, I246T*)-barcode	ΔCEP::P-*kan-cps23BJ*(*P254S, I246T*)-barcode x NUS0326	Kan^R	This study
NUS3903	*rpsL1* CPS33A Δ*cps33AJ*::P-*erm* ΔCEP::P-*kan-cps23BJ*(*P254S, I246T*)-barcode	Δ*cps33AJ*::P-*erm* x NUS3801	Erm^R, Kan^R	This study
NUS3904	*rpsL1* CPS33F Δ*cps33FJ*::P-*erm* ΔCEP::P-*kan-cps23BJ*(*P254S, I246T*)-barcode	Δ*cps33FJ*::P-*erm* x NUS3802	Erm^R, Kan^R	This study
NUS4017	*rpsL1* CPS23B Δ*bgaA*::P-*kan-cps23BJ*(*P254S, I246T*)-barcode	Δ*bgaA*::P-*kan-cps23BJ*(*P254S, I246T*)-barcode x NUS0329	Kan^R	This study
NUS4071	*rpsL1* CPS19B ΔCEP::P-*kan-cps33AJ*-barcode	ΔCEP::P-*kan-cps33AJ*-barcode x NUS0317	Kan^R	This study
NUS4072	*rpsL1* CPS19B ΔCEP::P-*kan-cps43J*-barcode	CPS19B ΔCEP::P-*kan-cps43J*-barcode x NUS0317	Kan^R	This study
NUS4074	*rpsL1* CPS19C ΔCEP::P-*kan-cps33AJ*-barcode	ΔCEP::P-*kan-cps33AJ*-barcode x NUS0316	Kan^R	This study
NUS4075	*rpsL1* CPS19C ΔCEP::P-*kan-cps43J*-barcode	ΔCEP::P-*kan-cps43J*-barcode x NUS0316	Kan^R	This study
NUS4097	*rpsL1* CPS18C ΔCEP::P-*kan-cps18CJ*-barcode	ΔCEP::P-*kan-cps18CJ*-barcode x NUS0364	Kan^R	This study
NUS4098	*rpsL1* CPS18C ΔCEP::P-*kan-cps15BJ*-barcode	ΔCEP::P-*kan-cps15BJ*-barcode x NUS0364	Kan^R	This study
NUS4099	*rpsL1* CPS18C ΔCEP::P-*kan-cps15FJ*-barcode	ΔCEP::P-*kan-cps15FJ*-barcode x NUS0364	Kan^R	This study
NUS4100	*rpsL1* CPS18F ΔCEP::P-*kan-cps15BJ*-barcode	ΔCEP::P-*kan-cps15BJ*-barcode x NUS0465	Kan^R	This study
NUS4101	*rpsL1* CPS18F ΔCEP::P-*kan-cps15FJ*-barcode	ΔCEP::P-*kan-cps15FJ*-barcode x NUS0465	Kan^R	This study
NUS4149	*rpsL1* CPS19C Δ*cps19C*(*wchU*)::P-*spec-rpsL*$^+$	Δ*cps19C*(*wchU*)::P-*spec-rpsL*$^+$ x NUS0316	$Spec^R$	This study
NUS4154	*rpsL1* CPS19C Δ*cps19C*(*wchU*)'	Δ*cps19C*(*wchU*)' x NUS4149	Str^R	This study
NUS4188	*rpsL1* CPS19C Δ*cps19C*(*wchU*)' ΔCEP::P-*kan-cps33AJ-barcode*	ΔCEP::P-*kan-cps33AJ* x NUS4154	Kan^R	This study

NUS4189	*rpsL1* CPS19C Δ*cps19C*(*wchU*)' ΔCEP::P-*kan-cps43J-barcode*	ΔCEP::P-*kan-cps43J* x NUS4154	Kan^R	This study
NUS4190	*rpsL1* CPS28A Δ*bgaA*::P-*kan-rpsL*$^+$	Δ*bgaA*::P-*kan-rpsL*$^+$ x NUS0671	Kan^R	This study
NUS4201	*rpsL1* CPS19C Δ*cps19C*(*wchU*)' ΔCEP::P-*kan-cps33AJ-barcode* Δ*cps19CJ*::P-*erm*	Δ*cps19CJ*::P-*erm* x NUS4188	Erm^R, Kan^R	This study
NUS4202	*rpsL1* CPS19C Δ*cps19C*(*wchU*)' ΔCEP::P-*kan-cps43J-barcode* Δ*cps19CJ*::P-*erm*	Δ*cps19CJ*::P-*erm* x NUS4189	Erm^R, Kan^R	This study
NUS4203	*rpsL1* CPS18C ΔCEP::P-*kan-cps15BJ*-barcode	ΔCEP::P-*kan-cps15BJ*-barcode x NUS0364	Kan^R	This study
NUS4204	*rpsL1* CPS18C ΔCEP::P-*kan-cps15FJ*-barcode	ΔCEP::P-*kan-cps15FJ*-barcode x NUS0364	Kan^R	This study
NUS4205	*rpsL1* CPS18F ΔCEP::P-*kan-cps15BJ*-barcode	ΔCEP::P-*kan-cps15BJ*-barcode x NUS0465	Kan^R	This study
NUS4206	*rpsL1* CPS18F ΔCEP::P-*kan-cps15FJ*-barcode	ΔCEP::P-*kan-cps15FJ*-barcode x NUS0465	Kan^R	This study
NUS4207	*rpsL1* CPS28A Δ*bgaA*::P_{Zn}-*cps34J*	Δ*bgaA*::P_{Zn}-*cps34J* x NUS4190	Str^R	This study
NUS4275	*rpsL1* CPS18C ΔCEP::P-*kan-cps15BJ*-barcode Δ*cps18CJ*::P-*erm*	Δ*cps18CJ*::P-*erm* x NUS4203	Kan^R, Erm^R	This study
NUS4276	*rpsL1* CPS18C ΔCEP::P-*kan-cps15FJ*-barcode Δ*cps18CJ*::P-*erm*	Δ*cps18CJ*::P-*erm* x NUS4204	Kan^R, Erm^R	This study
NUS4277	*rpsL1* CPS18F ΔCEP::P-*kan-cps15FJ*-barcode Δ*cps18FJ*::P-*erm*	Δ*cps18FJ*::P-*erm* x NUS4205	Kan^R, Erm^R	This study
NUS4285	*rpsL1* CPS28A Δ*bgaA*::P_{Zn}-*cps28AJ*	Δ*bgaA*::P_{Zn}-*cps28AJ* x NUS4190	Str^R	This study
NUS4288	*rpsL1* CPS28A Δ*cps28AE*::P-*erm*	Δ*cps28AE*::P-*erm* x NUS0671	Erm^R	This study
NUS4545	*rpsL1* CPS28A Δ*cps28AE*::P-*erm* Δ*bgaA*-P-*sacB-kan-rpsL*$^+$	Δ*bgaA*::P-*sacB-kan-rpsL*$^+$ x NUS4288	Kan^R, Str^S, Suc^S	This study
NUS4571	*rpsL1* CPS28A Δ*cps28AE*::P-*erm* Δ*bgaA*::P_{Zn}-*cps28AE*	Δ*bgaA*::P_{Zn}-*cps28AE* x NUS4545	Str^R	This study
NUS4599	*rpsL1* CPS28A Δ*cps28AE*::P-*erm* Δ*bgaA*::P_{Zn}-*cps28AE* ΔCEP::P-*kan-cps28AJ*	ΔCEP::P-*kan-cps28AJ* x NUS4571	Kan^R, Erm^R	This study

NUS4600	*rpsL1* CPS28A Δ*cps28AE*::P-*erm* Δ*bgaA*::P$_{Zn}$-*cps28AE* ΔCEP::P-*kan-cps34J*	ΔCEP::P-*kan-cps34J* x NUS4571	Kan^R, Erm^R	This study
NUS2351	*rpsL1* CPS14 ΔCEP::P-*kan-cps14J*-barcode	ΔCEP::P-*kan-cps14J*-barcode x NUS0403	Kan^R	This study
NUS1872	*rpsL1* CPS14 Δ*bgaA*::P-*sacB-kan-rpsL*$^+$	Δ*bgaA*::P-*sacB-kan-rpsL*$^+$ x NUS0403	Kan^R, Str^S, Suc^S	(30)
NUS3038	*rpsL1* CPS14 Δ*cps14E*::P-*sacB-kan-rpsL*$^+$	Δ*cps14E*::P-*sacB-kan-rpsL*$^+$ x NUS0403	Kan^R, Str^S, Suc^S	This study
NUS3278	*rpsL1* CPS14 Δ*cps14E'*	Δ*cps14E'* x NUS3038	Str^S	This study
NUS3315	*rpsL1* CPS14 Δ*cps14E'* Δ*bgaA*::P-*spec-rpsL*$^+$	Δ*bgaA*::P-*spec-rpsL*$^+$ x NUS3278	$Spec^R$, Str^S	This study
NUS3334	*rpsL1* CPS14 Δ*cps14E'* Δ*bgaA*::P$_{Zn}$-*cps14E*	Δ*bgaA*::P$_{Zn}$-*cps14E* x NUS3315	Str^S	This study
NUS4643	*rpsL1* CPS14 Δ*bgaA*::P$_{Zn}$-*cps14J*	Δ*bgaA*::P$_{Zn}$-*cps14J* x NUS1872	Str^S	This study
NUS4644	*rpsL1* CPS14 Δ*bgaA*::P$_{Zn}$-*cps34J*	Δ*bgaA*::P$_{Zn}$-*cps34J* x NUS1872	Str^S	This study
NUS4840	*rpsL1* CPS14 Δ*bgaA*::P$_{Zn}$-*cps14J* Δ*cps14J*::P-*spec-rpsL*$^+$	Δ*cps14J*::P-*spec-rpsL*$^+$ x NUS4643	$Spec^R$, Str^S	This study
NUS4841	*rpsL1* CPS28A Δ*bgaA*::P$_{Zn}$-*cps28AJ* Δ*cps28AJ*::P-*spec-rpsL*$^+$	Δ*cps28AJ*::P-*spec-rpsL*$^+$ x NUS4285	$Spec^R$, Str^S	This study
NUS4842	*rpsL1* CPS34 Δ*bgaA*::P$_{Zn}$-*cps34J* Δ*cps34J*::P-*spec-rpsL*$^+$	Δ*cps34J*::P-*spec-rpsL*$^+$ x NUS0304	$Spec^R$, Str^S	This study
NUS4910	*rpsL1* CPS14 Δ*cps14E'* Δ*bgaA*::P$_{Zn}$-*cps14E* ΔCEP::P-*kan-cps14J*	ΔCEP::P-*kan-cps14J* x NUS3334	Kan^R	This study
NUS4911	*rpsL1* CPS14 Δ*cps14E'* Δ*bgaA*::P$_{Zn}$-*cps14E* ΔCEP::P-kan-cps34J	ΔCEP::P-*kan-cps34J* x NUS3334	Kan^R	This study
NUS5047	*rpsL1* CPS14 Δ*cps14E*::P-*erm* Δ*bgaA*::P$_{Zn}$-*cps34J*	Δ*cps14E*::P-*erm* x NUS4644	Erm^R	This study
NUS5048	*rpsL1* CPS28A Δ*cps28AE*::P-*erm* Δ*bgaA*::P$_{Zn}$-*cps34J*	Δ*cps28AE*::P-*erm* x NUS4207	Erm^R	This study
NUS5883	*rpsL1* CPS19C ΔCEP::P-*kan-cps20J*	ΔCEP::P-*kan-cps20J* x NUS0316	Kan^R	This study
NUS5884	*rpsL1* CPS19B ΔCEP::P-*kan-cps20J*	ΔCEP::P-*kan-cps20J* x NUS0317	Kan^R	This study
NUS5899	*rpsL1* CPS41A ΔCEP::P-*kan-cps34J(T333F)*	ΔCEP::P-*kan-cps34J(T333F)* x NUS0374	Kan^R	This study

NUS5900	*rpsL1* CPS47A ΔCEP::P-*kan-cps34J(T333F)*	ΔCEP::P-*kan-cps34J(T333F)* x NUS1080	Kan^R	This study
NUS5903	*rpsL1* CPS41A ΔCEP::P-*kan-cps34J*	ΔCEP::P-*kan-cps34J* x NUS0374	Kan^R	This study
NUS5904	*rpsL1* CPS47A ΔCEP::P-*kan-cps34J*	ΔCEP::P-*kan-cps34J* x NUS1080	Kan^R	This study
NUS5928	*rpsL1* CPS34 ΔCEP::P-*kan-cps34J(T333F)*	ΔCEP::P-*kan-cps34J(T333F)* x NUS0265	Kan^R	This study
**CpsJ-FLAG strains**				
NUS4830	*rpsL1* CPS14 ΔCEP::P-*kan-cps14J*-FLAG	ΔCEP::P-*kan-cps14J*-FLAG x NUS0403	Kan^R	This study
NUS4831	*rpsL1* CPS28A ΔCEP::P-*kan-cps28AJ*-FLAG	ΔCEP::P-*kan-cps28AJ*-FLAG x NUS0671	Kan^R	This study
NUS4832	*rpsL1* CPS34 ΔCEP::P-*kan-cps34J*-FLAG	ΔCEP::P-*kan-cps34J*-FLAG x NUS0265	Kan^R	This study
NUS4868	*rpsL1* CPS14 Δ*bgaA*::P_{Zn}-*cps14J cps14J<>cps14J*-FLAG	*cps14J<>cps14J*-FLAG x NUS4840	Str^R	This study
NUS4869	*rpsL1* CPS28A Δ*bgaA*::P_{Zn}-*cps28AJ cps28AJ<>cps28AJ*-FLAG	*cps28AJ<>cps28AJ*-FLAG x NUS4841	Str^R	This study
NUS4870	*rpsL1* CPS34 Δ*bgaA*::P_{Zn}-*cps34J cps34J<>cps34J*-FLAG	*cps34J<>cps34J*-FLAG x NUS4842	Str^R	This study
NUS4944	*rpsL1* CPS14 Δ*cps14E'* Δ*bgaA*::P_{Zn}-*cps14E* ΔCEP::P-*kan-cps14J*-FLAG	ΔCEP::P-*kan-cps14J*-FLAG x NUS3334	Kan^R	This study
NUS4945	*rpsL1* CPS14 Δ*cps14E'* Δ*bgaA*::P_{Zn}-*cps14E* ΔCEP::P-*kan-cps34J*-FLAG	ΔCEP::P-*kan-cps34J*-FLAG x NUS3334	Kan^R	This study
NUS4946	*rpsL1* CPS28A Δ*cps28AE*::P-*erm* Δ*bgaA*::P_{Zn}-*cps28AE* ΔCEP::P-*kan-cps28AJ*-FLAG	ΔCEP::P-*kan-cps28AJ*-FLAG x NUS4571	Kan^R, Erm^R	This study
NUS4947	*rpsL1* CPS28A Δ*cps28AE*::P-*erm* Δ*bgaA*::P_{Zn}-*cps28AE* ΔCEP::P-*kan-cps34J*-FLAG	ΔCEP::P-*kan-cps34J*-FLAG x NUS4571	Kan^R, Erm^R	This study

NUS5149	*rpsL1* Δ*bgaA*::P-*kan-cps23BJ*(P254S, G347V)-FLAG	Δ*bgaA*::P-*kan-cps23BJ*(P254S, G347V)-FLAG x IU1781	Kan^R	This study
NUS5150	*rpsL1* Δ*bgaA*::P-*kan-cps23BJ*(P254S, V249A)-FLAG	Δ*bgaA*::P-*kan-cps23BJ*(P254S, V249A)-FLAG x IU1781	Kan^R	This study
NUS5151	*rpsL1* Δ*bgaA*::P-*kan-cps23BJ*(P254S, L28F)-FLAG	Δ*bgaA*::P-*kan-cps23BJ*(P254S, L28F)-FLAG x IU1781	Kan^R	This study
**TRIO cassettes**				
NUS4770	*rpsL1* CPS12A ΔCEP::P-*kan-cps7AJ*-linker-*cps12AJ*-linker-*cps19AJ*-barcode	ΔCEP::P-*kan-cps7AJ*-linker-*cps12AJ*-linker-*cps19AJ*-barcode x NUS0669	Kan^R	This study
NUS4771	*rpsL1* CPS19A ΔCEP::P-*kan-cps7AJ*-linker-*cps12AJ*-linker-*cps19AJ*-barcode	ΔCEP::P-*kan-cps7AJ*-linker-*cps12AJ*-linker-*cps19AJ*-barcode x NUS0282	Kan^R	This study
NUS4961	*rpsL1* CPS7A ΔCEP::P-*kan-cps7AJ*-linker-*cps12AJ*-linker-*cps19AJ*-barcode	ΔCEP::P-*kan-cps7AJ*-linker-*cps12AJ*-linker-*cps19AJ*-barcode x NUS0667	Kan^R	This study
NUS4969	*rpsL1* CPS7A ΔCEP::P-*kan-cps7AJ*-barcode	ΔCEP::P-*kan-cps7AJ*-barcode x NUS0667	Kan^R	This study
NUS4805	*rpsL1* CPS19A ΔCEP::P-*kan-cps19AJ*-barcode	ΔCEP::P-*kan-cps19AJ*-barcode x NUS0282	Kan^R	This study
NUS5362	*rpsL1* CPS12A ΔCEP::P-*kan-cps12AJ*-barcode	ΔCEP::P-*kan-cps12AJ*-barcode x NUS0669	Kan^R	This study
NUS5031	*rpsL1* CPS7A ΔCEP::P-*kan-cps7AJ*-linker-*cps12AJ*-linker-*cps19AJ*-barcode Δ*cps7AJ*::P-*erm*	Δ*cps7AJ*::P-*erm* x NUS4961	Kan^R, Erm^R	This study
NUS5412	*rpsL1* CPS12A ΔCEP::P-*kan-cps7AJ*-linker-*cps12AJ*-linker-*cps19AJ*-barcode Δ*cps12AJ*::P-*erm*	Δ*cps12AJ*::P-*erm* x NUS4770	Kan^R, Erm^R	This study
NUS4823	*rpsL1* CPS19A ΔCEP::P-*kan-cps7AJ*-linker-*cps12AJ*-linker-*cps19AJ*-barcode Δ*cps19AJ*::P-*erm*	Δ*cps19AJ*::P-*erm* x NUS4771	Kan^R, Erm^R	This study
NUS5407	*rpsL1* CPS33B ΔCEP::P-*kan-cps33BJ*-barcode	ΔCEP::P-*kan-cps33BJ*-barcode x NUS0308	Kan^R	This study
NUS5579	*rpsL1* CPS33B ΔCEP::P-*kan-cps33BJ*-barcode Δ*cps33BJ*::P-*erm*	Δ*cps33BJ*::P-*erm* x NUS5407	Kan^R , Erm^R	This study
NUS5368	*rpsL1* CPS33B ΔCEP::P-*kan-cps7AJ-linker-*	ΔCEP::P-*kan-cps7AJ*-linker-*cps12AJ*-linker-	Kan^R	This study

	cps12AJ-linker-cps19AJ-barcode	*cps19AJ*-barcode x NUS0308		
NUS5561	*rpsL1* CPS33B ΔCEP::P-*kan-cps7AJ-linker-cps12AJ-linker-cps19AJ-barcode* Δ*cps33BJ*::P-*erm*	Δ*cps33BJ*::P-*erm* x NUS5368	Kan^R, Erm^R	This study
NUS5357	*rpsL1* CPS17F ΔCEP::P-*kan-cps17FJ*-barcode	ΔCEP::P-*kan-cps17FJ*-barcode x NUS0313	Kan^R	This study
NUS5562	*rpsL1* CPS17F ΔCEP::P-*kan-cps17FJ*-barcode Δ*cps17FJ*::P-*erm*	Δ*cps17FJ*::P-*erm* x NUS5357	Kan^R, Erm^R	This study
NUS5366	*rpsL1* CPS17F ΔCEP::P-*kan-cps7AJ*-linker-*cps12AJ*-linker-*cps19AJ*-barcode	ΔCEP::P-*kan-cps7AJ*-linker-*cps12AJ*-linker-*cps19AJ*-barcode x NUS0313	Kan^R	This study
NUS5563	*rpsL1* CPS17F ΔCEP::P-*kan-cps7AJ*-linker-*cps12AJ*-linker-*cps19AJ*-barcode Δ*cps17FJ*::P-*erm*	Δ*cps17FJ*::P-*erm* x NUS5366	Kan^R, Erm^R	This study
NUS4897	*rpsL1* CPS5 ΔCEP::P-*kan-cps7AJ*-linker-*cps12AJ*-linker-*cps19AJ*-barcode	ΔCEP::P-*kan-cps7AJ*-linker-*cps12AJ*-linker-*cps19AJ*-barcode x NUS0327	Kan^R	This study
NUS4961	*rpsL1* CPS7A ΔCEP::P-*kan-cps7AJ*-linker-*cps12AJ*-linker-*cps19AJ*-barcode	ΔCEP::P-*kan-cps7AJ*-linker-*cps12AJ*-linker-*cps19AJ*-barcode x NUS0667	Kan^R	This study
NUS2228	*rpsL1* CPS18B ΔCEP::P-*kan-cps7AJ*-linker-*cps12AJ*-linker-*cps19AJ*-barcode	ΔCEP::P-*kan-cps7AJ*-linker-*cps12AJ*-linker-*cps19AJ*-barcode x NUS0330	Kan^R	This study
NUS5418	*rpsL1* CPS19C ΔCEP::P-*kan-cps7AJ*-linker-*cps12AJ*-linker-*cps19AJ*-barcode	ΔCEP::P-*kan-cps7AJ*-linker-*cps12AJ*-linker-*cps19AJ*-barcode x NUS0316	Kan^R	This study
NUS2071	*rpsL1* CPS23F ΔCEP::P-*kan-cps7AJ*-linker-*cps12AJ*-linker-*cps19AJ*-barcode	ΔCEP::P-*kan-cps7AJ*-linker-*cps12AJ*-linker-*cps19AJ*-barcode x NUS0389	Kan^R	This study
NUS5419	*rpsL1* CPS27 ΔCEP::P-*kan-cps7AJ*-linker-*cps12AJ*-linker-*cps19AJ*-barcode	ΔCEP::P-*kan-cps7AJ*-linker-*cps12AJ*-linker-*cps19AJ*-barcode x NUS0463	Kan^R	This study
NUS4909	*rpsL1* CPS32F ΔCEP::P-*kan-cps7AJ*-linker-*cps12AJ*-linker-*cps19AJ*-barcode	ΔCEP::P-*kan-cps7AJ*-linker-*cps12AJ*-linker-*cps19AJ*-barcode x NUS0342	Kan^R	This study

NUS2072	*rpsL1* CPS33F ΔCEP::P-*kan-cps7AJ*-linker-*cps12AJ*-linker-*cps19AJ*-barcode	ΔCEP::P-*kan-cps7AJ*-linker-*cps12AJ*-linker-*cps19AJ*-barcode x NUS0326	Kan^{R}	This study
NUS5420	*rpsL1* CPS35C ΔCEP::P-*kan-cps7AJ*-linker-*cps12AJ*-linker-*cps19AJ*-barcode	ΔCEP::P-*kan-cps7AJ*-linker-*cps12AJ*-linker-*cps19AJ*-barcode x NUS0369	Kan^{R}	This study
NUS5421	*rpsL1* CPS41A ΔCEP::P-*kan-cps7AJ*-linker-*cps12AJ*-linker-*cps19AJ*-barcode	ΔCEP::P-*kan-cps7AJ*-linker-*cps12AJ*-linker-*cps19AJ*-barcode x NUS0374	Kan^{R}	This study
NUS5564	*rpsL1* CPS7A Δ*cps7A*(*wcwH*)::P-*erm*	Δ*cps7A*(*wcwH*)::P-*erm* x NUS0667	Erm^{R}	This study
NUS5565	*rpsL1* CPS7A Δ*cps7AE*::P-*spec-rpsL*$^{+}$ Δ*cps7A*(*wcwH*)::P-*erm*	Δ*cps7A*(*wcwH*)::P-*erm* x NUS2212	Spec^{R}, Erm^{R}	This study
NUS5566	*rpsL1* CPS7A ΔCEP::P-*kan-cps7AJ*-linker-*cps12AJ*-linker-*cps19AJ*-barcode Δ*cps7A*(*wcwH*)::P-*erm*	Δ*cps7A*(*wcwH*)::P-*erm* x NUS4961	Kan^{R}, Erm^{R}	This study
NUS2399	*rpsL1* CPS35C Δ*cps35C*(*wcrK*)::P-*erm*	Δ*cps35C*(*wcrK*)::P-*erm* x NUS0369	Erm^{R}	This study
NUS5634	*rpsL1* CPS35C Δ*cps35CE*::P-*spec-rpsL*$^{+}$ Δ*cps35C*(*wcrK*)::P-*erm*	Δ*cps35C*(*wcrK*)::P-*erm* x NUS3461	Spec^{R}, Erm^{R}	This study
NUS2190	*rpsL1* CPS35C ΔCEP::P-*kan-cps7AJ*-linker-*cps12AJ*-linker-*cps19AJ*-barcode Δ*cps35C(wcrK)*::P-*erm*	Δ*cps35C(wcrK)*::P-*erm* x NUS5420	Kan^{R}, Erm^{R}	This study

[a] Strains were constructed by transforming amplicons (left of "x") into the indicated recipient strain (right of "x") as described in *Experimental procedures*. '< >' and '::' indicate exact replacements of open reading frames and insertional mutations, respectively. Primers for constructing the strains are listed in **Table S13**.

[b] "P" refers to the constitutive synthetic promoter that drives the expression of the erythromycin, kanamycin, and spectinomycin-resistant genes, as well as the Janus cassette (59), whereas "P_{Zn}" refers to the promoter of the *czcD* operon.

[c] For Δ*cps2E* and Δ*cps2J*, 30 base pairs of the open reading frame remained intact at the 5' and 3' ends to maintain adjacent genetic signals.

[d] Selectable markers and medium supplements: Erm, erythromycin; Kan, kanamycin; Str, streptomycin; Spec, spectinomycin; Suc, sucrose. Zn^{2+}: $ZnCl_2$/$MnCl_2$ (See *Experimental procedures*).

Table S13. A list of oligonucleotides used in this study.

Primer	Sequence (5’-3’)	Template	Amplicon
For construction of Δ*CEP::P-kan-cpsJ-barcode*			
O2093	GCTGACTAGGAGGAAGGAAATG	IU5122 (56)	*CEP'-P-kan*
O1394	TAGTTCCTTATATAGTTGCATGATCCTACATTCTCCTGT GTTTTTTTATT		
O1395	GATCATGCAACTATATAAGGAACTA	CpsJ alleles (21)	*cpsI'-cpsJ-cpsK'*
O1396	TCTCCTTTCAATACTCGTACTTATT		
O1397	AATAAGTACGAGTATTGAAAGGAGAGGTGTTAACGATC GTAAACAAGGCC	Barcode (29)	*rpsL'-barcode-CEP'*
O2094	TCTTTACCTCCAATTGCCTGAA		
For construction of Δ*CEP::P-kan-rpsL*-GACCGAGGA			
O3	ATTTGCCCATCTGGCTGACTAGGAGGAA	IU5122 (56)	Δ*CEP::P-kan-rpsL-*GACCGAGGA 5’
O1917	GCCCCTTTCCTCCTCGGTCTTATGCTTTTGGACGTTTA GTACCGTATTT		
O1918	AAAAGCATAAGACCGAGGAGGAAAGGGGCCCGTCGCT TTTCATTATAGG	IU5122 (56)	Δ*CEP::P-kan-rpsL-*GACCGAGGA 3’
O4	TGGGCACGTCATTTCCAATGATTACCTG		
For construction of Δ*bgaA*::P-*kan-rpsL*$^+$			
P23	CCGTAGAACCACTATCACAAG	D39W	*bgaA* 5’
P24	TTATCCATTAAAAATCAAACGGATCCTATCCCACAGCAA ACTTACGAATGCTATAAACTC		
P1	TAGGATCCGTTTGATTTTTAATGGATAATG	HMS0001	P-*kan-rpsL*$^+$
P2	GGGCCCCTTTCCTTATGCTTTTG		
P25	AAAAGCATAAGGAAAGGGGCCCTTAGCTCTTCTAGGTT TGAGTGCAGGATTAGTAGTTAC	D39W	*bgaA* 3’
P26	GACGAAACTTTGCGGATTTG		
For construction of Δ*bgaA*::P-*sacB-kan-rpsL*$^+$			
P64	ACCAGCTACGACTCCTTCTTCT	HMS0001	*bgaA* 5'
P251	CATTATCCATTAAAAATCAAACGGATCCTATAAAATAAT AAGAAAACTCTGGATTATGG		
P249	TAGGATCCGTTTGATTTTTAATGGATAATGTTAAGGATC GATCCGTTTGATTTTTAATGG	SpnYL001	P-*sacB-kan-rpsL*$^+$
P250	GGGCCCCTTTCCTTATGCTTTTGGACGTTTAGTACCGT ATTTAGAACGG		
P252	CAAAAGCATAAGGAAAGGGGCCCGACATTGAAATTTTA TTGAAGACAGTTAAAG	HMS0001	*bgaA* 3'
P65	TCGTCCCACCACTAGATAATAGCC		
For construction of Δ*bgaA*::P-*kan-cps23BJ* variants			
P23	CCGTAGAACCACTATCACAAG	NUS1963	*bgaA*::P-*kan-cps23BJ(P254S)*
P6	TGCATGGTTACGATAGTCTTGG		
For construction of Δ*tacF*::P-*erm*			
P972	CATGCATCTGTCAAGATTTCCTTC	D39W	*tacF 5’*
P1092	CATTATCCATTAAAAATCAAACGGATCCTAAATAATATT CAAGACACGAATTCCC		
P1	TAGGATCCGTTTGATTTTTAATGGATAATG	P-*erm* cassette	P-*erm*
P2	GGGCCCCTTTCCTTATGCTTTTG		

P1093	CAAAAGCATAAGGAAAGGGGCCCATTTTCTATGAATTT TTAAACCATGTCCTAGCC	D39W	*tacF 3’*
P975	ACGCTCATAATCCTCACGATAAA		
For construction of Δ*tacF*::P-*erm*			
P1289	ATCCCAGAACCAGAGCAATC	D39W	*ytgP* 5’
P1290	CATTATCCATTAAAAATCAAACGGATCCTACGTTAGCCA AGCAGTCCCCGTAACATC		
P1	TAGGATCCGTTTGATTTTTAATGGATAATG	P-*erm* cassette	P-*erm*
P2	GGGCCCCTTTCCTTATGCTTTTG		
P1291	CAAAAGCATAAGGAAAGGGGCCCGATAAGGTAATAGG AAAAGCCCAAGCAGATC	D39W	*ytgP* 3’
P1292	TTGAGAACTGCTTCCTGAGTC		
For construction of ΔCEP::P-*kan-cps19C(wchU)*			
P3	ATTTGCCCATCTGGCTGACTAGGAGGAA	HMS0019	CEP::P-*kan*
O728	TTCTTGGAATTACAATCTTCAACTACATTCTCCTGTGTT TTTTTA		
O729	TTGAAGATTGTAATTCCAAGAATTA	PATH2463	*cps19C (wchU)*
O730	TTATAGATTGTTGTTCATATCTTGC		
O731	AAGATATGAACAACAATCTATAAGGAAAGGGGCCCGTC GCTTTTC	HMS0019	CEP′
P4	TGGGCACGTCATTTCCAATGATTACCTG		
For construction of Δ*cps19C(wchU)*::P-*erm*			
P3255	CTCGGAGAACCTATGAAACACA	PATH2463	*Cps19C (wchU) 5′*
P3256	CCATTAAAAATCAAACGGATCCTAGGACCAATCCCAGG TCAGTTG		
P1	TAGGATCCGTTTGATTTTTAATGGATAATG	P-*erm* cassette	P-*erm*
P2	GGGCCCCTTTCCTTATGCTTTTG		
P3257	CAAAAGCATAAGGAAAGGGGCCCGACACAGTTGCCAA AAGAATG	PATH2463	*Cps19C (wchU) 3′*
P3258	ACGAGGCAAATCTTGAGGAG		
For construction of Δ*cps19C(wchU)*::P-*spec-rpsL*$^+$			
P3255	CTCGGAGAACCTATGAAACACA	PATH2463	*Cps19C (wchU) 5′*
P3256	CCATTAAAAATCAAACGGATCCTAGGACCAATCCCAGG TCAGTTG		
P1	TAGGATCCGTTTGATTTTTAATGGATAATG	P-*spec-rpsL*$^+$ cassette	P-*spec-rpsL*$^+$
P2	GGGCCCCTTTCCTTATGCTTTTG		
P3257	CAAAAGCATAAGGAAAGGGGCCCGACACAGTTGCCAA AAGAATG	PATH2463	*Cps19C (wchU) 3′*
P3258	ACGAGGCAAATCTTGAGGAG		
For construction of Δ*cps28AE*::P-*erm*			
O4198	AAGGATAAACAGCCAGAGGAAG	PATH9002	Cps28AE 5’
O4121	CATTATCCATTAAAAATCAAACGGATCCTACAATGAAGA CCTTAATATTTTTCCATTC		
O1	TAGGATCCGTTTGATTTTTAATGGATAATG	P-*erm* cassette	P-*erm*
O2	GGGCCCCTTTCCTTATGCTTTTG		

O4122	GTCCAAAAGCATAAGGAAAGGGGCCCGTTGTATTGAT GAAGGATGGAGCGAAG	PATH9002	Cps28AE 3'
O4199	AGTTGATGCCAGAGCTTCTAAA		
For construction of Δ*cps33DE::P-spec-rpsL*$^+$			
O4128	TTTCTTGGAGGAGCAGTCGTAAC	NUS0672	*cps33DE 5'*
O4129	CATTATCCATTAAAAATCAAACGGATCCTATTCTCTCAC AGCACTAAGTAGATAAGTC		
O1	TAGGATCCGTTTGATTTTTAATGGATAATG	P-*spec-rpsL*$^+$ cassette	P-*spec-rpsL*$^+$
O2	GGGCCCCTTTCCTTATGCTTTTG		
O4130	CAAAAGCATAAGGAAAGGGGCCCGTTGTATTTATGAGA GATGGAGCGAAATAG	NUS0672	*cps33DE 3'*
O4131	TCGCAAGACTATACGTTCTCTG		
For construction of Δ*cps14E*::P-*sacB-kan-rpsL*$^+$			
O2312	CAGTATTACTCGTGTTTCTGATGTG	NUH0007	*cps14E 5'*
O2313	CATTATCCATTAAAAATCAAACGGATCCTACAGAAAAAT TTCCAATCCTTTTTTATCC		
O1	TAGGATCCGTTTGATTTTTAATGGATAATG	P-*sacB-kan-rpsL*$^+$ cassette	P-*sacB-kan-rpsL*$^+$
O2	GGGCCCCTTTCCTTATGCTTTTG		
O2314	CGTCCAAAAGCATAAGGAAAGGGGCCCGTTGTATTTAT GAGAAATGGAGCGAAG	NUH0007	*cps14E 3'*
O2315	CAAATTGCATCTGGTGATCATTTAC		
For construction of Δ*cps14E*'			
O2312	CAGTATTACTCGTGTTTCTGATGTG	NUH0007	*cps14E 5'*
O2669	CTTCGCTCCATTTCTCATAAATACAACCAGAAAAATTTC CAATCCTTTTTTATCC		
O2670	GGATAAAAAAGGATTGGAAATTTTTCTGGTTGTATTTAT GAGAAATGGAGCGAAG	NUH0007	*cps14E 3'*
O2315	CAAATTGCATCTGGTGATCATTTAC		
For construction of Δ*cps5E::P-spec-rpsL*$^+$			
O4112	GTTCAATCGGGAGCTGTATCAC	PATH46	*cps5E 5'*
O4113	CATTATCCATTAAAAATCAAACGGATCCTAATCTCCTAA CCGTTTTAATATACTATAC		
O1	TAGGATCCGTTTGATTTTTAATGGATAATG	P-*spec-rpsL*$^+$ cassette	P-*spec-rpsL*$^+$
O2	GGGCCCCTTTCCTTATGCTTTTG		
O4114	CAAAAGCATAAGGAAAGGGGCCCATTGTAGAGGGAAG TCAAAAAGAGAG	PATH46	*cps5E 3'*
O4115	TCTTCTCTATCAAACTGGCAGC		
For construction of Δ*cps7AE::P-spec-rpsL*$^+$			
P1583	CCCGTATTGTCTCTGTTTCAGT	PATH2477	*cps7AE 5'*
P1584	CATTATCCATTAAAAATCAAACGGATCCTATAAAATGAC AATAATACTCTGTAATAC		
O1	TAGGATCCGTTTGATTTTTAATGGATAATG	P-*spec-rpsL*$^+$ cassette	P-*spec-rpsL*$^+$
O2	GGGCCCCTTTCCTTATGCTTTTG		

P1585	CAAAAGCATAAGGAAAGGGGCCCGATATTGAAATATTG TTAAAGACCGTCAAAG	PATH2477	*cps7AE 3′*
P1586	GGATGCTAGGGCTTCCAATAG		
For construction of Δ*cps18BE::P-spec-rpsL*⁺			
O1276	AGTACCAGTTGATACCCGTATTG	PATH269	*cps18BE 5′*
O1277	CATTAAAAATCAAACGGATCCTATAAAATGACAATAATA CTCTGC		
O1	TAGGATCCGTTTGATTTTTAATGGATAATG	P-*spec-rpsL*⁺ cassette	P-*spec-rpsL*⁺
O2	GGGCCCCTTTCCTTATGCTTTTG		
O1278	CAAAAGCATAAGGAAAGGGGCCCGATATTGAAATTTTG CTTAAAAC	PATH269	*cps18BE 3′*
O1279	GCTAATGCTTCAAGTAACGAAGG		
For construction of Δ*cps19CE::P-spec-rpsL*⁺			
O4116	CAAGACTTGCAGGCAGGATC	PATH2463	*cps19CE 5′*
O4117	CATTATCCATTAAAAATCAAACGGATCCTACAGAAAAAT TTTCAATCCTTTTTCATC		
O1	TAGGATCCGTTTGATTTTTAATGGATAATG	P-*spec-rpsL*⁺ cassette	P-*spec-rpsL*⁺
O2	GGGCCCCTTTCCTTATGCTTTTG		
O4118	CAAAAGCATAAGGAAAGGGGCCCGTTGTATTTATGAGA GATGGAGCGAAGTG	PATH2463	*cps19CE 3′*
O4119	TATTTCGTGGCAAGCCCAAAC		
For construction of Δ*cps23FE::P-spec-rpsL*⁺			
P1370	CAGTCGTATCGCTAATTCTCTACGAGAAG	NUH0017	*cps23FE 5′*
P1372	CATTATCCATTAAAAATCAAACGGATCCTATAAAATAAC AAGAAAACTCTGAATTATG		
O1	TAGGATCCGTTTGATTTTTAATGGATAATG	P-*spec-rpsL*⁺ cassette	P-*spec-rpsL*⁺
O2	GGGCCCCTTTCCTTATGCTTTTG		
P1373	GTACTAAACGTCCAAAAGCATAAGGAAAGGGGCCCGA TATTGAAATTTTATTGAAG	NUH0017	*cps23FE 3′*
P1371	GTTTAGTTGATGCCAGAGCTTCTAAAAG		
For construction of Δ*cps27E::P-spec-rpsL*⁺			
N310	TCGCAATCAAGGAGACAAGG	PATH2467	*cps27E 5′*
N311	CATTATCCATTAAAAATCAAACGGATCCTATAAAATAAC AAGAAAACTCTGGATTATG		
O1	TAGGATCCGTTTGATTTTTAATGGATAATG	P-*spec-rpsL*⁺ cassette	P-*spec-rpsL*⁺
O2	GGGCCCCTTTCCTTATGCTTTTG		
N312	CAAAAGCATAAGGAAAGGGGCCCGACATTGAAATTTTA TTGAAGACAGTTAAAGTTG	PATH2467	*cps27E 3′*
N313	ACAGGAACAATGATGCTCAAATG		
For construction of Δ*cps32FE::P-spec-rpsL*⁺			
O4124	GTGTGAAACGTCCTGAAGATG	PATH2468	*cps32FE 5′*
O4125	CATTATCCATTAAAAATCAAACGGATCCTACAATGAAGA CTTTACTATTTTTCCGTTC		

O1	TAGGATCCGTTTGATTTTTAATGGATAATG	P-*spec-rpsL*$^{+}$ cassette	P-*spec-rpsL*$^{+}$
O2	GGGCCCCTTTCCTTATGCTTTTG		
O4126	CAAAAGCATAAGGAAAGGGGCCCGTTGTATTGATGAAAGATGGAGCGAAGTG	PATH2468	*cps32FE 3′*
O4127	ATCGTCCCACCACTAGATAATAGC		
For construction of Δ*cps33FE::P-spec-rpsL*$^{+}$			
O4043	TCGTCGCTGTAACGCGAGTAT	PATH101	*cps33FE 5′*
O4044	CATTATCCATTAAAAATCAAACGGATCCTACAATGAAGGCTTTACTATTTTTCCATTC		
O1	TAGGATCCGTTTGATTTTTAATGGATAATG	P-*spec-rpsL*$^{+}$ cassette	P-*spec-rpsL*$^{+}$
O2	GGGCCCCTTTCCTTATGCTTTTG		
O4045	CGTCCAAAAGCATAAGGAAAGGGGCCCGTAGTATTGATGAAGGATGGAGCGAAGTAG	PATH101	*cps33FE 3′*
O4046	CCGTTACTCTTGTAGCACGATTGC		
For construction of Δ*cps35CE::P-spec-rpsL*$^{+}$			
O4134	TAGCCAGCAAAGTTCAAGTGAC	PATH1895	*cps35CE 5′*
O4135	CATTATCCATTAAAAATCAAACGGATCCTACAATGAAGACCTTAATATTTTTCCATTC		
O1	TAGGATCCGTTTGATTTTTAATGGATAATG	P-*spec-rpsL*$^{+}$ cassette	P-*spec-rpsL*$^{+}$
O2	GGGCCCCTTTCCTTATGCTTTTG		
O4136	CAAAAGCATAAGGAAAGGGGCCCGTTGTATTGATGAAGGATGGAGCGAAGTAG	PATH1895	*cps35CE 3′*
O4137	ACTCTGGTAGGCGAAATCTC		
For construction of Δ*cps41AE::P-spec-rpsL*$^{+}$			
O4138	AAGCCAGAATATACGAGTACCACG	PATH2471	*cps41AE 5′*
O4139	CATTATCCATTAAAAATCAAACGGATCCTACAATGAAGGCTTTACTACTTTTCCATTC		
O1	TAGGATCCGTTTGATTTTTAATGGATAATG	P-*spec-rpsL*$^{+}$ cassette	P-*spec-rpsL*$^{+}$
O2	GGGCCCCTTTCCTTATGCTTTTG		
O4140	CAAAAGCATAAGGAAAGGGGCCCGTTGTATTGATGAAGGATGGAGCGAAGTAG	PATH2471	*cps41AE 3′*
O4141	TAACTCGTTGCTCCTTTGGCAATC		
For construction of Δ*bgaA*::P_{Zn}-*cps28AJ*			
O3918	ATCGCAGCCGTAGAACCACTATC	NUS0064	*bgaA*-P_{Zn} 5′
O98	ATTTCTCATTCCTTTGTTATAATAG		
O4200	CTATTATAACAAAGGAATGAGAAATTTGGAATCTGTAATGAAAAAAATATTAAATAAG	PATH9002	Cps28AJ
O187	TCACTGTTTTTTTATCATGACTATC		
O4201	GATAGTCATGATAAAAAAACAGTGATTAGCTCTTCTAGGTTTGAGTGCAGGATTAG	D39W	*bgaA 3′*
O3919	TGATCGCCCGTCTTATCACCTTAAA		
For construction of Δ*bgaA*::P_{Zn}-*cps34J*			
P86	GTTTGACTGCCGGTGTATCT	NUS0064	*bgaA*-P_{Zn} 5′

P147	ATTTCTCATTCCTTTGTTATAATAG		
P503	CTATTATAACAAAGGAATGAGAAATATGAAAGTACTAAAAAACTACGCC	CCUG2399	Cps34J
P166	TTATTTTTTAATAATATGTTTTAAT		
P504	ATTAAAACATATTATTAAAAAATAATTAGCTCTTCTAGGTTTGAGTGCAGG	D39W	*bgaA 3′*
P6	TGCATGGTTACGATAGTCTTGG		
For construction of Δ*bgaA::P$_{Zn}$-cps14J*			
O391	ATCGCAGCCGTAGAACCACTATC	NUS0064	*bgaA-P$_{Zn}$ 5′*
O4356	CTAAATTTTTACTGATTTTATTACTCATATTTCTCATTCCTTTGTTATAATAG		
O113	ATGAGTAATAAAATCAGTAAAAATT	NUH0007	*cps14J*
O114	TTATAATATATTTTCATAACCAATC		
O4357	GATTGGTTATGAAAATATATTATAATTAGCTCTTCTAGGTTTGAGTGCAGG	D39W	*bgaA 3′*
O3919	TGATCGCCCGTCTTATCACCTTAAA		
For construction of Δ*bgaA::P$_{Zn}$-cps28AE*			
O3918	ATCGCAGCCGTAGAACCACTATC	NUS0064	*bgaA-P$_{Zn}$ 5′*
O4283	GAAGACCTTAATATTTTTCCATTCATATTTCTCATTCCTTTGTTATAATAG		
O4284	ATGAATGGAAAAATATTAAGGTCTT	PATH9002	*cps28AE*
O4285	TTACTTCGCTCCATCCTTCATCAAT		
O4286	GTATTGATGAAGGATGGAGCGAAGTAATTAGCTCTTCTAGGTTTGAGTGCAGGATTAG	D39W	*bgaA 3′*
O3919	TGATCGCCCGTCTTATCACCTTAAA		
For construction of Δ*bgaA::P$_{Zn}$-cps14E*			
O95	CCGTAGAACCACTATCACAAG	NUS0064	*bgaA-P$_{Zn}$ 5′*
O98	ATTTCTCATTCCTTTGTTATAATAG		
O2671	CTATTATAACAAAGGAATGAGAAATATGGATAAAAAAGGATTGGAAATTTTTCTGGC	NUH0007	*cps14E*
O2672	TTACTTCGCTCCATTTCTCATAAAT		
O2673	GTATTTATGAGAAATGGAGCGAAGTAATTAGCTCTTCTAGGTTTGAGTGCAGGATTAG	D39W	*bgaA 3′*
O6	TGCATGGTTACGATAGTCTTGG		
For construction of Δ*cps14J::P-spec-rpsL$^+$*			
O1747	CGTGTCAACAGTATTGTAACTGGA	NUH0007	*cps14J* 5’
O1792	CATTATCCATTAAAAATCAAACGGATCCTAGGCTAAATTTTTACTGATTTTATTACTC		
O1	TAGGATCCGTTTGATTTTTAATGGATAATG	P-*spec-rpsL$^+$* cassette	P-*spec-rpsL$^+$*
O2	GGGCCCCTTTCCTTATGCTTTTG		
O1793	CCAAAAGCATAAGGAAAGGGGCCCCGAAAGATTGGTTATGAAAATATATTATAA	NUH0007	*cps14J* 3’
O1750	GGTGCTGACGAATAGTCTGAAAT		
For construction of Δ*cps28AJ::P-spec-rpsL$^+$*			
O2853	CGTGATGATGGCAGATGAGAG	PATH9002	*cps28AJ* 5’

O2854	CCATTAAAAATCAAACGGATCCTAATTTAATATTTTTTTC ATTACAGATTCC		
O1	TAGGATCCGTTTGATTTTTAATGGATAATG	P-*spec-rpsL*$^{+}$ cassette	P-*spec-rpsL*$^{+}$
O2	GGGCCCCTTTCCTTATGCTTTTG		
O2855	CAAAAGCATAAGGAAAGGGGCCCAGTTCGATAGTCAT GATAAAAAAACAGTG	PATH9002	*cps28AJ* 3’
O2856	TCCTTTACCCGACCAATTTCC		
For construction of Δ*cps34J::P-spec-rpsL*$^{+}$			
O1523	CGCGGGATTAAAGAAGCTAATG	CCUG2399	*cps34J* 5’
O2392	CCATTAAAAATCAAACGGATCCTAATTATAGGCGTAGTT TTTTAGTACTTTC		
O1	TAGGATCCGTTTGATTTTTAATGGATAATG	P-*spec-rpsL*$^{+}$ cassette	P-*spec-rpsL*$^{+}$
O2	GGGCCCCTTTCCTTATGCTTTTG		
O2393	CAAAAGCATAAGGAAAGGGGCCCAAAGAATTAAAACAT ATTATTAAAAAAATAA	CCUG2399	*cps34J* 3’
O1526	GCGAAGGCTACGGTATTCTAA		
For construction of Δ*bgaA::P-kan-cps23BJ*-FLAG			
O95	CCGTAGAACCACTATCACAAG	NUS5077-NUS5079	Cps23BJ-flag 5’
O2274	TCATTTGTCATCATCATCTTTATAATCTTTAAATCCTTTT AATAATTTTAAACATAATGG		
O2275	GATTATAAAGATGATGATGACAAATGATTAGCTCTTCTA GGTTTGAGTGCAGGATTAG	NUS5077-NUS5079	Cps23BJ-flag 3’
O6	TGCATGGTTACGATAGTCTTGG		
For construction of Δ*cps14J*<>*cps14J*-FLAG			
O1747	CGTGTCAACAGTATTGTAACTGGA	NUH0007	Cps14J-flag 5’
O4358	TTATTTGTCATCATCATCTTTATAATCTAATATATTTTCA TAACCAATCTTTCGTTT		
O4359	GATTATAAAGATGATGATGACAAATAAATTATTATTAAA GAGATTAATATTTCCCCCC	NUH0007	Cps14J-flag 3’
O1750	GGTGCTGACGAATAGTCTGAAAT		
For construction of Δ*cps28AJ*<>*cps28AJ*-FLAG			
O2853	CGTGATGATGGCAGATGAGAG	PATH9002	Cps28AJ-flag 5’
O4360	TCATTTGTCATCATCATCTTTATAATCCTGTTTTTTTATC ATGACTATCGAACTC		
O4361	GATTATAAAGATGATGATGACAAATGAGGTACACGAAT GACAGAAATAAATAAACAG	PATH9002	Cps28AJ-flag 3’
O2856	TCCTTTACCCGACCAATTTCC		
For construction of Δ*cps34J*<>*cps34J*-FLAG			
O1523	CGCGGGATTAAAGAAGCTAATG	CCUG2399	Cps34J-flag 5’
O4362	TTATTTGTCATCATCATCTTTATAATCTTTTTTAATAATAT GTTTTAATTCTTTGATATC		
O4363	GATATCAAAGAATTAAAACATATTATTAAAAAAGATTATA AAGATGATGATGACAAATAA	CCUG2399	Cps34J-flag 3’
O1526	GCGAAGGCTACGGTATTCTAA		
For construction of Δ*CEP::P-kan-cps14J-FLAG*			

O2093	GCTGACTAGGAGGAAGGAAAT	NUS2290	*CEP'-P-kan-cps14J-FLAG* 5'
O4358	TTATTTGTCATCATCATCTTTATAATCTAATATATTTTCA TAACCAATCTTTCGTTT		
O4364	GATTATAAAGATGATGATGACAAATAAGAACCAATAAGT ACGAGTATTGAAAGGAGAGG	NUS2290	*CEP'-P-kan-cps14J-FLAG* flag 3'
O2094	TCTTTACCTCCAATTGCCTGAA		
For construction of ∆*CEP::P-kan-cps28AJ-FLAG*			
O2093	GCTGACTAGGAGGAAGGAAAT	NUS2283	*CEP'-P-kan-cps28AJ-FLAG* 5'
O4360	TCATTTGTCATCATCATCTTTATAATCCTGTTTTTTTATC ATGACTATCGAACTC		
O4364	GATTATAAAGATGATGATGACAAATGAGAACCAATAAG TACGAGTATTGAAAGGAGAGG	NUS2283	*CEP'-P-kan-cps28AJ-FLAG* flag 3'
O2094	TCTTTACCTCCAATTGCCTGAA		
For construction of ∆*CEP::P-kan-cps34J-FLAG*			
O2093	GCTGACTAGGAGGAAGGAAAT	NUS2340	*CEP'-P-kan-cps34J-FLAG* 5'
O4362	TTATTTGTCATCATCATCTTTATAATCTTTTTTAATAATAT GTTTTAATTCTTTGATATC		
O4364	GATTATAAAGATGATGATGACAAATAAGAACCAATAAGT ACGAGTATTGAAAGGAGAGG	NUS2340	*CEP'-P-kan-cps34J-FLAG* flag 3'
O2094	TCTTTACCTCCAATTGCCTGAA		
For construction of ∆*CEP::P-kan-cps7AJ-linker-cps12AJ-linker-cps19AJ*			
O3	ATTTGCCCATCTGGCTGACTAGGAGGAA	NUS1051	*CEP'-P-kan-cps7AJ* 5'
O142	TCAAAAAAATCTTGTGTTTTTATTT		
O4275	CACAAGATTTTTTTGAAAACTAGACATCGAGTTCCTGCA G	PATH1706	*cps12AJ*
O178	TTAATCATGATTTTTTTCCTCCAAA		
O4276	GAAAAAAATCATGATTAAAAACTAGACATCGAGTTCCTG CAGATGAATAGCAAAATTAAA	NUS2332b	*cps19AJ-barcode-CEP' 3'*
O4	TGGGCACGTCATTTCCAATGATTACCTG		
For construction of site-directed mutant *cps34J(T333F)*			
O1523	CGCGGGATTAAAGAAGCTAATG	CCUG2399	*cps34J (T333F)* 5'
N977	GAGTTCCCATGATATTGAACCAACCGATAAAGAAC		
N978	GTTCTTTATCGGTTGGTTCAATATCATGGGAACTC	CCUG2399	*cps34J (T333F)* 3'
O1526	GCGAAGGCTACGGTATTCTAA		
**For construction of ∆*CEP::P-kan-cpsJ*-barcode**			
For construction of ∆*CEP::P-kan-cps23BJ(T38M)*-GGCGTCACC			
O3	ATTTGCCCATCTGGCTGACTAGGAGGAA	NUS1025	*CEP'-P-kan 5'*
O2163	CATTATCCATTAAAAATCAAACGGATCCTACTCGAGCTT AGCTGACTTCAACCCAC		
O1	TAGGATCCGTTTGATTTTTAATGGATAATG	NUS2863	*Cps23BJ(P254S, T38M)*
O119	TCATTTAAATCCTTTTAATAATTTT		
O2164	AAAATTATTAAAAGGATTTAAATGAGGTGTTAACGATCG TAAACAAGGCC	NUS1025	*rpsL*-GGCGTCACC-*CEP 3'*
O4	TGGGCACGTCATTTCCAATGATTACCTG		
For construction of ∆*CEP::P-kan-cps23BJ(I246T)*-TGTGCAGGT			
O3	ATTTGCCCATCTGGCTGACTAGGAGGAA	NUS1005	*CEP'-P-kan 5'*
O2163	CATTATCCATTAAAAATCAAACGGATCCTACTCGAGCTT AGCTGACTTCAACCCAC		

O1	TAGGATCCGTTTGATTTTTAATGGATAATG	NUS2864	*Cps23BJ(P254S, I246T)*
O119	TCATTTAAATCCTTTTAATAATTTT		
O2164	AAAATTATTAAAAGGATTTAAATGAGGTGTTAACGATCGTAAACAAGGCC	NUS1005	*rpsL*-TGTGCAGGT-*CEP 3'*
O4	TGGGCACGTCATTTCCAATGATTACCTG		
For construction of Δ*CEP::P-kan-cps23BJ(Q276L)*-GGGCCGACG			
O3	ATTTGCCCATCTGGCTGACTAGGAGGAA	NUS0995	*CEP'-P-kan 5'*
O2163	CATTATCCATTAAAAATCAAACGGATCCTACTCGAGCTTAGCTGACTTCAACCCAC		
O1	TAGGATCCGTTTGATTTTTAATGGATAATG	NUS2865	*Cps23BJ(P254S, Q276L)*
O119	TCATTTAAATCCTTTTAATAATTTT		
O2164	AAAATTATTAAAAGGATTTAAATGAGGTGTTAACGATCGTAAACAAGGCC	NUS0995	*rpsL*-GGGCCGACG-*CEP 3'*
O4	TGGGCACGTCATTTCCAATGATTACCTG		
For construction of Δ*CEP::P-kan-cps23BJ(L414P)*-CCCTGATTT			
O3	ATTTGCCCATCTGGCTGACTAGGAGGAA	NUS0598	*CEP'-P-kan 5'*
O2163	CATTATCCATTAAAAATCAAACGGATCCTACTCGAGCTTAGCTGACTTCAACCCAC		
O1	TAGGATCCGTTTGATTTTTAATGGATAATG	NUS2866	*Cps23BJ(P254S, L414P)*
O119	TCATTTAAATCCTTTTAATAATTTT		
O2164	AAAATTATTAAAAGGATTTAAATGAGGTGTTAACGATCGTAAACAAGGCC	NUS0598	*rpsL*-CCCTGATTT-*CEP 3'*
O4	TGGGCACGTCATTTCCAATGATTACCTG		
**For construction of Δ*cpsJ::*P-*erm***			
For construction of Δ*cps1J::*P-*erm*			
O3066	CTTGTAATGTTAGTTCCGCTTGC	PATH106	*cps1J* 5'
O2746	CCATTAAAAATCAAACGGATCCTATAAAATATAAAAAATTTCTACTAAG		
O1	TAGGATCCGTTTGATTTTTAATGGATAATG	P-*erm* cassette	P-*erm*
O2	GGGCCCCTTTCCTTATGCTTTTG		
O2747	CCAAAAGCATAAGGAAAGGGGCCCTTTTTTATTATAGATTATCTTAGAAATTG	PATH106	*cps1J* 3'
O3067	CTGCCTTGATTAGCTCCTGTT		
For construction of Δ*cps2J::*P-*erm*			
O93	GAACATGGAAATGTGGAAGATGAG	D39W	*cps2J* 5'
O2064	CCATTAAAAATCAAACGGATCCTATTTTAGTAAGTAATTATATCTTCTAC		
O1	TAGGATCCGTTTGATTTTTAATGGATAATG	P-*erm* cassette	P-*erm*
O2	GGGCCCCTTTCCTTATGCTTTTG		
O2065	CAAAAGCATAAGGAAAGGGGCCCATTGGTGAATTAAAAAAGTTTCTAAC	D39W	*cps2J* 3'
O94	CATAGCCGAAGGAAGGATTGT		
For construction of Δ*cps4J::*P-*erm*			
O2301	AGGGCATAATGCACGTTT	CCUG37285	*cps4J* 5'
O2302	CCATTAAAAATCAAACGGATCCTATTTTATAAATGAAATTCTATCAACTTTC		
O1	TAGGATCCGTTTGATTTTTAATGGATAATG	P-*erm* cassette	P-*erm*
O2	GGGCCCCTTTCCTTATGCTTTTG		
O2303	CAAAAGCATAAGGAAAGGGGCCCAACCGTTCATTATTTGTTAGGAGTAAG	CCUG37285	*cps4J* 3'
O2304	CTTCCTGAATACCACCACTATC		
For construction of Δ*cps5J::*P-*erm*			
O2305	GGGATACCTACACAACTTCTGATT	PATH46	*cps5J* 5'

O2306	CCATTAAAAATCAAACGGATCCTATAGACTAACTTGTTTCAGAATGTTCACC		
O1	TAGGATCCGTTTGATTTTTAATGGATAATG	P-*erm* cassette	P-*erm*
O2	GGGCCCCTTTCCTTATGCTTTTG		
O2307	CAAAAGCATAAGGAAAGGGGCCCAAATATATAGAAAGTAGGAAAAACGTATG	PATH46	*cps5J* 5’
O2308	TCTCTTCCTTCTTTCTAATGTCCTAAG		
For construction of Δ*cps6AJ*::P-*erm*			
O3085	GGGAGTCCTAAATCTCAACTTACC	NUH0002	*cps6AJ* 5’
O2952	CCATTAAAAATCAAACGGATCCTACAAATTTGTTATAAGAAACTTC		
O1	TAGGATCCGTTTGATTTTTAATGGATAATG	P-*erm* cassette	P-*erm*
O2	GGGCCCCTTTCCTTATGCTTTTG		
O2950	CCAAAAGCATAAGGAAAGGGGCCCGAATATTTTAGAAAGAAAATATTTG	NUH0002	*cps6AJ* 3’
O2953	CGTCACCGATAAACTCTTCCC		
For construction of Δ*cps6BJ*::P-*erm*			
O2948	ATGCAATCATTAGTGGTGGTAGA	NUS0003	*cps6BJ* 5’
O2952	CCATTAAAAATCAAACGGATCCTACAAATTTGTTATAAGAAACTTC		
O1	TAGGATCCGTTTGATTTTTAATGGATAATG	P-*erm* cassette	P-*erm*
O2	GGGCCCCTTTCCTTATGCTTTTG		
O2950	CCAAAAGCATAAGGAAAGGGGCCCGAATATTTTAGAAAGAAAATATTTG	NUS0003	*cps6BJ* 3’
O2951	CCAGGGCCGTGATAGATATTG		
For construction of Δ*cps6CJ*::P-*erm*			
O2948	ATGCAATCATTAGTGGTGGTAGA	NUH0004	*cps6CJ* 5’
O2949	CCATTAAAAATCAAACGGATCCTATAAATTTGTTATAAGAAACTTC		
O1	TAGGATCCGTTTGATTTTTAATGGATAATG	P-*erm* cassette	P-*erm*
O2	GGGCCCCTTTCCTTATGCTTTTG		
O2950	CCAAAAGCATAAGGAAAGGGGCCCGAATATTTTAGAAAGAAAATATTTG	NUH0004	*cps6CJ* 3’
O2951	CCAGGGCCGTGATAGATATTG		
For construction of Δ*cps6DJ*::P-*erm*			
O3298	ATTCCAGCGACTACACTTATTTCT	PATH3390	*cps6DJ* 5’
O2952	CCATTAAAAATCAAACGGATCCTACAAATTTGTTATAAGAAACTTC		
O1	TAGGATCCGTTTGATTTTTAATGGATAATG	P-*erm* cassette	P-*erm*
O2	GGGCCCCTTTCCTTATGCTTTTG		
O2950	CCAAAAGCATAAGGAAAGGGGCCCGAATATTTTAGAAAGAAAATATTTG	PATH3390	*cps6DJ* 3’
O3299	GAACGAGGGTATTCTGGCTTT		
For construction of Δ*cps7AJ*::P-*erm*			
O2286	CCAAACTATTACAGTGGGAATTACG	PATH2477	*cps7AJ* 5’
O2287	CCATTAAAAATCAAACGGATCCTATTTATAAGCATAATTTTTTATTGTTTTC		
O1	TAGGATCCGTTTGATTTTTAATGGATAATG	P-*erm* cassette	P-*erm*
O2	GGGCCCCTTTCCTTATGCTTTTG		
O2288	CAAAAGCATAAGGAAAGGGGCCCTTATTAAATAAAAACACAAGATTTTTTTG	PATH2477	*cps7AJ* 3’
O2289	GGTTGGGCTAAGGTCAATACA		
For construction of Δ*cps7BJ*::P-*erm*			
O2290	GTGACTGTGAGTCCTAACGATATT	PATH1803	*cps7BJ* 5’

O2291	CCATTAAAAATCAAACGGATCCTAAGCATTTTTATTTATTGATTTTTCTTTC		
O1	TAGGATCCGTTTGATTTTTAATGGATAATG	P-*erm* cassette	P-*erm*
O2	GGGCCCCTTTCCTTATGCTTTTG		
O2292	CAAAAGCATAAGGAAAGGGGCCCAATCAAATTTTTTATAAGGATATTTC	PATH1803	*cps7BJ* 3’
O2293	GAACTTTCCCTCCATCTGCTAC		
For construction of Δ*cps7CJ::*P-*erm*			
O2290	GTGACTGTGAGTCCTAACGATATT	PATH203	*cps7CJ* 5’
O2294	CCATTAAAAATCAAACGGATCCTATTTATTTATTGATTTTTCTTTCATGCTC		
O1	TAGGATCCGTTTGATTTTTAATGGATAATG	P-*erm* cassette	P-*erm*
O2	GGGCCCCTTTCCTTATGCTTTTG		
O2292	CAAAAGCATAAGGAAAGGGGCCCAATCAAATTTTTTATAAGGATATTTC	PATH203	*cps7CJ* 3’
O2293	GAACTTTCCCTCCATCTGCTAC		
For construction of Δ*cps7FJ::*P-*erm*			
O2286	CCAAACTATTACAGTGGGAATTACG	NUH0005	*cps7FJ* 5’
O2295	CCATTAAAAATCAAACGGATCCTATATTGATTGCAACTCCCCATGAAAG		
O1	TAGGATCCGTTTGATTTTTAATGGATAATG	P-*erm* cassette	P-*erm*
O2	GGGCCCCTTTCCTTATGCTTTTG		
O2288	CAAAAGCATAAGGAAAGGGGCCCTTATTAAATAAAAACACAAGATTTTTTTG	NUH0005	*cps7FJ* 3’
O2289	GGTTGGGCTAAGGTCAATACA		
For construction of Δ*cps8J::*P-*erm*			
O2309	CAAGAGGGAGTTGTTGGAGAAA	NUH0006	*cps8J* 5’
O2259	CATTATCCATTAAAAATCAAACGGATCCTAAAAATTTCTAGTAATAGATTTTTTAG		
O1	TAGGATCCGTTTGATTTTTAATGGATAATG	P-*erm* cassette	P-*erm*
O2	GGGCCCCTTTCCTTATGCTTTTG		
O2260	CGTCCAAAAGCATAAGGAAAGGGGCCCCTAAATCGGTTTAATATTATTAAAGAATG	NUH0006	*cps8J* 3’
O2310	ACTGGAATCGAAGAAACTAACATCT		
For construction of Δ*cps9AJ::*P-*erm*			
O2888	CGCTACTGACAATGTTGATTTCAT	PATH4969	*cps9AJ* 5’
O2889	CCATTAAAAATCAAACGGATCCTAACCTACCTTAAACTTATTACTTATATCC		
O1	TAGGATCCGTTTGATTTTTAATGGATAATG	P-erm cassette	P-*erm*
O2	GGGCCCCTTTCCTTATGCTTTTG		
O2890	CAAAAGCATAAGGAAAGGGGCCCAAGCAGTTAATAATGATGTTTAGTAGGTG	PATH4969	*cps9AJ* 3’
O2891	CTATCTGAAGTGATGACCGGAAG		
For construction of Δ*cps9VJ::*P-*erm*			
O2888	CGCTACTGACAATGTTGATTTCAT	PATH30	*cps9VJ* 5’
O2889	CCATTAAAAATCAAACGGATCCTAACCTACCTTAAACTTATTACTTATATCC		
O1	TAGGATCCGTTTGATTTTTAATGGATAATG	P-*erm* cassette	P-*erm*
O2	GGGCCCCTTTCCTTATGCTTTTG		
O2890	CAAAAGCATAAGGAAAGGGGCCCAAGCAGTTAATAATGATGTTTAGTAGG	PATH30	*cps9VJ* 3’
O2891	CTATCTGAAGTGATGACCGGAAG		
For construction of Δ*cps9LJ::*P-*erm*			
O2221	AAGTTGCATGAGCAAGTTAC	PATH2478	*cps9LJ* 5’

O2222	CCATTAAAAATCAAACGGATCCTATCCACGTTTCAAATT TTTTTCAAC		
O1	TAGGATCCGTTTGATTTTTAATGGATAATG	P-*erm* cassette	P-*erm*
O2	GGGCCCCTTTCCTTATGCTTTTG		
O2223	CCAAAAGCATAAGGAAAGGGGCCCCGACGAGTCTTCG CGGTTATAAG	PATH2478	*cps9LJ* 3’
O2224	GTATTCTAGGATCACTCTCAATTTC		
For construction of Δ*cps9NJ::*P-*erm*			
O2221	AAGTTGCATGAGCAAGTTAC	PATH98	*cps9NJ* 5’
O2222	CCATTAAAAATCAAACGGATCCTATCCACGTTTCAAATT TTTTTCAAC		
O1	TAGGATCCGTTTGATTTTTAATGGATAATG	P-erm cassette	P-*erm*
O2	GGGCCCCTTTCCTTATGCTTTTG		
O2223	CCAAAAGCATAAGGAAAGGGGCCCCGACGAGTCTTCG CGGTTATAAG	PATH98	*cps9NJ* 3’
O2224	GTATTCTAGGATCACTCTCAATTTC		
For construction of Δ*cps10AJ::*P-*erm*			
O633	CGAGGAAATATGAAACAGGCTTAT	PATH691	*cps10AJ* 5’
O2916	CCATTAAAAATCAAACGGATCCTAGTTATAGGCGTAATT TTTTAGTACTTTC		
O1	TAGGATCCGTTTGATTTTTAATGGATAATG	P-*erm* cassette	P-*erm*
O2	GGGCCCCTTTCCTTATGCTTTTG		
O2917	CAAAAGCATAAGGAAAGGGGCCCAAAGAATTAAAACAA ATCATAAAAAAATAATG	PATH691	*cps10AJ* 3’
O636	GCTGCTTCCTCTTGGTACTT		
For construction of Δ*cps10CJ::*P-*erm*			
O3453	CAGGAAACACATCAAGCAGAC	PATH2460	*cps10CJ* 5’
O3675	CATTATCCATTAAAAATCAAACGGATCCTAATTGTAGGC GTAGTTTTTTAATACTTTCATTTC		
O1	TAGGATCCGTTTGATTTTTAATGGATAATG	P-*erm* cassette	P-*erm*
O2	GGGCCCCTTTCCTTATGCTTTTG		
O3676	CTAAACGTCCAAAAGCATAAGGAAAGGGGCCCGAATTA AAACAAATTATTAGGAAAAAATAAAATG	PATH2460	*cps10CJ* 3’
O3454	CTCCCATTGTTTGATTAGAAAGGA		
For construction of Δ*cps10FJ::*P-*erm*			
O2652	GAGGTAATCCTTGTAAATGATGGTTC	PATH1539	*cps10FJ* 5’
O2653	CCATTAAAAATCAAACGGATCCTAATTGTAGGCGTAGT TTTTTAATACTTTC		
O1	TAGGATCCGTTTGATTTTTAATGGATAATG	P-erm cassette	P-*erm*
O2	GGGCCCCTTTCCTTATGCTTTTG		
O2656	CAAAAGCATAAGGAAAGGGGCCCGAATTAAAACAAATT ATTAGGAAAAAATAG	PATH1539	*cps10FJ* 3’
O2655	CCAGCACCGACGATAAGATAAT		
For construction of Δ*cps12AJ::*P-*erm*			
O2297	ACAAGTATGCCCAATTTGTCT	PATH1706	*cps12AJ* 5’
O2298	CCATTAAAAATCAAACGGATCCTAATTAAAAAAACTATT TTTTACACGACTC		
O1	TAGGATCCGTTTGATTTTTAATGGATAATG	P-*erm* cassette	P-*erm*
O2	GGGCCCCTTTCCTTATGCTTTTG		
O2299	CAAAAGCATAAGGAAAGGGGCCCAAAAATTTGGAGGA AAAAAATCATGATTAATG	PATH1706	*cps12AJ* 3’
O2300	TAGCAGGTGTTGCCAGAT		
For construction of Δ*cps12BJ::*P-*erm*			
O2297	ACAAGTATGCCCAATTTGTCT	PATH2479	*cps12BJ* 5’

O2298	CCATTAAAAATCAAACGGATCCTAATTAAAAAAACTATTTTTTACACGACTC		
O1	TAGGATCCGTTTGATTTTTAATGGATAATG	P-*erm* cassette	P-*erm*
O2	GGGCCCCTTTCCTTATGCTTTTG		
O2299	CAAAAGCATAAGGAAAGGGGCCCAAAAATTTGGAGGAAAAAAATCATGATTAATG	PATH2479	*cps12BJ* 3’
O2300	TAGCAGGTGTTGCCAGAT		
For construction of Δ*cps12FJ::*P-*erm*			
O2297	ACAAGTATGCCCAATTTGTCT	PATH122	*cps12FJ* 5’
O2298	CCATTAAAAATCAAACGGATCCTAATTAAAAAAACTATTTTTTACACGACTC		
O1	TAGGATCCGTTTGATTTTTAATGGATAATG	P-*erm* cassette	P-*erm*
O2	GGGCCCCTTTCCTTATGCTTTTG		
O2299	CAAAAGCATAAGGAAAGGGGCCCAAAAATTTGGAGGAAAAAAATCATGATTAATG	PATH122	*cps12FJ* 3’
O2300	TAGCAGGTGTTGCCAGAT		
For construction of Δ*cps13J::*P-*erm*			
O556	AGGGCAGTATTCTACTACTCTACA	PATH1886	*cps13J* 5’
O2892	CCATTAAAAATCAAACGGATCCTAATTGTAGGCGTAGTTTTTTAAGATTTTC		
O1	TAGGATCCGTTTGATTTTTAATGGATAATG	P-erm cassette	P-*erm*
O2	GGGCCCCTTTCCTTATGCTTTTG		
O2893	CAAAAGCATAAGGAAAGGGGCCCGAATTAAAACAAATTATTAGGAAAAATTAG	PATH1886	*cps13J* 3’
O559	TCATTCAAAGTAAGCGTGAGGA		
For construction of Δ*cps14J::*P-*erm*			
O1747	CGTGTCAACAGTATTGTAACTGGA	NUH0007	*cps14J* 5’
O1792	CATTATCCATTAAAAATCAAACGGATCCTAGGCTAAATTTTTACTGATTTTATTACTC		
O1	TAGGATCCGTTTGATTTTTAATGGATAATG	P-*erm* cassette	P-*erm*
O2	GGGCCCCTTTCCTTATGCTTTTG		
O1793	CCAAAAGCATAAGGAAAGGGGCCCCGAAAGATTGGTTATGAAAATATATTATAA	NUH0007	*cps14J* 3’
O1750	GGTGCTGACGAATAGTCTGAAAT		
For construction of Δ*cps15AJ::*P-*erm*			
O2495	ACGACAGTTTAGAAAGTTCGCTA	CCUG69630	*cps15AJ* 5’
O2496	CCATTAAAAATCAAACGGATCCTAGGCTAAATTTTTACTAATTTTATTACTC		
O1	TAGGATCCGTTTGATTTTTAATGGATAATG	P-*erm* cassette	P-*erm*
O2	GGGCCCCTTTCCTTATGCTTTTG		
O2497	CAAAAGCATAAGGAAAGGGGCCCCGAAAGGTTGTACATAAAAATACATTATAA	CCUG69630	*cps15AJ* 3’
O2498	AAGTCGCATATTTATCTCCAATTCC		
For construction of Δ*cps15BJ::*P-*erm*			
O2495	ACGACAGTTTAGAAAGTTCGCTA	NUH0009	*cps15BJ* 5’
O2496	CCATTAAAAATCAAACGGATCCTAGGCTAAATTTTTACTAATTTTATTACTC		
O1	TAGGATCCGTTTGATTTTTAATGGATAATG	P-*erm* cassette	P-*erm*
O2	GGGCCCCTTTCCTTATGCTTTTG		
O2497	CAAAAGCATAAGGAAAGGGGCCCCGAAAGGTTGTACATAAAAATACATTATAA	NUH0009	*cps15BJ* 3’
O2499	AATTGAATCGCTAATTGTAGCTTCC		
For construction of Δ*cps15CJ::*P-*erm*			
O2495	ACGACAGTTTAGAAAGTTCGCTA	NUH0010	*cps15CJ* 5’

O2496	CCATTAAAAATCAAACGGATCCTAGGCTAAATTTTTACT AATTTTATTACTC		
O1	TAGGATCCGTTTGATTTTTAATGGATAATG	P-*erm* cassette	P-*erm*
O2	GGGCCCCTTTCCTTATGCTTTTG		
O2497	CAAAAGCATAAGGAAAGGGGCCCCGAAAGGTTGTACA TAAAAATACATTATAA	NUH0010	*cps15CJ* 3’
O2499	AATTGAATCGCTAATTGTAGCTTCC		
For construction of Δ*cps15FJ::*P-*erm*			
O2495	ACGACAGTTTAGAAAGTTCGCTA	NUH0011	*cps15FJ* 5’
O2496	CCATTAAAAATCAAACGGATCCTAGGCTAAATTTTTACT AATTTTATTACTC		
O1	TAGGATCCGTTTGATTTTTAATGGATAATG	P-*erm* cassette	P-*erm*
O2	GGGCCCCTTTCCTTATGCTTTTG		
O2497	CAAAAGCATAAGGAAAGGGGCCCCGAAAGGTTGTACA TAAAAATACATTATAA	NUH0011	*cps15FJ* 3’
O2499	AATTGAATCGCTAATTGTAGCTTCC		
For construction of Δ*cps16FJ::*P-*erm*			
O2812	GGCACCTGAAACTCGGAAA	PATH1702	*cps16FJ* 5’
O2813	CCATTAAAAATCAAACGGATCCTAATTTAATATTTTTTTC ATGACAGATTCC		
O1	TAGGATCCGTTTGATTTTTAATGGATAATG	P-*erm* cassette	P-*erm*
O2	GGGCCCCTTTCCTTATGCTTTTG		
O2814	CAAAAGCATAAGGAAAGGGGCCCAGTTCGATAGCTAT CATTAAACAACAGTG	PATH1702	*cps16FJ* 3’
O2815	TACTCTTTGACATCACTGCGTT		
For construction of Δ*cps17AJ::*P-*erm*			
O2931	TTCTAGATTCACTTCTGCTTATGGT	PATH2480	*cps17AJ* 5’
O2932	CCATTAAAAATCAAACGGATCCTACTTGGAGCTTAAACT TTTTTCG		
O1	TAGGATCCGTTTGATTTTTAATGGATAATG	P-*erm* cassette	P-*erm*
O2	GGGCCCCTTTCCTTATGCTTTTG		
O2933	CCAAAAGCATAAGGAAAGGGGCCCTTGAAAAATAGATT TTTGAGCACTG	PATH2480	*cps17AJ* 3’
O2934	AGAGTTTAACTGCTTCAGCTTCT		
For construction of Δ*cps17FJ::*P-*erm*			
O2935	AAAGTTCCAACAACAACGGAAA	PATH680	*cps17FJ* 5’
O2936	CCATTAAAAATCAAACGGATCCTATCTTCTAGTATTAAC AAGTCTATTAC		
O1	TAGGATCCGTTTGATTTTTAATGGATAATG	P-erm cassette	P-*erm*
O2	GGGCCCCTTTCCTTATGCTTTTG		
O2937	CTAAACGTCCAAAAGCATAAGGAAAGGGGCCCAAAATA ATCAAGTCG	PATH680	*cps17FJ* 3’
O2938	CCATGCGATAGGCAATTTCTTC		
For construction of Δ*cps18AJ::*P-*erm*			
O2939	CTTGTGGTGGATTCGGAGTAAG	PATH4560	*cps18AJ* 5’
O2940	CCATTAAAAATCAAACGGATCCTAGTAAACAATATTTTT CTTTAAAC		
O1	TAGGATCCGTTTGATTTTTAATGGATAATG	P-*erm* cassette	P-*erm*
O2	GGGCCCCTTTCCTTATGCTTTTG		
O2941	CCAAAAGCATAAGGAAAGGGGCCCGAACGTACTCATTT ATTTAAAAGG	PATH4560	*cps18AJ* 3’
O2942	GGTTGATTCAATTCCCTTCCATAAA		
For construction of Δ*cps18BJ::*P-*erm*			
O2943	CCTGTTGTTATTCACGCCTTAC	PATH269	*cps18BJ* 5’

O2944	CCATTAAAAATCAAACGGATCCTAGTAAACAATGTTTTTCTTTAAATTAG		
O1	TAGGATCCGTTTGATTTTTAATGGATAATG	P-*erm* cassette	P-*erm*
O2	GGGCCCCTTTCCTTATGCTTTTG		
O2945	CCAAAAGCATAAGGAAAGGGGCCCGAACATACTCATTTATTTAAAAGG	PATH269	*cps18BJ* 3'
O2946	CAACTCCCTTCCATAAATCTGTTC		
For construction of Δ*cps18CJ::*P-*erm*			
O2943	CCTGTTGTTATTCACGCCTTAC	NUH0012	*cps18CJ* 5'
O2944	CCATTAAAAATCAAACGGATCCTAGTAAACAATGTTTTTCTTTAAATTAG		
O1	TAGGATCCGTTTGATTTTTAATGGATAATG	P-*erm* cassette	P-*erm*
O2	GGGCCCCTTTCCTTATGCTTTTG		
O2945	CCAAAAGCATAAGGAAAGGGGCCCGAACATACTCATTTATTTAAAAGG	NUH0012	*cps18CJ* 3'
O2946	CAACTCCCTTCCATAAATCTGTTC		
For construction of Δ*cps18FJ::*P-*erm*			
O2947	CTTACGAAATCGTTGGCCTTTATG	PATH4599	*cps18FJ* 5'
O2944	CCATTAAAAATCAAACGGATCCTAGTAAACAATGTTTTTCTTTAAATTAG		
O1	TAGGATCCGTTTGATTTTTAATGGATAATG	P-*erm* cassette	P-*erm*
O2	GGGCCCCTTTCCTTATGCTTTTG		
O2945	CCAAAAGCATAAGGAAAGGGGCCCGAACATACTCATTTATTTAAAAGG	PATH4599	*cps18FJ* 3'
O2946	CAACTCCCTTCCATAAATCTGTTC		
For construction of Δ*cps19AJ::*P-*erm*			
O2918	CAGTGGTTCTTTAGAAATGCTACC	NUH0013	*cps19AJ* 5'
O2919	CCATTAAAAATCAAACGGATCCTAAGTTAGTATATTTTTAATTTTGCTATTC		
O1	TAGGATCCGTTTGATTTTTAATGGATAATG	P-erm cassette	P-*erm*
O2	GGGCCCCTTTCCTTATGCTTTTG		
O2920	CAAAAGCATAAGGAAAGGGGCCCTTTAAAGCTAGTAAAAAAACATCAAATTAA	NUH0013	*cps19AJ* 3'
O2921	TCTGTTGACTAGCATCACCATTA		
For construction of Δ*cps19BJ::*P-*erm*			
O2229	TGCTCAAGAGAAGGACAGTTATT	PATH2606	*cps19BJ* 5'
O2230	CCATTAAAAATCAAACGGATCCTATGCATTCAACTTTATGGATTTATTCCCC		
O1	TAGGATCCGTTTGATTTTTAATGGATAATG	P-*erm* cassette	P-*erm*
O2	GGGCCCCTTTCCTTATGCTTTTG		
O2231	CAAAAGCATAAGGAAAGGGGCCCTTATTTTCTCTTCTTGCAAAGAAGAAG	PATH2606	*cps19BJ* 3'
O2232	AGTCTCCTCATCAGTCCCAA		
For construction of Δ*cps19CJ::*P-*erm*			
O2229	TGCTCAAGAGAAGGACAGTTATT	PATH2463	*cps19CJ* 5'
O2230	CCATTAAAAATCAAACGGATCCTATGCATTCAACTTTATGGATTTATTCCCC		
O1	TAGGATCCGTTTGATTTTTAATGGATAATG	P-*erm* cassette	P-*erm*
O2	GGGCCCCTTTCCTTATGCTTTTG		
O2231	CAAAAGCATAAGGAAAGGGGCCCTTATTTTCTCTTCTTGCAAAGAAGAAG	PATH2463	*cps19CJ* 3'
O2394	GTCGTATCTCGCATGACCAA		
For construction of Δ*cps19FJ::*P-*erm*			
O2922	CAACCTTGTTCTTAGTAATGGATATACG	NUH0014	*cps19FJ* 5'

O2923	CCATTAAAAATCAAACGGATCCTAAGTTATTATATTTTTA ATTTTAGTATTC		
O1	TAGGATCCGTTTGATTTTTAATGGATAATG	P-*erm* cassette	P-*erm*
O2	GGGCCCCTTTCCTTATGCTTTTG		
O2924	CAAAAGCATAAGGAAAGGGGCCCTACTTTAAAAATCAT AAAAAAATATCATAA	NUH0014	*cps19FJ* 3’
O2232	AGTCTCCTCATCAGTCCCAA		
For construction of Δ*cps20J::*P-*erm*			
O2383	GATTCAACAATGTAGTGGCGATAA	PATH682	*cps20J* 5’
O2384	CCATTAAAAATCAAACGGATCCTAATTGTAAGCATAGTT TTTTATTACTTTC		
O1	TAGGATCCGTTTGATTTTTAATGGATAATG	P-*erm* cassette	P-*erm*
O2	GGGCCCCTTTCCTTATGCTTTTG		
O2385	CAAAAGCATAAGGAAAGGGGCCCGAATTAAAACAAATT ATTAGGAAAAACTAG	PATH682	*cps20J* 3’
O2386	CAAACCAGCACCGACAATAAG		
For construction of Δ*cps21J::*P-*erm*			
O2894	AGACAAGGACTACCTGTTAATGG	PATH57	*cps21J* 5’
O2895	CCATTAAAAATCAAACGGATCCTAATTATAGGCGTAATT TTTAAGAACTTTC		
O1	TAGGATCCGTTTGATTTTTAATGGATAATG	P-*erm* cassette	P-*erm*
O2	GGGCCCCTTTCCTTATGCTTTTG		
O2896	CAAAAGCATAAGGAAAGGGGCCCCAGGAACTATTAAAA AATAGGGAGATTTAA	PATH57	*cps21J* 3’
O2897	GCTAACCGTCCACAGAAGATAA		
For construction of Δ*cps22AJ::*P-*erm*			
O2225	TGAGGTGAATCCTGCGTTTAG	NUH0015	*cps22AJ* 5’
O2226	CATTAAAAATCAAACGGATCCTAATTTTTAATAAGTGAA TTTCTGGTTG		
O1	TAGGATCCGTTTGATTTTTAATGGATAATG	P-*erm* cassette	P-*erm*
O2	GGGCCCCTTTCCTTATGCTTTTG		
O2227	CCAAAAGCATAAGGAAAGGGGCCCTTTACAGGTATTAT AAAGAG	NUH0015	*cps22AJ* 3’
O2228	GTCTTTGGCTGCATTCCATATTC		
For construction of Δ*cps22FJ::*P-*erm*			
O2225	TGAGGTGAATCCTGCGTTTAG	PATH115	*cps22FJ* 5’
O2226	CATTAAAAATCAAACGGATCCTAATTTTTAATAAGTGAA TTTCTGGTTG		
O1	TAGGATCCGTTTGATTTTTAATGGATAATG	P-*erm* cassette	P-*erm*
O2	GGGCCCCTTTCCTTATGCTTTTG		
O2227	CCAAAAGCATAAGGAAAGGGGCCCTTTACAGGTATTAT AAAGAG	PATH115	*cps22FJ* 3’
O2228	GTCTTTGGCTGCATTCCATATTC		
For construction of Δ*cps23AJ::*P-*erm*			
O2816	AAAGCAGTCGGGATGAGA	NUH0016	*cps23AJ* 5’
O2192	CCATTAAAAATCAAACGGATCCTAATTTTTTGCTAATTC CTTATATTTACTC		
O1	TAGGATCCGTTTGATTTTTAATGGATAATG	P-*erm* cassette	P-*erm*
O2	GGGCCCCTTTCCTTATGCTTTTG		
O2817	CAAAAGCATAAGGAAAGGGGCCCTTATATTTAAAAATTT TCAAAAGAAATTAG	NUH0016	*cps23AJ* 3’
O2818	AGGCAGTAAGTCATACAATTCTTC		
For construction of Δ*cps23BJ::*P-*erm*			
O646	TGAGAAATCGAGTCTATGGATCTTAC	PATH212	*cps23BJ* 5’

O2192	CCATTAAAAATCAAACGGATCCTAATTTTTTGCTAATTC CTTATATTTACTC		
O1	TAGGATCCGTTTGATTTTTAATGGATAATG	P-*erm* cassette	P-*erm*
O2	GGGCCCCTTTCCTTATGCTTTTG		
O2193	CAAAAGCATAAGGAAAGGGGCCCTGTTTAAAATTATTA AAAGGATTTAAATG	PATH212	*cps23BJ* 3’
O649	CATTCACATATCTGACATAAGGCTTG		
For construction of Δ*cps23FJ*::P-*erm*			
O2816	AAAGCAGTCGGGATGAGA	NUH0017	*cps23FJ* 5’
O2192	CCATTAAAAATCAAACGGATCCTAATTTTTTGCTAATTC CTTATATTTACTC		
O1	TAGGATCCGTTTGATTTTTAATGGATAATG	P-*erm* cassette	P-*erm*
O2	GGGCCCCTTTCCTTATGCTTTTG		
O2817	CAAAAGCATAAGGAAAGGGGCCCTTATATTTAAAAATTT TCAAAAGAAATTAG	NUH0017	*cps23FJ* 3’
O2818	AGGCAGTAAGTCATACAATTCTTC		
For construction of Δ*cps24AJ*::P-*erm*			
O2842	AAAGGTGCTTTATTAGCAGCAA	PATH2464	*cps24AJ* 5’
O2843	CCATTAAAAATCAAACGGATCCTACCTCACTGAACGTT GCTTTCCTATATTC		
O1	TAGGATCCGTTTGATTTTTAATGGATAATG	P-*erm* cassette	P-*erm*
O2	GGGCCCCTTTCCTTATGCTTTTG		
O2844	CAAAAGCATAAGGAAAGGGGCCCTTAAAGAGAATTATT AAAGAAATTAAATG	PATH2464	*cps24AJ* 3’
O2845	GGATATCAAACTCCAACATGCC		
For construction of Δ*cps24BJ*::P-*erm*			
O2846	TTCTCAAGCTTGTACTGAATCT	PATH2465	*cps24BJ* 5’
O2847	CCATTAAAAATCAAACGGATCCTAACTTATTGATTTTTC TTTCATGCTCATC		
O1	TAGGATCCGTTTGATTTTTAATGGATAATG	P-*erm* cassette	P-*erm*
O2	GGGCCCCTTTCCTTATGCTTTTG		
O2292	CAAAAGCATAAGGAAAGGGGCCCAATCAAATTTTTTAT AAGGATATTTCATG	PATH2465	*cps24BJ* 3’
O2293	GAACTTTCCCTCCATCTGCTAC		
For construction of Δ*cps24FJ*::P-*erm*			
O2846	TTCTCAAGCTTGTACTGAATCT	PATH20	*cps24FJ* 5’
O2848	CCATTAAAAATCAAACGGATCCTATTTACTTATTGATTTT TCTTTCATGCTC		
O1	TAGGATCCGTTTGATTTTTAATGGATAATG	P-erm cassette	P-*erm*
O2	GGGCCCCTTTCCTTATGCTTTTG		
O2292	CAAAAGCATAAGGAAAGGGGCCCAATCAAATTTTTTAT AAGGATATTTCATG	PATH20	*cps24FJ* 3’
O2845	GGATATCAAACTCCAACATGCC		
For construction of Δ*cps27J*::P-*erm*			
O2849	AAGAAGAGGCTTAGATCGTTT	PATH2467	*cps27J* 5’
O2850	CCATTAAAAATCAAACGGATCCTAGTTTTGATTACTTAT TTTTGTAGTACTC		
O1	TAGGATCCGTTTGATTTTTAATGGATAATG	P-*erm* cassette	P-*erm*
O2	GGGCCCCTTTCCTTATGCTTTTG		
O2851	CAAAAGCATAAGGAAAGGGGCCCCAGACAAAAATAAG ACACATAAAATTATAAG	PATH2467	*cps27J* 3’
O2852	GTCTGTTCAAGAATATGACGATAC		
For construction of Δ*cps28AJ*::P-*erm*			
O2853	CGTGATGATGGCAGATGAGAG	PATH9002	*cps28AJ* 5’

O2854	CCATTAAAAATCAAACGGATCCTAATTTAATATTTTTTTC ATTACAGATTCC		
O1	TAGGATCCGTTTGATTTTTAATGGATAATG	P-erm cassette	P-*erm*
O2	GGGCCCCTTTCCTTATGCTTTTG		
O2855	CAAAAGCATAAGGAAAGGGGCCCAGTTCGATAGTCAT GATAAAAAAACAGTG	PATH9002	*cps28AJ* 3'
O2856	TCCTTTACCCGACCAATTTCC		
For construction of Δ*cps28FJ::*P-*erm*			
O2853	CGTGATGATGGCAGATGAGAG	PATH382	*cps28FJ* 5'
O2854	CCATTAAAAATCAAACGGATCCTAATTTAATATTTTTTTC ATTACAGATTCC		
O1	TAGGATCCGTTTGATTTTTAATGGATAATG	P-*erm* cassette	P-*erm*
O2	GGGCCCCTTTCCTTATGCTTTTG		
O2855	CAAAAGCATAAGGAAAGGGGCCCAGTTCGATAGTCAT GATAAAAAAACAGTG	PATH382	*cps28FJ* 3'
O2856	TCCTTTACCCGACCAATTTCC		
For construction of Δ*cps29J::*P-*erm*			
O2059	TACGGAGTGAGAGTGTCCAA	PATH4478	*cps29J* 5'
O2060	CCATTAAAAATCAAACGGATCCTAGTTGTAGGCATAGT TTTTTAATACTTTC		
O1	TAGGATCCGTTTGATTTTTAATGGATAATG	P-*erm* cassette	P-*erm*
O2	GGGCCCCTTTCCTTATGCTTTTG		
O2061	CAAAAGCATAAGGAAAGGGGCCCCAACAATTATTAAAA AATAAAGGAGC	PATH4478	*cps29J* 3'
O2062	CTGCAAAGCTGCTCCAATTAC		
For construction of Δ*cps31J::*P-*erm*			
O2826	GTCTCTTTATCAAGTTCCTGGTTT	PATH18	*cps31J* 5'
O2827	CCATTAAAAATCAAACGGATCCTAATAATTTCGGGCAAT GGATTTCCTTTTC		
O1	TAGGATCCGTTTGATTTTTAATGGATAATG	P-*erm* cassette	P-*erm*
O2	GGGCCCCTTTCCTTATGCTTTTG		
O2828	CAAAAGCATAAGGAAAGGGGCCCATAGAAGTTAGAAA GAAAATATATAGATAG	PATH18	*cps31J* 3'
O2829	GGTACGCTAGATTTGTATCCAATG		
For construction of Δ*cps32AJ::*P-*erm*			
O2819	TTTGGCAGGTAATATAAGCGTT	PATH6653	*cps32AJ* 5'
O2820	CCATTAAAAATCAAACGGATCCTACTTTTCAACGCTCTT TTTTTGACTTTCC		
O1	TAGGATCCGTTTGATTTTTAATGGATAATG	P-*erm* cassette	P-*erm*
O2	GGGCCCCTTTCCTTATGCTTTTG		
O2821	CAAAAGCATAAGGAAAGGGGCCCAAACTAAATGTTCTT GCAAGGAAAGTATG	PATH6653	*cps32AJ* 3'
O2822	CAACAGTCTCTCAATCAACTACTATC		
For construction of Δ*cps32FJ::*P-*erm*			
O2819	TTTGGCAGGTAATATAAGCGTT	PATH2468	*cps32FJ* 5'
O2820	CCATTAAAAATCAAACGGATCCTACTTTTCAACGCTCTT TTTTTGACTTTCC		
O1	TAGGATCCGTTTGATTTTTAATGGATAATG	P-*erm* cassette	P-*erm*
O2	GGGCCCCTTTCCTTATGCTTTTG		
O2821	CAAAAGCATAAGGAAAGGGGCCCAAACTAAATGTTCTT GCAAGGAAAGTATG	PATH2468	*cps32FJ* 3'
O2822	CAACAGTCTCTCAATCAACTACTATC		
For construction of Δ*cps33AJ::*P-*erm*			
O2387	GATTTCTGAGACAAGTTTCGTCTAT	PATH1754	*cps33AJ* 5'

O2388	CCATTAAAAATCAAACGGATCCTAATTGTAGGCATAGTTTTTTAGTATTTTC		
O1	TAGGATCCGTTTGATTTTTAATGGATAATG	P-*erm* cassette	P-*erm*
O2	GGGCCCCTTTCCTTATGCTTTTG		
O2389	CAAAAGCATAAGGAAAGGGGCCCGAATTAAAACAAATAATCAGGAAAAATTAG	PATH1754	*cps33AJ* 3’
O2390	CACCGACAATAAGATAATCGTACATC		
For construction of Δ*cps33BJ::*P-*erm*			
O133	TCAATGGTCTGGTGGTCAATTA	PATH1945	*cps33BJ* 5’
O2391	CCATTAAAAATCAAACGGATCCTAATTGTAGGCGTAATTTTTTAGTACTTTC		
O1	TAGGATCCGTTTGATTTTTAATGGATAATG	P-*erm* cassette	P-*erm*
O2	GGGCCCCTTTCCTTATGCTTTTG		
O2385	CAAAAGCATAAGGAAAGGGGCCCGAATTAAAACAAATTATTAGGAAAAAC	PATH1945	*cps33BJ* 3’
O134	AAATCCCTACGCTTACAACTTT		
For construction of Δ*cps33CJ::*P-*erm*			
O1539	ATTTGCTGAATCTCCTATTTGGG	PATH344	*cps33CJ* 5’
O2898	CCATTAAAAATCAAACGGATCCTAATTATAAGCGTAGTTTTTTAGTACTTTC		
O1	TAGGATCCGTTTGATTTTTAATGGATAATG	P-*erm* cassette	P-*erm*
O2	GGGCCCCTTTCCTTATGCTTTTG		
O2899	CAAAAGCATAAGGAAAGGGGCCCAAAGAATTAAAACAAATCATTAAGAAATAA	PATH344	*cps33CJ* 3’
O1540	AGGAATCTCACGTTCTGTATAGTT		
For construction of Δ*cps33DJ::*P-*erm*			
O4012	TGTACACCCTACCTCAACAG	PATH2481	*cps33DJ* 5’
O2391	CCATTAAAAATCAAACGGATCCTAATTGTAGGCGTAATTTTTTAGTACTTTC		
O1	TAGGATCCGTTTGATTTTTAATGGATAATG	P-*erm* cassette	P-*erm*
O2	GGGCCCCTTTCCTTATGCTTTTG		
O2385	CAAAAGCATAAGGAAAGGGGCCCGAATTAAAACAAATTATTAGGAAAAAC	PATH2481	*cps33DJ* 3’
O4013	ATGGTAAGTGGTCTTTGCTATG		
For construction of Δ*cps33FJ::*P-*erm*			
O2387	GATTTCTGAGACAAGTTTCGTCTAT	PATH101	*cps33FJ* 5’
O2388	CCATTAAAAATCAAACGGATCCTAATTGTAGGCATAGTTTTTTAGTATTTTC		
O1	TAGGATCCGTTTGATTTTTAATGGATAATG	P-*erm* cassette	P-*erm*
O2	GGGCCCCTTTCCTTATGCTTTTG		
O2389	CAAAAGCATAAGGAAAGGGGCCCGAATTAAAACAAATAATCAGGAAAAATTAG	PATH101	*cps33FJ* 3’
O2390	CACCGACAATAAGATAATCGTACATC		
For construction of Δ*cps34J::*P-*erm*			
O1523	CGCGGGATTAAAGAAGCTAATG	CCUG2399	*cps34J* 5’
O2392	CCATTAAAAATCAAACGGATCCTAATTATAGGCGTAGTTTTTTAGTACTTTC		
O1	TAGGATCCGTTTGATTTTTAATGGATAATG	P-*erm* cassette	P-*erm*
O2	GGGCCCCTTTCCTTATGCTTTTG		
O2393	CAAAAGCATAAGGAAAGGGGCCCAAAGAATTAAAACATATTATTAAAAAATAA	CCUG2399	*cps34J* 3’
O1526	GCGAAGGCTACGGTATTCTAA		
For construction of Δ*cps35AJ::*P-*erm*			
O2857	ACAGGTTTAGTAGGCCTTCAATTA	PATH1709	*cps35AJ* 5’

O2858	CCATTAAAAATCAAACGGATCCTAATTATATAAGTAGTT TTTTAATACCTTC		
O1	TAGGATCCGTTTGATTTTTAATGGATAATG	P-*erm* cassette	P-*erm*
O2	GGGCCCCTTTCCTTATGCTTTTG		
O2385	CAAAAGCATAAGGAAAGGGGCCCGAATTAAAACAAATT ATTAGGAAAAACTAG	PATH1709	*cps35AJ* 3’
O2859	GTGGTCACGTTTATCAATCACTTT		
For construction of Δ*cps35BJ::*P-*erm*			
O2217	ACTATGGAGTTGCTCACCAATTA	PATH51	*cps35BJ* 5’
O2218	CCATTAAAAATCAAACGGATCCTAATTGTATGCATAGTT TTTAAGTACTTTC		
O1	TAGGATCCGTTTGATTTTTAATGGATAATG	P-*erm* cassette	P-*erm*
O2	GGGCCCCTTTCCTTATGCTTTTG		
O2219	CAAAAGCATAAGGAAAGGGGCCCGAATTAAAACAAGTA ATTAGGAAAAATTAG	PATH51	*cps35BJ* 3’
O2220	GAATTCCTTCCTCTTCACGAGTAT		
For construction of Δ*cps35CJ::*P-*erm*			
O2857	ACAGGTTTAGTAGGCCTTCAATTA	PATH1895	*cps35CJ* 5’
O2858	CCATTAAAAATCAAACGGATCCTAATTATATAAGTAGTT TTTTAATACCTTC		
O1	TAGGATCCGTTTGATTTTTAATGGATAATG	P-*erm* cassette	P-*erm*
O2	GGGCCCCTTTCCTTATGCTTTTG		
O2385	CAAAAGCATAAGGAAAGGGGCCCGAATTAAAACAAATT ATTAGGAAAAACTAG	PATH1895	*cps35CJ* 3’
O2860	CAAACCAGCACCGACAATAAG		
For construction of Δ*cps36J::*P-*erm*			
O2900	GGAACAGCTTGTCAACAGATTG	PATH1833	*cps36J* 5’
O2901	CCATTAAAAATCAAACGGATCCTAATTATATGCATAATT TTTTAAAACCTTC		
O1	TAGGATCCGTTTGATTTTTAATGGATAATG	P-*erm* cassette	P-*erm*
O2	GGGCCCCTTTCCTTATGCTTTTG		
O2902	CAAAAGCATAAGGAAAGGGGCCCGAGATAAAAGAGCA GCTGTTAAATAATTAG	PATH1833	*cps36J* 3’
O2903	GCAGTACAAGAGGTATAACACCATA		
For construction of Δ*cps39J::*P-*erm*			
O552	AGTCAGGCGTATTCTTCACAAG	PATH2009	*cps39J* 5’
O2063	CCATTAAAAATCAAACGGATCCTAGTTATACGCGTAATT TTTTAGTACTTTC		
O1	TAGGATCCGTTTGATTTTTAATGGATAATG	P-*erm* cassette	P-*erm*
O2	GGGCCCCTTTCCTTATGCTTTTG		
O2061	CAAAAGCATAAGGAAAGGGGCCCCAACAATTATTAAAA AATAAAGGAGCTTAA	PATH2009	*cps39J* 3’
O555	TAAGGTTCATCTCCGCGTTTC		
For construction of Δ*cps40J::*P-*erm*			
O2290	GTGACTGTGAGTCCTAACGATATT	PATH2469	*cps40J* 5’
O2296	CCATTAAAAATCAAACGGATCCTAATTTATTGATTTTTCT TTCATGCTCATC		
O1	TAGGATCCGTTTGATTTTTAATGGATAATG	P-*erm* cassette	P-*erm*
O2	GGGCCCCTTTCCTTATGCTTTTG		
O2292	CAAAAGCATAAGGAAAGGGGCCCAATCAAATTTTTTAT AAGGATATTTCATG	PATH2469	*cps40J* 3’
O2293	GAACTTTCCCTCCATCTGCTAC		
For construction of Δ*cps41AJ::*P-*erm*			
O2823	ACCGTCTCTTTATCAAGTTCCT	PATH2471	*cps41AJ* 5’

O2824	CCATTAAAAATCAAACGGATCCTATCGGGCAATGGATT TCCTTTTCACAATC		
O1	TAGGATCCGTTTGATTTTTAATGGATAATG	P-*erm* cassette	P-*erm*
O2	GGGCCCCTTTCCTTATGCTTTTG		
O2828	CAAAAGCATAAGGAAAGGGGCCCATAGAAGTTAGAAA GAAAATATATAGATAG	PATH2471	*cps41AJ* 3'
O2825	CTGATTCTACAGCAGATGTATCAAA		
For construction of Δ*cps41FJ::*P-*erm*			
O2823	ACCGTCTCTTTATCAAGTTCCT	PATH2470	*cps41FJ* 5'
O2827	CCATTAAAAATCAAACGGATCCTAATAATTTCGGGCAAT GGATTTCCTTTTC		
O1	TAGGATCCGTTTGATTTTTAATGGATAATG	P-*erm* cassette	P-*erm*
O2	GGGCCCCTTTCCTTATGCTTTTG		
O2861	CAAAAGCATAAGGAAAGGGGCCCATAGAAGTCAGAAA GAAAATATATAGATAA	PATH2470	*cps41FJ* 3'
O2862	AGGATTAGTCTCTAACACCGTTTC		
For construction of Δ*cps42J::*P-*erm*			
O2863	ACAGGTTTAGTAGGCCTTCAATTA	CCUG6568	*cps42J* 5'
O2858	CCATTAAAAATCAAACGGATCCTAATTATATAAGTAGTT TTTTAATACCTTC		
O1	TAGGATCCGTTTGATTTTTAATGGATAATG	P-*erm* cassette	P-*erm*
O2	GGGCCCCTTTCCTTATGCTTTTG		
O2385	CAAAAGCATAAGGAAAGGGGCCCGAATTAAAACAAATT ATTAGGAAAAACTAG	CCUG6568	*cps42J* 3'
O2864	CAAACCAGCACCGACAATAAG		
For construction of Δ*cps43J::*P-*erm*			
O2904	AGAGTCATCTGACCAAAGGAATTTA	PATH2472	*cps43J* 5'
O2905	CCATTAAAAATCAAACGGATCCTACCTGATAGATTTAAC TTTATAACTTGTC		
O1	TAGGATCCGTTTGATTTTTAATGGATAATG	P-*erm* cassette	P-*erm*
O2	GGGCCCCTTTCCTTATGCTTTTG		
O2906	CAAAAGCATAAGGAAAGGGGCCCATTTTCTATAAGAAA TTAAAGAAAATTTAAC	PATH2472	*cps43J* 3'
O2907	GCAAGACGACCACAGAAGATAA		
For construction of Δ*cps44J::*P-*erm*			
O2297	ACAAGTATGCCCAATTTGTCT	PATH2473	*cps44J* 5'
O2298	CCATTAAAAATCAAACGGATCCTAATTAAAAAAACTATT TTTTACACGACTC		
O1	TAGGATCCGTTTGATTTTTAATGGATAATG	P-*erm* cassette	P-*erm*
O2	GGGCCCCTTTCCTTATGCTTTTG		
O2299	CAAAAGCATAAGGAAAGGGGCCCAAAAATTTGGAGGA AAAAAATCATGATTAATG	PATH2473	*cps44J* 3'
O2300	TAGCAGGTGTTGCCAGAT		
For construction of Δ*cps45J::*P-*erm*			
O2908	ATGAGGATATCTGTTGCGATGA	PATH656	*cps45J* 5'
O2909	CCATTAAAAATCAAACGGATCCTAATATAGATAATTTTT AGAAATTTTGCTC		
O1	TAGGATCCGTTTGATTTTTAATGGATAATG	P-*erm* cassette	P-*erm*
O2	GGGCCCCTTTCCTTATGCTTTTG		
O2910	CAAAAGCATAAGGAAAGGGGCCCGTAAAGAAAGGATT CAAAAAAAGTAGATG	PATH656	*cps45J* 3'
O2911	GTTGCATAAATGGTTGTAAAGCTG		
For construction of Δ*cps46J::*P-*erm*			
O2297	ACAAGTATGCCCAATTTGTCT	PATH2474	*cps46J* 5'

O2298	CCATTAAAAATCAAACGGATCCTAATTAAAAAAACTATT TTTTACACGACTC		
O1	TAGGATCCGTTTGATTTTTAATGGATAATG	P-*erm* cassette	P-*erm*
O2	GGGCCCCTTTCCTTATGCTTTTG		
O2299	CAAAAGCATAAGGAAAGGGGCCCAAAAATTTGGAGGA AAAAAATCATGATTAATG	PATH2474	*cps46J* 3’
O2300	TAGCAGGTGTTGCCAGAT		
For construction of Δ*cps47AJ::*P-*erm*			
O560	GGATTCACGCATTGTAATTATAGAC	PATH2476	*cps47AJ* 5’
O2657	CCATTAAAAATCAAACGGATCCTAATTATATAAAAAATT TTTCAAAACTTTC		
O1	TAGGATCCGTTTGATTTTTAATGGATAATG	P-*erm* cassette	P-*erm*
O2	GGGCCCCTTTCCTTATGCTTTTG		
O2658	CAAAAGCATAAGGAAAGGGGCCCGAGATTTCTATGATA AGAAAAACCCCC	PATH2476	*cps47AJ* 3’
O563	GGTATTCTAACTCTCCGTGCT		
For construction of Δ*cps47FJ::*P-*erm*			
O564	CGCGGGATTAAAGAAGCTAGT	PATH2475	*cps47FJ* 5’
O2659	CCATTAAAAATCAAACGGATCCTATTTAAGTACTTTCAT ACAAGCACACTCC		
O1	TAGGATCCGTTTGATTTTTAATGGATAATG	P-*erm* cassette	P-*erm*
O2	GGGCCCCTTTCCTTATGCTTTTG		
O2219	CAAAAGCATAAGGAAAGGGGCCCGAATTAAAACAAGTA ATTAGGAAAAATTAG	PATH2475	*cps47FJ* 3’
O567	ATTTGACGATTCGATTGAAGTAAGG		
For construction of Δ*cps48J::*P-*erm*			
O2912	TCAACATACCTTTCAGATCTTAGCC	PATH1937	*cps48J* 5’
O2913	CCATTAAAAATCAAACGGATCCTAATTATATATGTAGTT TTTTAATAATTTC		
O1	TAGGATCCGTTTGATTTTTAATGGATAATG	P-*erm* cassette	P-*erm*
O2	GGGCCCCTTTCCTTATGCTTTTG		
O2914	CAAAAGCATAAGGAAAGGGGCCCACTATACTTAAAAAA ATAAAGATACAGTAG	PATH1937	*cps48J* 3’
O2915	TAGTTTGAACGAGGTTGCTCTG		
**For construction of deletion of GTs**			
For construction of Δ*cps5(whaD)::*P-*erm*			
O2704	GTCAATGATGGATCTACAG	PATH46	*cps5 (whaD)* 5’
O2705	CATTATCCATTAAAAATCAAACGGATCCTATAACATAAC CCTAAAAGGAGC		
O1	TAGGATCCGTTTGATTTTTAATGGATAATG	P-*erm* cassette	P-*erm*
O2	GGGCCCCTTTCCTTATGCTTTTG		
N143	CAAAAGCATAAGGAAAGGGGCCCTATAATCTGGAAGG TTCAGCAAAAAATATTATG	PATH46	*cps5 (whaD)* 3’
O2707	CGCACTACAATATTTTTCTGAC		
For construction of Δ*cps7A(wcwH)::*P-*erm*			
O2865	CTGGATGCAAACATAGTTC	PATH2477	*cps7A (wcwH)* 5’
N439	CATTATCCATTAAAAATCAAACGGATCCTAAATTTTATAT CCTCCCGTAGGAACAGTGC		
O1	TAGGATCCGTTTGATTTTTAATGGATAATG	P-*erm* cassette	P-*erm*
O2	GGGCCCCTTTCCTTATGCTTTTG		
N144	CGTCCAAAAGCATAAGGAAAGGGGCCCGGATGGGAGA AAACTACTTTAAAATTAG	PATH2477	*cps7A (wcwH)* 3’
O2868	CCTCTCCTAAACCAATACC		

For construction of Δ*cps18B(wciY)::*P-*erm*			
O3099	GGTGTATTTATGAAGACAGA	PATH269	*cps18B (wciY)* 5’
N415	CATTATCCATTAAAAATCAAACGGATCCTACAATAAATGCAACGGTAATGTAAAGAC		
O1	TAGGATCCGTTTGATTTTTAATGGATAATG	P-*erm* cassette	P-*erm*
O2	GGGCCCCTTTCCTTATGCTTTTG		
N416	CGTCCAAAAGCATAAGGAAAGGGGCCCTCAGTACAGTGTGCTAATTGGATTGAAG	PATH269	*cps18B (wciY)* 3’
O3102	GGTCAATAAAACCCAGTTTG		
For construction of Δ*cps19C(wchR)::*P-*erm*			
O3311	CGGAGAAAACTATCTTCACG	PATH2463	*cps19C (wchR)* 5’
N440	CATTATCCATTAAAAATCAAACGGATCCTATGGACGTTGTTTTATCCAATTCCAATC		
O1	TAGGATCCGTTTGATTTTTAATGGATAATG	P-*erm* cassette	P-*erm*
O2	GGGCCCCTTTCCTTATGCTTTTG		
O3313	CGTCCAAAAGCATAAGGAAAGGGGCCCAAAAGTAATACTTGGGAAAAAAG	PATH2463	*cps19C (wchR)* 3’
O3314	CCAAATTGAACGAATAACTC		
For construction of Δ*cps23F(wchX)::*P-*erm*			
N417	CAGTTATGCTTGCCATGAATGT	NUH0017	*cps23F (wchX)* 5’
N418	CATTATCCATTAAAAATCAAACGGATCCTAACTTATCAAAAACATAAAAACGAATC		
O1	TAGGATCCGTTTGATTTTTAATGGATAATG	P-*erm* cassette	P-*erm*
O2	GGGCCCCTTTCCTTATGCTTTTG		
N419	CGTCCAAAAGCATAAGGAAAGGGGCCCCCAATCTTTAATGGAACCGCTAGTGAAAATG	NUH0017	*cps23F (wchX)* 3’
N420	TTCATGCAGATAGGTATAGCGATTA		
For construction of Δ*cps27(whaL)::*P-*erm*			
O3330	GGAGTAGGGATGTTACCT	PATH2467	*cps27 (whaL)* 5’
N496	CATTATCCATTAAAAATCAAACGGATCCTAATACAAAAATAGATCGTCACCTAAATTAAC		
O1	TAGGATCCGTTTGATTTTTAATGGATAATG	P-*erm* cassette	P-*erm*
O2	GGGCCCCTTTCCTTATGCTTTTG		
N149	CTAAACGTCCAAAAGCATAAGGAAAGGGGCCCCTAGATGAACGACTAATGAGAGGCAC	PATH2467	*cps27 (whaL)* 3’
O3333	GTGTAAATTTCCCACCAAC		
For construction of Δ*cps27(wcrN)::*P-*erm*			
O1027	GTGAGTATTTTTGCCACATTGAT	PATH2467	*cps27 (wcrN)* 5’
N443	CATTATCCATTAAAAATCAAACGGATCCTAAATCGTATGTAAAATCTCCAACGATAC		
O1	TAGGATCCGTTTGATTTTTAATGGATAATG	P-*erm* cassette	P-*erm*
O2	GGGCCCCTTTCCTTATGCTTTTG		
N150	CTAAACGTCCAAAAGCATAAGGAAAGGGGCCCATTTTACCTCCTGAAAAGGATAGGG	PATH2467	*cps27 (wcrN)* 3’
O2879	CATAAACCGGCATAAGTTG		
For construction of Δ*cps32F(wcrN)::*P-*erm*			
O288	GTGAGTATTTTTTCAACATTGA	PATH2468	*cps32F (wcrN)* 5’
O5249	CATTATCCATTAAAAATCAAACGGATCCTAAATCGTGTGTAAAATCTCCAACGATAC		
O1	TAGGATCCGTTTGATTTTTAATGGATAATG	P-*erm* cassette	P-*erm*
O2	GGGCCCCTTTCCTTATGCTTTTG		
O5250	CGTCCAAAAGCATAAGGAAAGGGGCCCATTTTGCCTCCAGAGAAGGATAGAGTAGG	PATH2468	*cps32F (wcrN)* 3’
O2879	CATAAACCGGCATAAGTTG		

For construction of Δ*cps32F(wchQ)::*P-*erm*			
O3340	GGTTTTGCTACATATGGATC	PATH2468	*cps32F (wchQ)* 5’
O5247	CCATTAAAAATCAAACGGATCCTAATTGACGCATTGTTT<br>AGTTTCTTTAAAAACC		
O1	TAGGATCCGTTTGATTTTTAATGGATAATG	P-*erm* cassette	P-*erm*
O2	GGGCCCCTTTCCTTATGCTTTTG		
O5248	CGTCCAAAAGCATAAGGAAAGGGGCCCTGTCTACTGC<br>AGTCAACAAAAGCG	PATH2468	*cps32F (wchQ)* 3’
O3344	GACTTTTCAAATCCTACACC		
For construction of Δ*cps33F(wciF)::*P-*erm*			
O2788	CATTTACTCCTGCTATTGCTC	PATH101	*cps33F (wciF)* 5’
N413	CATTATCCATTAAAAATCAAACGGATCCTAATCCAACGC<br>AAAGCGCAGATAATCCGCG		
O1	TAGGATCCGTTTGATTTTTAATGGATAATG	P-*erm* cassette	P-*erm*
O2	GGGCCCCTTTCCTTATGCTTTTG		
N414	CGTCCAAAAGCATAAGGAAAGGGGCCCCTTTCCCCAA<br>ATTACTACTATAAAATAAAAG	PATH101	*cps33F (wciF)* 3’
O2791	CTAAGACCGTCTGAAATACC		
For construction of Δ*cps35C(wcrK)::*P-*erm*			
O3195	CAGGAGATGCTTATAGTTGC	PATH1895	*cps35C (wcrK)* 5’
N151	CCATTAAAAATCAAACGGATCCTAAGCACGTAAAACCA<br>CATTTTTCACACCATATC		
O1	TAGGATCCGTTTGATTTTTAATGGATAATG	P-*erm* cassette	P-*erm*
O2	GGGCCCCTTTCCTTATGCTTTTG		
N152 OL F	CTAAACGTCCAAAAGCATAAGGAAAGGGGCCCTTAGAT<br>AATGTTGCTGAAGATAG	PATH1895	*cps35C (wcrK)* 3’
O3198	CATCACCCTTACCAACAAC		
For construction of Δ*cps41A(wcrW)::*P-*erm*			
O2881	CATTAGGACAATTTAATCCG	PATH2471	*cps41A (wcrW)* 5’
N497	CATTATCCATTAAAAATCAAACGGATCCTAATGAAAAAA<br>ATTTTTTTCTATTGTTCG		
O1	TAGGATCCGTTTGATTTTTAATGGATAATG	P-*erm* cassette	P-*erm*
O2	GGGCCCCTTTCCTTATGCTTTTG		
N447	CGTCCAAAAGCATAAGGAAAGGGGCCCCTTGATACAAT<br>TGTAGATGAAATTGCGGGAC	PATH2471	*ps41A (wcrW)* 3’
O2884	GAAACAAAAGTACTCCCAC		
For construction of Δ*cps41A(wcrQ)::*P-*erm*			
O3345	GCGAACTCAGATATATGTGG	PATH2471	*cps41A (wcrQ)* 5’
N153	CCATTAAAAATCAAACGGATCCTAAGGTATTCGTTTTGT<br>TACTTTATAAATGATAC		
O1	TAGGATCCGTTTGATTTTTAATGGATAATG	P-*erm* cassette	P-*erm*
O2	GGGCCCCTTTCCTTATGCTTTTG		
N154	CCAAAAGCATAAGGAAAGGGGCCCAAAAATGGTAGGG<br>AAACGGTTTATAAAAAAATTG	PATH2471	*cps41A (wcrQ)* 3’
O3348	GCTATTCTTATACCAAGCAC		
**Illumina primers for next-generation sequencing**			
F1	AATGATACGGCGACCACCGAGATCTACACTCTTTCCCTACACGACGCTCTTCCGATC<br>T*GGAACTCCTACTGAC*GTTCTAAATACGGTACTAAACGTCC		
F2	AATGATACGGCGACCACCGAGATCTACACTCTTTCCCTACACGACGCTCTTCCGATC<br>T*TAGCACATGCTGACT*GTTCTAAATACGGTACTAAACGTCC		
F3	AATGATACGGCGACCACCGAGATCTACACTCTTTCCCTACACGACGCTCTTCCGATC<br>T*CCTTGAGGATGACTG*GTTCTAAATACGGTACTAAACGTCC		
F4	AATGATACGGCGACCACCGAGATCTACACTCTTTCCCTACACGACGCTCTTCCGATC<br>T*ATCGTGTACGACTGA*GTTCTAAATACGGTACTAAACGTCC		

F5	AATGATACGGCGACCACCGAGATCTACACTCTTTCCCTACACGACGCTCTTCCGATCT*AGACCTATGTGATGA*GTTCTAAATACGGTACTAAACGTCC
F6	AATGATACGGCGACCACCGAGATCTACACTCTTTCCCTACACGACGCTCTTCCGATCT*TCTGGATACACTACT*GTTCTAAATACGGTACTAAACGTCC
F7	AATGATACGGCGACCACCGAGATCTACACTCTTTCCCTACACGACGCTCTTCCGATCT*CTGATGGGAGACCTG*GTTCTAAATACGGTACTAAACGTCC
R1	CAAGCAGAAGACGGCATACGAGATATTACTCGGTGACTGGAGTTCAGACGTGTGCTCTTCCGATCTTATGACCTATAATGAAAAGCGACGG
R2	CAAGCAGAAGACGGCATACGAGATTCCGGAGAGTGACTGGAGTTCAGACGTGTGCTCTTCCGATCTTATGACCTATAATGAAAAGCGACGG
R3	CAAGCAGAAGACGGCATACGAGATCGCTCATTGTGACTGGAGTTCAGACGTGTGCTCTTCCGATCTTATGACCTATAATGAAAAGCGACGG
R4	CAAGCAGAAGACGGCATACGAGATGAGATTCCGTGACTGGAGTTCAGACGTGTGCTCTTCCGATCTTATGACCTATAATGAAAAGCGACGG

Data S1. CpsJ flippases interchangeability dataset (separate file)

(A) Values of the Tanimoto coefficient of CPS substrates (see Fig. 1A)
(B) Values of Root Mean Square Deviation (RMSD) of CpsJ flippases (see Fig. 1B)
(C) Library sizes of the "Input" and "Output" libraries (refer to Fig. 2)
(D) Bar-seq data on the ratio of NGS read counts after *cpsJ* deletion (refer to Fig. 3)
(E) Fold-changes in relative barcode abundance (refer to Fig. S6)

REFERENCES AND NOTES

1. N. Ruiz, Bioinformatics identification of MurJ (MviN) as the peptidoglycan lipid II flippase in *Escherichia coli*. *Proc. Natl. Acad. Sci. U.S.A.* **105**, 15553–15557 (2008).

2. L.-T. Sham, E. K. Butler, M. D. Lebar, D. Kahne, T. G. Bernhardt, N. Ruiz, Bacterial cell wall. MurJ is the flippase of lipid-linked precursors for peptidoglycan biogenesis. *Science* **345**, 220–222 (2014).

3. N. Ruiz, Lipid flippases for bacterial peptidoglycan biosynthesis. *Lipid Insights* **8s1**, LPI.S31783 (2015).

4. A. C. Y. Kuk, A. Hao, S.-Y. Lee, Structure and mechanism of the lipid flippase MurJ. *Annu. Rev. Biochem.* **91**, 705–729 (2022).

5. M. F. Feldman, C. L. Marolda, M. A. Monteiro, M. B. Perry, A. J. Parodi, M. A. Valvano, The activity of a putative polyisoprenol-linked sugar translocase (Wzx) involved in *Escherichia coli* O antigen assembly Is independent of the chemical structure of the O repeat. *J. Biol. Chem.* **274**, 35129–35138 (1999).

6. Y. Hong, M. A. Liu, P. R. Reeves, Progress in our understanding of Wzx flippase for translocation of bacterial membrane lipid-linked oligosaccharide. *J. Bacteriol.* **200,** e00154-17 (2018).

7. J. Helenius, D. T. W. Ng, C. L. Marolda, P. Walter, M. A. Valvano, M. Aebi, Translocation of lipid-linked oligosaccharides across the ER membrane requires Rft1 protein. *Nature* **415**, 447–450 (2002).

8. R. N. Hvorup, B. Winnen, A. B. Chang, Y. Jiang, X.-F. Zhou, M. H. Saier, The multidrug/oligosaccharidyl-lipid/polysaccharide (MOP) exporter superfamily: The MOP superfamily. *Eur. J. Biochem.* **270**, 799–813 (2003).

9. T. Kuroda, T. Tsuchiya, Multidrug efflux transporters in the MATE family. *Biochim. Biophys. Acta* **1794**, 763–768 (2009).

10. T. Su, R. Nakamoto, Y.-Y. Chun, W.-Z. Chua, J.-H. Chen, J. J. Zik, L.-T. Sham, Decoding capsule synthesis in *Streptococcus pneumoniae*. *FEMS Microbiol. Rev.* **45**, fuaa067 (2021).

11. J. Yother, Capsules of *Streptococcus pneumoniae* and other bacteria: Paradigms for polysaccharide biosynthesis and regulation. *Annu. Rev. Microbiol.* **65**, 563–581 (2011).

12. K. A. Geno, G. L. Gilbert, J. Y. Song, I. C. Skovsted, K. P. Klugman, C. Jones, H. B. Konradsen, M. H. Nahm, Pneumococcal capsules and their types: Past, present, and future. *Clin. Microbiol. Rev.* **28**, 871–899 (2015).

13. C. Hyams, S. Opel, W. Hanage, J. Yuste, K. Bax, B. Henriques-Normark, B. G. Spratt, J. S. Brown, Effects of *Streptococcus pneumoniae* strain background on complement resistance. *PLOS ONE* **6**, e24581 (2011).

14. M. Abeyta, G. G. Hardy, J. Yother, Genetic alteration of capsule type but not PspA type affects accessibility of surface-bound complement and surface antigens of *Streptococcus pneumoniae*. *Infect. Immun.* **71**, 218–225 (2003).

15. J. N. Weiser, D. M. Ferreira, J. C. Paton, *Streptococcus pneumoniae*: Transmission, colonization and invasion. *Nat. Rev. Microbiol.* **16**, 355–367 (2018).

16. F. Ganaie, K. Maruhn, C. Li, R. J. Porambo, P. L. Elverdal, C. Abeygunwardana, M. Van Der Linden, J. Ø. Duus, C. L. Sheppard, M. H. Nahm, Structural, genetic, and serological elucidation of *Streptococcus pneumoniae* serogroup 24 serotypes: Discovery of a new serotype, 24C, with a variable capsule structure. *J. Clin. Microbiol.* **59**, e0054021 (2021).

17. C. Li, K. A. Duda, P. L. Elverdal, I. C. Skovsted, C. Kjeldsen, D. Teze, J. Ø. Duus, Structural, biosynthetic and serological cross-reactive elucidation of capsular polysaccharides from *Streptococcus pneumoniae* serogroup 28. *Carbohydr. Polym.* **254**, 117323 (2021).

18. F. A. Ganaie, J. S. Saad, S. W. Lo, L. McGee, A. J. Van Tonder, P. A. Hawkins, J. J. Calix, S. D. Bentley, M. H. Nahm, Novel pneumococcal capsule type 33E results from the inactivation of glycosyltransferase WciE in vaccine type 33F. *J. Biol. Chem.* **299**, 105085 (2023).

19. T. R. Larson, J. Yother, *Streptococcus pneumoniae* capsular polysaccharide is linked to peptidoglycan via a direct glycosidic bond to β-D-*N*-acetylglucosamine. *Proc. Natl. Acad. Sci. U.S.A.* **114**, 5695–5700 (2017).

20. B. Xayarath, J. Yother, Mutations blocking side chain assembly, polymerization, or transport of a Wzy-dependent *Streptococcus pneumoniae* capsule are lethal in the absence of suppressor mutations and can affect polymer transfer to the cell wall. *J. Bacteriol.* **189**, 3369–3381 (2007).

21. W.-Z. Chua, M. Maiwald, K. L. Chew, R. T.-P. Lin, S. Zheng, L.-T. Sham, High-throughput mutagenesis and cross-complementation experiments reveal substrate preference and critical residues of the capsule transporters in *Streptococcus pneumoniae*. *MBio* **12**, e0261521 (2021).

22. Y. Hong, M. M. Cunneen, P. R. Reeves, The Wzx translocases for *Salmonella enterica* O-antigen processing have unexpected serotype specificity: Wzx specificity in *Salmonella enterica*. *Mol. Microbiol.* **84**, 620–630 (2012).

23. M. A. Liu, P. Morris, P. R. Reeves, Wzx flippases exhibiting complex O-unit preferences require a new model for Wzx–substrate interactions. *MicrobiologyOpen* **8**, e00655 (2019).

24. S. Kumar, F. A. Rubino, A. G. Mendoza, N. Ruiz, The bacterial lipid II flippase MurJ functions by an alternating-access mechanism. *J. Biol. Chem.* **294**, 981–990 (2019).

25. A. C. Y. Kuk, A. Hao, Z. Guan, S.-Y. Lee, Visualizing conformation transitions of the Lipid II flippase MurJ. *Nat. Commun.* **10**, 1736 (2019).

26. F. A. Rubino, A. Mollo, S. Kumar, E. K. Butler, N. Ruiz, S. Walker, D. E. Kahne, Detection of transport intermediates in the peptidoglycan flippase MurJ identifies residues essential for conformational cycling. *J. Am. Chem. Soc.* **142**, 5482–5486 (2020).

27. M. Damjanovic, A. S. Kharat, A. Eberhardt, A. Tomasz, W. Vollmer, The essential *tacF* gene is responsible for the choline-dependent growth phenotype of *Streptococcus pneumoniae*. *J. Bacteriol.* **189**, 7105–7111 (2007).

28. L.-T. Sham, S. Zheng, A. A. Yakhnina, A. C. Kruse, T. G. Bernhardt, Loss of specificity variants of WzxC suggest that substrate recognition is coupled with transporter opening in MOP-family flippases. *Mol. Microbiol.* **109**, 633–641 (2018).

29. Y.-Y. Chun, K. S. Tan, L. Yu, M. Pang, M. H. M. Wong, R. Nakamoto, W.-Z. Chua, A. Huee-Ping Wong, Z. Z. R. Lew, H. H. Ong, V. T. Chow, T. Tran, D. Yun Wang, L.-T. Sham, Influence of glycan structure on the colonization of *Streptococcus pneumoniae* on human respiratory epithelial cells. *Proc. Natl. Acad. Sci. U.S.A.* **120**, e2213584120 (2023).

30. T. Su, W.-Z. Chua, Y. Liu, J. Fan, S.-Y. Tan, D. Yang, L.-T. Sham, Rewiring the pneumococcal capsule pathway for investigating glycosyltransferase specificity and genetic glycoengineering. *Sci. Adv.* **9**, eadi8157 (2023).

31. S. Kumar, A. Mollo, D. Kahne, N. Ruiz, The bacterial cell wall: From lipid II flipping to polymerization. *Chem. Rev.* **122**, 8884–8910 (2022).

32. S. Zheng, L.-T. Sham, F. A. Rubino, K. P. Brock, W. P. Robins, J. J. Mekalanos, D. S. Marks, T. G. Bernhardt, A. C. Kruse, Structure and mutagenic analysis of the lipid II flippase MurJ from *Escherichia coli*. *Proc. Natl. Acad. Sci. U.S.A.* **115**, 6709–6714 (2018).

33. H. An, C. Qian, Y. Huang, J. Li, X. Tian, J. Feng, J. Hu, Y. Fang, F. Jiao, Y. Zeng, X. Huang, X. Meng, X. Liu, X. Lin, Z. Zeng, M. Guilliams, A. Beschin, Y. Chen, Y. Wu, J. Wang, M. R. Oggioni, J. Leong, J.-W. Veening, H. Deng, R. Zhang, H. Wang, J. Wu, Y. Cui, J.-R. Zhang, Functional vulnerability of liver macrophages to capsules defines virulence of blood-borne bacteria. *J. Exp. Med.* **219**, e20212032 (2022).

34. I. Hug, M. R. Couturier, M. M. Rooker, D. E. Taylor, M. Stein, M. F. Feldman, *Helicobacter pylori* lipopolysaccharide is synthesized via a novel pathway with an evolutionary connection to protein N-glycosylation. *PLOS Pathog.* **6**, e1000819 (2010).

35. W. Elhenawy, R. M. Davis, J. Fero, N. R. Salama, M. F. Felman, N. Ruiz, The O-antigen flippase Wzk can substitute for MurJ in peptidoglycan synthesis in *Helicobacter pylori* and *Escherichia coli*. *PLOS ONE* **11**, e0161587 (2016).

36. S. D. Liston, E. Mann, C. Whitfield, Glycolipid substrates for ABC transporters required for the assembly of bacterial cell-envelope and cell-surface glycoconjugates. *Biochim. Biophys. Acta Mol. Cell Biol. Lipids* **1862**, 1394–1403 (2017).

37. C. L. Marolda, Wzx proteins involved in biosynthesis of O antigen function in association with the first sugar of the O-specific lipopolysaccharide subunit. *Microbiology* **150**, 4095–4105 (2004).

38. M. Rausch, J. P. Deisinger, H. Ulm, A. Müller, W. Li, P. Hardt, X. Wang, X. Li, M. Sylvester, M. Engeser, W. Vollmer, C. E. Müller, H. G. Sahl, J. C. Lee, T. Schneider, Coordination of capsule assembly and cell wall biosynthesis in *Staphylococcus aureus*. *Nat. Commun.* **10**, 1404 (2019).

39. L. J. Sweetlove, A. R. Fernie, The role of dynamic enzyme assemblies and substrate channelling in metabolic regulation. *Nat. Commun.* **9**, 2136 (2018).

40. R. Nakamoto, S. Bamyaci, K. Blomqvist, S. Normark, B. Henriques-Normark, L.-T. Sham, The divisome but not the elongasome organizes capsule synthesis in *Streptococcus pneumoniae*. *Nat. Commun.* **14**, 3170 (2023).

41. F. E. Jacobsen, K. M. Kazmierczak, J. P. Lisher, M. E. Winkler, D. P. Giedroc, Interplay between manganese and zinc homeostasis in the human pathogen *Streptococcus pneumoniae*. *Metallomics* **3**, 38–41 (2011).

42. J. E. Martin, J. P. Lisher, M. E. Winkler, D. P. Giedroc, Perturbation of manganese metabolism disrupts cell division in *Streptococcus pneumoniae*: Role for manganese efflux in *Streptococcus pneumoniae*. *Mol. Microbiol.* **104**, 334–348 (2017).

43. R. Junges, R. Khan, Y. Tovpeko, H. A. Åmdal, F. C. Petersen, D. A. Morrison, Markerless genome editing in competent Streptococci. *Methods Mol. Biol.* **1537**, 233–247 (2017).

44. D. G. Gibson, L. Young, R.-Y. Chuang, J. C. Venter, C. A. Hutchison, H. O. Smith, Enzymatic assembly of DNA molecules up to several hundred kilobases. *Nat. Methods* **6**, 343–345 (2009).

45. S. Guiral, V. Hénard, M.-H. Laaberki, C. Granadel, M. Prudhomme, B. Martin, J.-P. Claverys, Construction and evaluation of a chromosomal expression platform (CEP) for ectopic, maltose-driven gene expression in *Streptococcus pneumoniae*. *Microbiology* **152**, 343–349 (2006).

46. M. W. Davis, E. M. Jorgensen, ApE, a plasmid editor: A freely available DNA manipulation and visualization program. *Front. Bioinform.* **2**, 818619 (2022).

47. M. Akdel, D. E. V. Pires, E. P. Pardo, J. Jänes, A. O. Zalevsky, B. Mészáros, P. Bryant, L. L. Good, R. A. Laskowski, G. Pozzati, A. Shenoy, W. Zhu, P. Kundrotas, V. R. Serra, C. H. M. Rodrigues, A. S. Dunham, D. Burke, N. Borkakoti, S. Velankar, A. Frost, J. Basquin, K. Lindorff-Larsen, A. Bateman, A. V. Kajava, A. Valencia, S. Ovchinnikov, J. Durairaj, D. B. Ascher, J. M. Thornton, N. E. Davey, A. Stein, A. Elofsson, T. I. Croll, P. Beltrao, A structural biology community assessment of AlphaFold2 applications. *Nat. Struct. Mol. Biol.* **29**, 1056–1067 (2022).

48. H. Ashkenazy, S. Abadi, E. Martz, O. Chay, I. Mayrose, T. Pupko, N. Ben-Tal, ConSurf 2016: An improved methodology to estimate and visualize evolutionary conservation in macromolecules. *Nucleic Acids Res.* **44**, W344–W350 (2016).

49. T. Metsalu, J. Vilo, ClustVis: A web tool for visualizing clustering of multivariate data using principal component analysis and heatmap. *Nucleic Acids Res.* **43**, W566–W570 (2015).

50. K. Tamura, G. Stecher, S. Kumar, MEGA11: Molecular evolutionary genetics analysis version 11. *Mol. Biol. Evol.* **38**, 3022–3027 (2021).

51. A. Ducret, E. M. Quardokus, Y. V. Brun, MicrobeJ, a tool for high throughput bacterial cell detection and quantitative analysis. *Nat. Microbiol.* **1**, 16077 (2016).

52. P. V. Toukach, K. S. Egorova, Carbohydrate structure database merged from bacterial, archaeal, plant and fungal parts. *Nucleic Acids Res.* **44**, D1229–D1236 (2016).

53. S. Neelamegham, K. Aoki-Kinoshita, E. Bolton, M. Frank, F. Lisacek, T. Lütteke, N. O'Boyle, N. H. Packer, P. Stanley, P. Toukach, A. Varki, R. J. Woods, The SNFG Discussion Group, Updates to the symbol nomenclature for glycans guidelines. *Glycobiology* **29**, 620–624 (2019).

54. J. A. Lanie, W.-L. Ng, K. M. Kazmierczak, T. M. Andrzejewski, T. M. Davidsen, K. J. Wayne, H. Tettelin, J. I. Glass, M. E. Winkler, Genome sequence of Avery's virulent serotype 2 strain D39 of *Streptococcus pneumoniae* and comparison with that of unencapsulated laboratory strain R6. *J. Bacteriol.* **189**, 38–51 (2007).

55. K. M. Kazmierczak, K. J. Wayne, A. Rechtsteiner, M. E. Winkler, Roles of relSpn in stringent response, global regulation and virulence of serotype 2 *Streptococcus pneumoniae* D39. *Mol. Microbiol.* **72**, 590–611 (2009).

56. L.-T. Sham, K. R. Jensen, K. E. Bruce, M. E. Winkler, Involvement of FtsE ATPase and FtsX extracellular loops 1 and 2 in FtsEX-PcsB complex function in cell division of *Streptococcus pneumoniae* D39. *MBio* **4**, e00431-13 (2013).

57. A. K. Fenton, L. El Mortaji, D. T. Lau, D. Z. Rudner, T. G. Bernhardt, CozE is a member of the MreCD complex that directs cell elongation in *Streptococcus pneumoniae*. *Nat. Microbiol.* **2**, 16237 (2017).

58. Y. Li, C. M. Thompson, M. Lipsitch, A modified Janus cassette (Sweet Janus) to improve allelic replacement efficiency by high-stringency negative selection in *Streptococcus pneumoniae*. *PLOS ONE* **9**, e100510 (2014).

59. C. K. Sung, H. Li, J. P. Claverys, D. A. Morrison, An *rpsL* cassette, janus, for gene replacement through negative selection in *Streptococcus pneumoniae*. *Appl. Environ. Microbiol.* **67**, 5190–5196 (2001).
